# Three-Dimensional Single Molecule Localization Microscopy Reveals the Topography of the Immunological Synapse at Isotropic Precision below 15 nm

**DOI:** 10.1021/acs.nanolett.1c03160

**Published:** 2021-10-28

**Authors:** Lukas Velas, Mario Brameshuber, Johannes B. Huppa, Elke Kurz, Michael L. Dustin, Philipp Zelger, Alexander Jesacher, Gerhard J. Schütz

**Affiliations:** † Institute of Applied Physics, TU Wien, 1040 Vienna, Austria; ‡ Institute for Hygiene and Applied Immunology, Center for Pathophysiology, Infectiology and Immunology, Medical University of Vienna, 1090 Vienna, Austria; § Kennedy Institute of Rheumatology, University of Oxford, OX3 7FY Oxford, United Kingdom; ∥ Division for Biomedical Physics, Medical University of Innsbruck, 6020 Innsbruck, Austria

**Keywords:** T-cells, Immunological synapse, Interference Reflection Microscopy, T-cell receptor, 3D Single Molecule Localization Microscopy

## Abstract

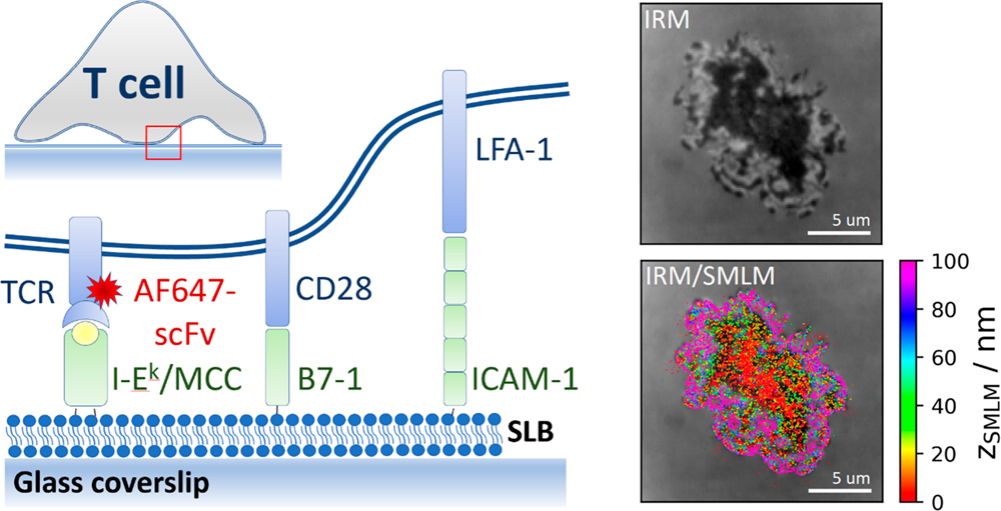

T-cells engage with antigen-presenting cells in search for antigenic peptides and form transient interfaces termed immunological synapses. Synapse topography affects receptor binding rates and the mutual segregation of proteins due to size exclusion effects. It is hence important to determine the 3D topography of the immunological synapse at high precision. Current methods provide only rather coarse images of the protein distribution within the synapse. Here, we applied supercritical angle fluorescence microscopy combined with defocused imaging, which allows three-dimensional single molecule localization microscopy (3D-SMLM) at an isotropic localization precision below 15 nm. Experiments were performed on hybrid synapses between primary T-cells and functionalized glass-supported lipid bilayers. We used 3D-SMLM to quantify the cleft size within the synapse by mapping the position of the T-cell receptor (TCR) with respect to the supported lipid bilayer, yielding average distances of 18 nm up to 31 nm for activating and nonactivating bilayers, respectively.

## Introduction

Understanding the topography of cellular interfaces is central for addressing many cell biological questions. The distance between the two juxtaposing cell surfaces not only regulates the affinity of protein–protein transinteractions,^1,2^ but the extension of the intercellular cleft also affects the spatial distribution of membrane proteins with differently sized ectodomains.^3^ A prominent example is the size-exclusion of the large phosphatase CD45 upon contact formation between a T-cell and an antigen-presenting cell (APC), which is suspected to represent an important regulatory mechanism for the phosphorylation of the T-cell receptor (TCR);^4^ according to this model, shifting the balance between Lck-mediated phosphorylation and CD45-mediated dephosphorylation induces downstream signaling.

A common way to study antigen-specific T-cell activation involves the use of functionalized glass-supported lipid bilayers (SLBs) as surrogates of APCs.^5−7^ This experimental design has several advantages when it comes to the application of high-resolution microscopy techniques while still preserving the essential hallmarks of T-cell signaling including the formation of an immunological synapse, the recruitment of the kinase ZAP-70 and other downstream signaling effectors, the increase in intracellular calcium, and the release of cytokines.

First, interfacing cells with a glass coverslip further allows for exploiting the interference of light reflected from the glass–water interface and light reflected from the cell membrane for imaging purposes.^8,9^ This technique, termed interference reflection microscopy (IRM), yields high precision information on the separation of the cell surface from the surface of the glass coverslip, eventually limited only by the signal-to-noise ratio of the data and by the knowledge of the interference model.^9^ IRM has been frequently applied to qualitatively assess the homogeneity of T-cell adhesion to activating or inert surfaces,^5,10−12^ often yielding patches of close contact next to areas of substantial elevation of the T-cell surface. Quantitative interpretation of the data, however, is often hampered by unknowns of the interference model.^9^ For example, tilted membranes or the presence of second or third order interferences are difficult to account for. In addition, multiple reflections from different layers of varying refractive index induce phase shifts in the IRM intensity profiles, thereby impeding absolute distance measurements. In fact, protein ectodomains and the glycocalyx contribute to the change in refractive index between the aqueous environment and the cell, rendering the plane of reflection rather poorly defined. Finally, IRM images are of diffraction-limited spatial resolution and thereby yield averages of the interference contrast over a few 100 nm. Fluctuations at smaller scales would hence be averaged out.

As a second advantage of hybrid synapses, they facilitate the application of total internal reflection (TIR) excitation to accentuate the signal of dyes proximal to the glass surface over intracellular background.^13^ Using TIR excitation, researchers discovered, for example, the formation of TCR microclusters upon T-cell activation.^14−16^

A third advantage relates to the use of single molecule localization microscopy (SMLM) for studying the organization of signaling molecules in the course of T-cell activation.^17−22^ Briefly, SMLM achieves superior spatial resolution by precisely localizing well-separated single molecule signals that can be obtained from blinking chromophores.^23^ Although a two-dimensional localization precision below 20 nm was frequently reported, it is difficult to achieve similar precision along the optical axis.^24^ In this context, the presence of the glass coverslip in the vicinity of the fluorophores of interest allows for using the supercritical angle fluorescence as a parameter for determining the distance between the dye molecule and the glass surface.^25,26^ Of note, the dye’s distance from the glass surface affects the shape of the recorded point spread functions due to different supercritical angle contributions. We have recently demonstrated that when combined with defocused imaging supercritical angle three-dimensional SMLM (3D-SMLM) achieves isotropic localization precision down to ∼10 nm in all three dimensions.^27^

Here, we used 3D-SMLM based on supercritical angle microscopy to study the topography of the immunological synapse formed between primary murine CD4^+^ T-cells and functionalized SLBs at isotropic localization precision below 15 nm. The obtained TCR localization maps were correlated with IRM images of the same synapse, thereby allowing not only cross-validation of the two approaches but also the identification of artifacts inherent to IRM images. From the TCR *z*-coordinates, we quantified the roughness of the T-cell surface within the synapse, as well as its separation from the SLB: Both for activating and nonactivating conditions we observed multiple TCR-proximal spots of close contact, which would qualify for CD45 exclusion. We finally quantitatively compared 3D-SMLM images with diffraction-limited TIR fluorescence microscopy of T-cell synapses to disentangle different contributions to the appearance of TCR microclusters.

## Results

### Correlative 3D-Single Molecule Localization Microscopy and Interference Reflection Microscopy

**Figure 1:**
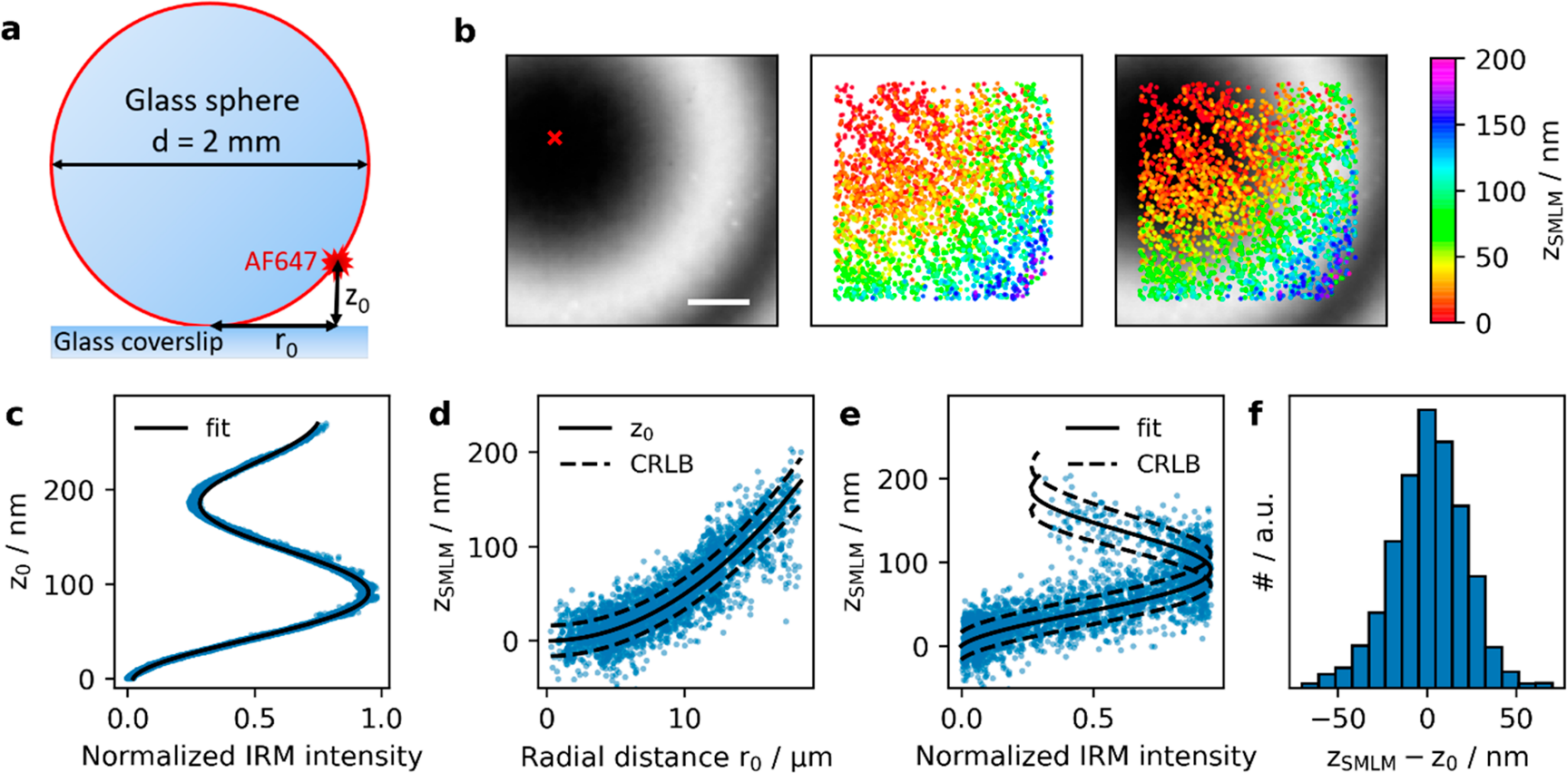
Control experiment on a fluorescently labeled glass sphere. A glass sphere of 2 mm diameter was labeled with BSA, Biotin–Streptavidin-AF647. On the basis of the known size of the sphere, the distance of the AF647 to the coverslip *z*_0_ was calculated from the measured radial distance *r*_0_ from the contact point of the sphere with the coverslip (a) (see SI Materials and Methods eq 1). (b) IRM and SMLM images were recorded next to the contact point of the sphere with the coverslip (red cross). (c) We plotted the *z*_0_ corresponding to the center of the respective pixel against the normalized IRM intensity and fitted with SI Materials and Methods eq 2. (d) The measured distances *z*_SMLM_ of all localizations were plotted against their radial distance *r*_0_. The solid line indicates the expected behavior for a spherical surface (SI Materials and Methods eq 1), dashed lines indicate the square root of the Cramér-Rao lower bound. (e) Dependence of *z*_SMLM_ on the normalized IRM intensity. Solid line indicates fit results from panel (c), dashed lines indicate the square root of the Cramér-Rao lower bound. (f) The difference between the measured values of *z*_SMLM_ and the calculated values of *z*_0_ showed a standard deviation of 21 nm. Scale bar 5 μm.

In order to evaluate the correlation between 3D-SMLM and IRM data, we sought a system with known separation of the detected single dye molecules from the glass surface. We opted for an AlexaF647-coated glass sphere of 1 mm radius adhered to a glass coverslip, which yielded *z*-distances of up to 150 nm within the field of view of 22 × 22 μm^2^ (Figure 1a). Figure 1b shows an IRM image recorded next to the contact point between the sphere and the glass surface. Concentric interference fringes are clearly visible. When plotting the recorded IRM intensity values versus the distance of the according pixels from the glass surface, *z*_0_, we observed the characteristic cosine-dependence (Figure 1c). In this case, three branches of the IRM intensity can be distinguished, corresponding to different orders of the interference pattern. The first branch covers *z*_SMLM_ values up to 90 nm, the second values of 90–180 nm, and the third values above 180 nm. A slight decrease in amplitude and wavelength of the recorded IRM curve is noted for increasing IRM interference orders, which is a consequence of reflections on the curved surface.^9^ In Figure 1b, we also included the recorded 3D-SMLM data, with the color-code indicating the calculated displacement from the glass surface, *z*_SMLM_. When plotted against the radial distance from the sphere’s contact point, *r*_0_, the measured single molecule displacements *z*_SMLM_ follow closely the surface of the sphere. (Figure 1d). We further correlated *z*_SMLM_ with the IRM intensity values recorded on the corresponding pixels (Figure 1e), yielding very good agreement of the two data sets. The solid black line shows the calibration curve obtained in Figure 1c, and the dashed lines indicate the expected Cramér-Rao lower bound (CRLB).^27,28^ To obtain a quantitative measure of the method’s *z*-precision, we calculated for each localization the difference between *z*_SMLM_ and the theoretical *z*_0_ (Figure 1f). The determined standard deviation of 21 nm agrees well with the Cramér-Rao lower bound of 17 nm.

### Correlative IRM, TIR, and 3D-SMLM of the Immunological Synapse

Next, we applied the method to image the three-dimensional topography of the immunological synapse formed between CD4^+^ murine 5c.c7 TCR-transgenic T-cells and stimulatory or inert surfaces. To visualize the position of the TCR, T-cells were labeled with an AlexaF647-conjugated single chain antibody fragment^6^ against the TCR β subunit. Before analysis, all 3D-SMLM images were corrected for overcounts, which eventually allowed us to obtain a valid estimation of the surface roughness (see Supporting Information (SI) Materials and Methods). An isotropic mean precision of 12.7, 12.0, and 14.6 nm was determined for single molecule localization along the *x-*, *y*-, and *z*-axis, respectively (SI Figure 1).

**Figure 2:**
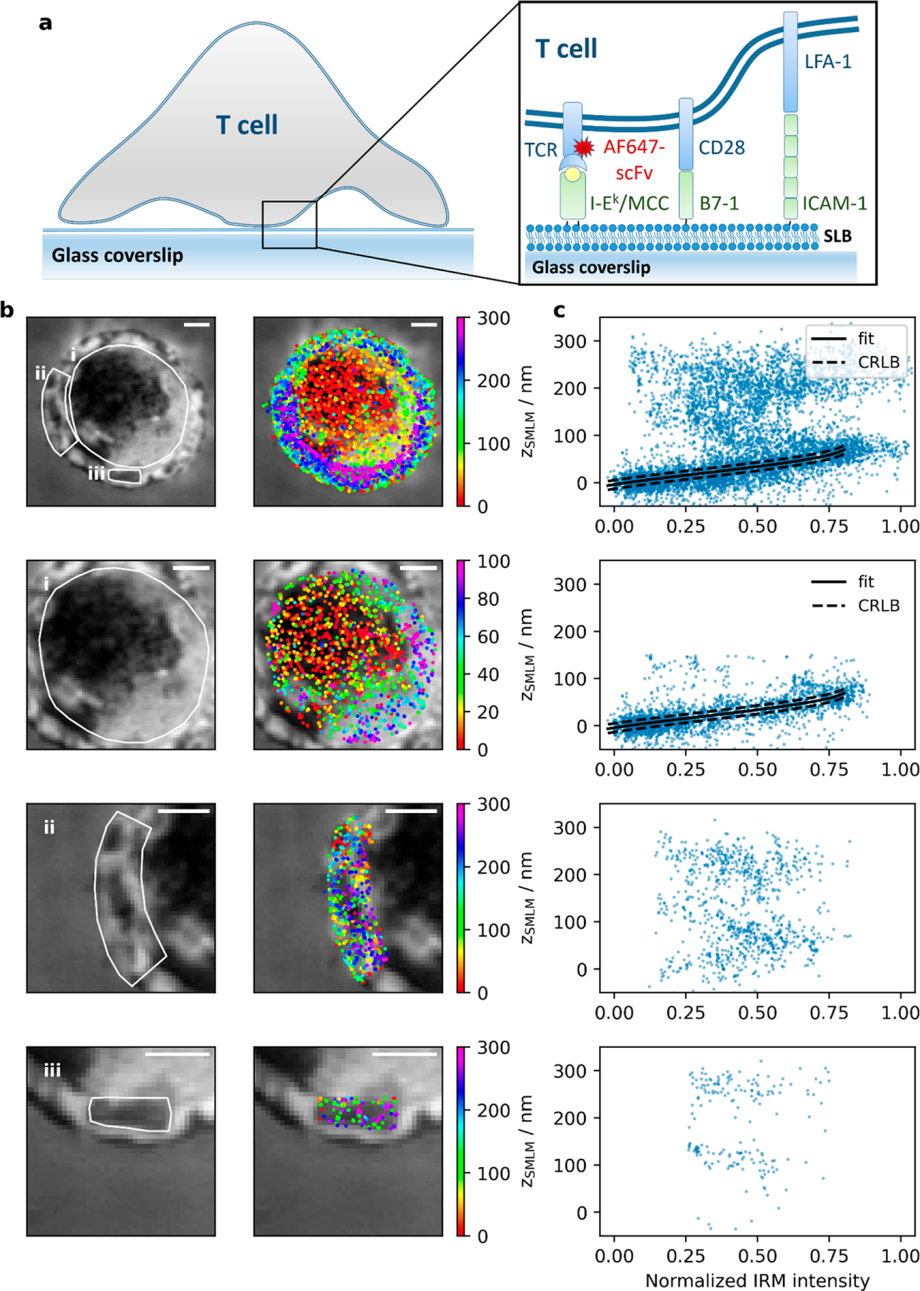
Correlative 3D-SMLM and IRM within the immunological synapse. (a) Experiments were performed on a T-cell adhering to an SLB functionalized with I-E^k^/MCC, B7-1 and ICAM-1; the TCR was labeled via AF647-conjugated H57-scFv. (b) IRM (left) and SMLM/IRM overlay images (right) of the immunological synapse. The areas i, ii, and iii are shown in magnification below. (c) Correlation plots between *z*_SMLM_ and the normalized IRM intensity for the areas indicated in panel b. Data points with *z*_SMLM_ < 100 nm were fitted with SI Materials and Methods eq 3 (solid line). The dashed lines indicate the square root of the Cramér-Rao lower bound. Scale bars 2 μm.

We first addressed the three-dimensional organization of the TCR in activated T-cells. To this end, T-cells were seeded onto fluid SLBs functionalized with MCC-loaded I-E^k^ at a surface density of 100 ± 30 molecules/μm^2^, which is known to stimulate intracellular calcium release;^29^ in addition, bilayers contained the adhesion molecule ICAM-1 and the costimulatory molecule B7-1 (Figure 2a). For all applied conditions, T-cell activation was controlled via ratiometric calcium imaging (SI Figure 3). Prior to conducting imaging experiments, T-cells were fixed at specific time points (here 5 min) after their seeding onto the SLBs and imaged by both via IRM and 3D-SMLM. The IRM images show substantial contrast fluctuations (Figure 2b), indicating corresponding fluctuations in the distance of the T-cell membrane from the SLB. This is supported by the 3D-SMLM data, where the determined TCR *z*-positions spread between 0 and 300 nm. Plotting *z*_SMLM_ versus the IRM intensity revealed a good correlation between the two data sets for the first branch of the IRM signal (Figure 2c, solid black line). This correlation vanished, however, for higher order IRM branches. We attribute this lack of correlation to additional parameters affecting the measured IRM contrast such as unknown angles of the reflecting surfaces or the occurrence of multiple interferences, which particularly disturbs IRM signals originating from reflections at larger distances from the glass surface. We therefore did not fit those regions of the IRM curves. In addition, different resolutions of the two methods impede direct comparison of the two data sets: while 3D-SMLM data report on *z*-distances specific for 2D coordinates that can be determined with a precision below the diffraction-limit, IRM images are limited by diffraction and hence provide average values over areas given by the size of the 2D point spread function.

For detailed assessment, we selected in region (i) of Figure 2b the cell center in which the left half of the region was well adhered to the SLB surface while the right half featured a separation of ∼60 nm from the SLB. Here, results obtained with the two imaging modalities are in very good agreement. In region (ii) showing the cell edge, where the T-cell formed a narrow lamellipodium, we observed two distinct clusters in the *z*_SMLM_ data, one reflecting the bottom, the other reflecting the top membrane of the lamellipodium. The two clusters were separated by ∼160 nm (SI Figure 2), which corresponded to the thickness of the lamellipodium.^30^ Interestingly, the *z*-positions of this particular region hardly correlated with the IRM patterns, likely due to high inclinations of the reflecting membrane at the lamellipodium edge possibly causing additional interferences in the IRM image. In region (iii), another feature of membrane topography became apparent: the central dark IRM area did not indicate an area of close contact but instead showed rather distal lamellipodium regions reflected by *z*_SMLM_ coordinates more than 100 nm away from the glass surface. Also in this region, the separation of the two lamellipodia membranes of ∼160 nm became apparent from two well-separated localization clusters in the *z*_SMLM_ data.

**Figure 3:**
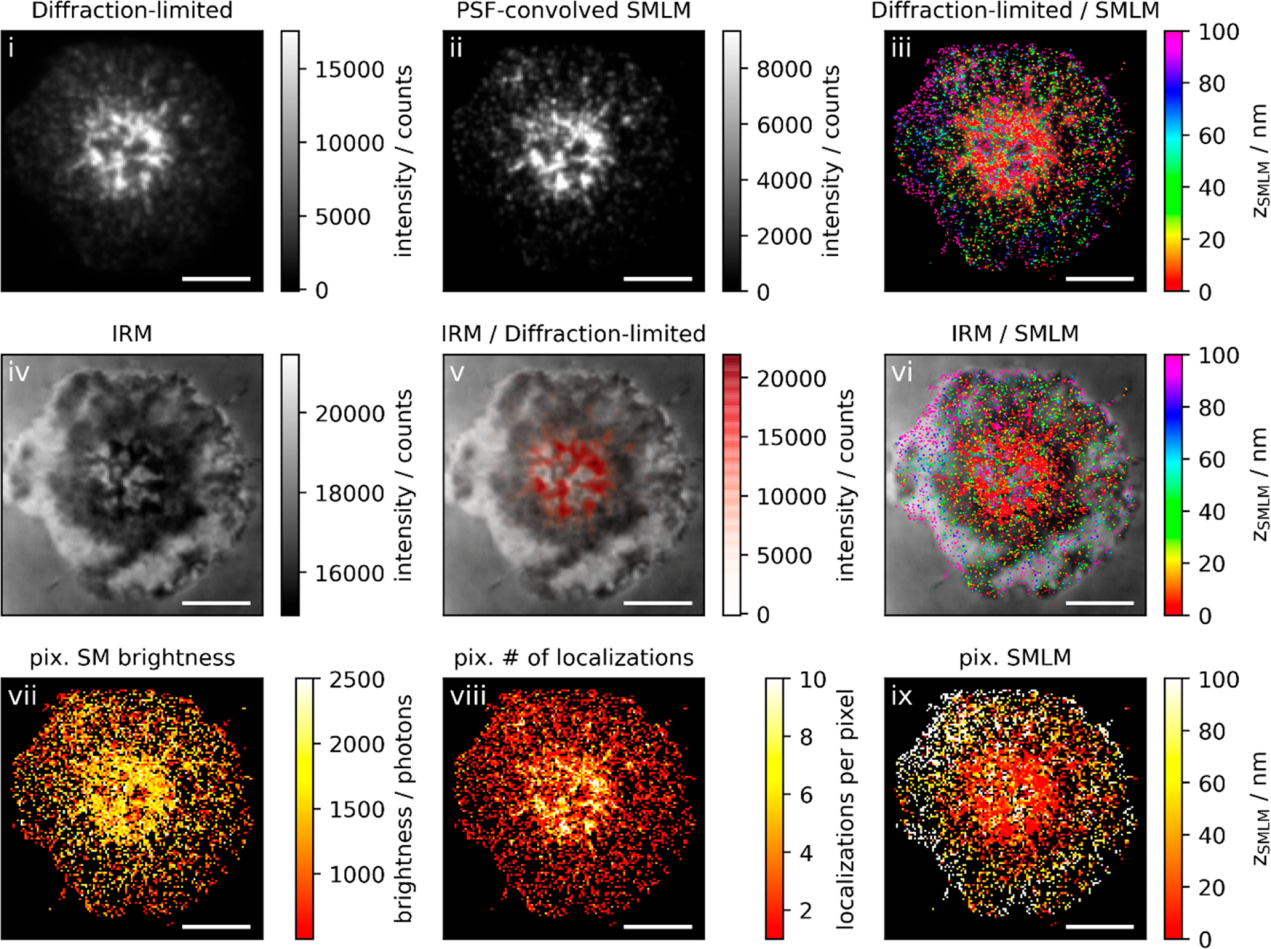
Correlative 3D-SMLM, IRM, and diffraction-limited TIR microscopy of the immunological synapse. T-cells were activated on a SLB functionalized with I-E^k^/MCC, B7-1, and high densities of ICAM-1 and fixed 10 min post seeding. The T-cell was imaged with IRM and fluorescence microscopy. (i) Diffraction-limited TIR image of the T-cell. (ii) Reconstruction of the diffraction-limited image by convolving the 3D-SMLM image with the corresponding psf. (iii) Overlay of the diffraction-limited TIR image with the 3D-SMLM image. The color-code indicates distances to the coverslip *z*_SMLM_. (iv) IRM image. (v) Overlay of the IRM image with the diffraction-limited image. (vi) Overlay of the IRM and the 3D-SMLM image. Bottom row images were generated by calculating the pixel-wise average of the 3D-SMLM images (pixel size of 146 nm is consistent with diffraction-limited image) according to pixelated mean single molecule (SM) brightness (vii), pixelated number of localizations (viii), and pixelated mean *z*_SMLM_ (ix). Scale bars: 5 μm.

It is also instructive to compare the obtained 3D SMLM and IRM images with conventional diffraction-limited TIR fluorescence microscopy images (Figure 3; see also SI supporting gallery Figures 1 and 2 for additional examples). In this particular example, the cell had been fixed just before the formation of the central supramolecular activation cluster (cSMAC), when TCR microclusters were observed in a ringlike structure around the cell center (Figure 3i). This image hence presumably reflects a snapshot of the directional microcluster transport toward the cSMAC.^31^ While TCRs in the ring itself showed tight contact with the glass surface, likely due to binding to the cognate pMHC, 3D-SMLM revealed that the engulfed circular membrane patch contained TCRs substantially elevated by ∼100 nm (Figure 3iii and Figure 3ix), in agreement with the observation that the cSMAC is a site of TCR endocytosis.^32^ In addition, some of the SLB engaged TCR in the cSMAC reside in ∼100 nm extracellular microvesicles that elevate the nonengaged TCR bearing plasma membrane by ∼100 nm above the SLB,^33^ potentially contributing to the two layers of TCR in the cSMAC.

We next analyzed TCR microclusters in more detail. In principle, TCR microcluster contrast is not only determined by protein enrichment but also by the closer proximity of the fluorophores to the glass surface, yielding both increased excitation intensity in the evanescent field as well as increased detection efficiency due to the collection of supercritical angle fluorescence. Because our method allows for disentangling single molecule fluorescence brightness, z-position, and local clustering, we addressed the different contributions to the diffraction-limited TIR images. For this, we compared the diffraction-limited TIR images (Figure 3i) with data obtained from 3D-SMLM. The single molecule brightness indeed showed some correlation with the positions of microclusters in the diffraction-limited image (Figure 3vii), which was also reflected in a map of the single molecule *z*-positions (Figure 3ix). Furthermore, single molecule localizations were strongly clustered at the positions of the microclusters (Figure 3viii). We sought to reconstruct the diffraction-limited image by convolving the single molecule localization map with the brightness-weighted point spread function (psf) (Figure 3ii). The reconstructed image agreed well with the original diffraction-limited image, down to the level of the individual TCR microclusters.

**Figure 4:**
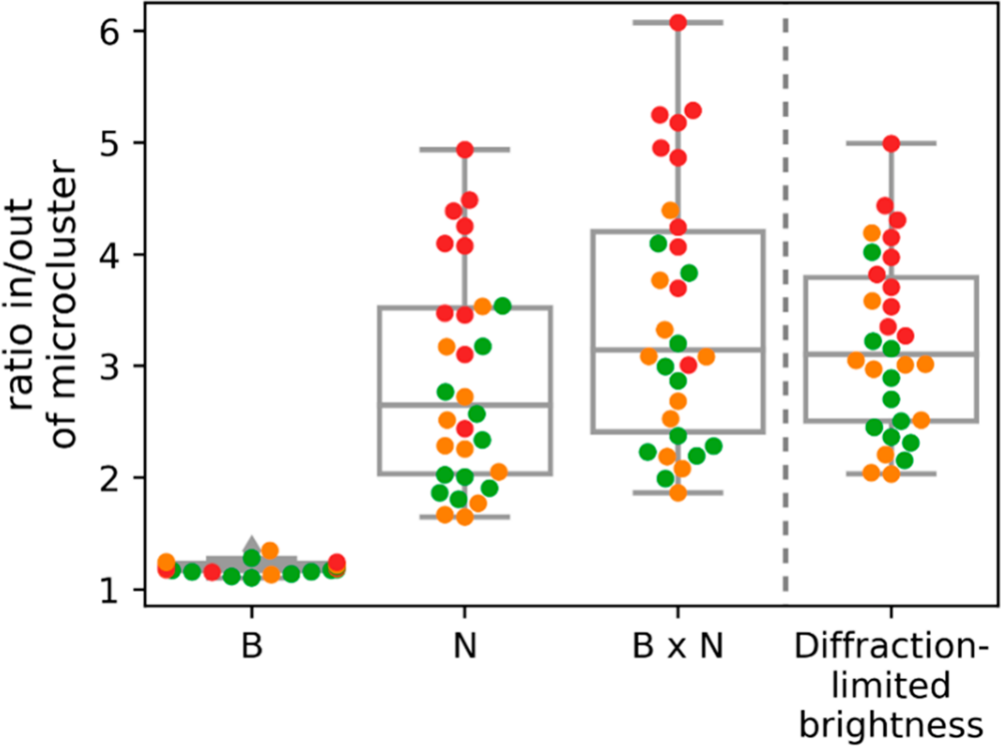
Disentangling single molecule brightness and molecular enrichment in TCR microclusters. We quantified the average single molecule brightness, *B*, and the number of localizations, *N*, in pixels corresponding to TCR microclusters (“in”) and the complementary regions of the synapse (“out”). Plotted are the ratios *in*/*out* for *B*, *N*, the product *B* × *N*, and the average diffraction-limited brightness per pixel. Each data point corresponds to the average ratio per cell. Colors indicate the time point of fixation post seeding (orange, 5–10 min; green, 10 min; red, 10–15 min). (*n* = 30 cells).

To quantitatively disentangle the different contributions, we identified TCR microclusters by intensity-thresholding the diffraction-limited images (SI Figure 6) and analyzed the single molecule properties separately for localizations coinciding with the TCR microcluster regions versus localizations outside of TCR microclusters. Figure 4 shows the ratios of single molecule brightness and the number of single molecule localizations per pixel, which were obtained from multiple cells. While the brightness levels in microclusters increased only by 20%, we observed about 3-fold enrichment of localizations. The product of the two ratios quantitatively matched the average brightness ratios of pixels corresponding to microclusters versus pixels of outside regions obtained from the diffraction-limited images. Importantly, as this analysis was based on pixel-wise discrimination of microclusters in the diffraction-limited images, it was not affected by residual overcounts in the SMLM images. Taken together, we conclude that the increased brightness of TCR microclusters in diffraction-limited images is mainly explained by enrichment of TCR molecules. Of note, we observed the expected increased brightness of microclusters fixed at late time-points (red data points) compared to those fixed at early time-points (orange data points);^16^ also, this effect is explained by an increase in the number of single molecule localizations.

We further studied T-cells contacting SLBs that were functionalized with the adhesion molecule ICAM-1 only, so that no calcium signal was triggered (see SI Figure 3 for ratiometric calcium analysis and SI supporting gallery Figures 3 and 4 for exemplary images). As expected, we did not observe the formation of TCR microclusters. While in general the cells spread well on such substrates, areas of close contact appeared more fragmented than in the activated situation. This effect was more pronounced when we reduced the density of the adhesion molecule ICAM-1 in the SLB from 125 ± 22 to 4 ± 2 molecules/μm ^2^.

### Quantitative Analysis of T-cell Surface Topography within the Immunological Synapse

**Figure 5:**
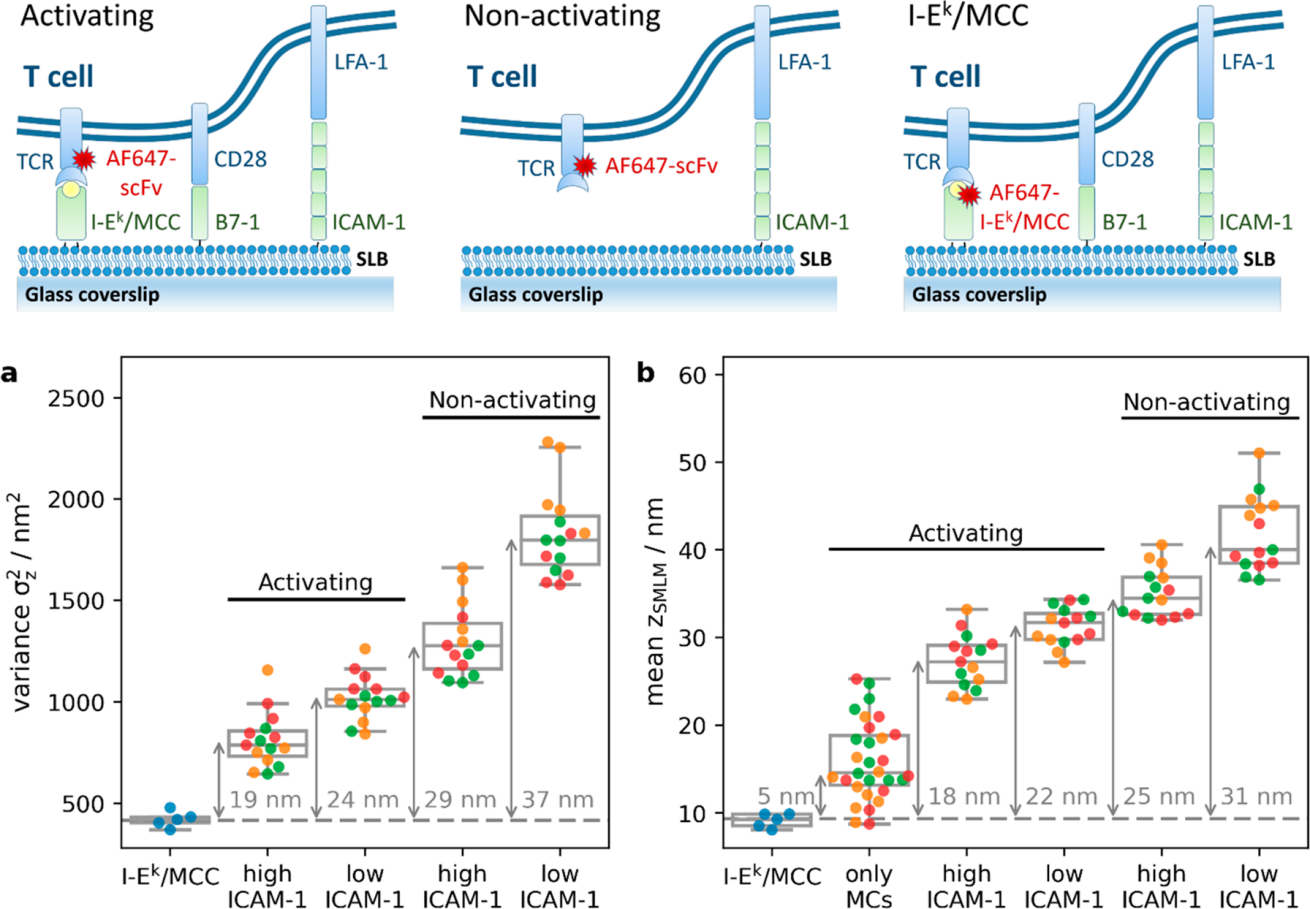
Contact analysis for T-cells recorded under activating and nonactivating conditions: T-cells were seeded on SLBs either functionalized with I-E^k^/MCC, B7-1, and ICAM-1 (termed “activating”) or with ICAM-1 only (termed “non-activating”). To vary adhesive strength, we used 125 ICAM-1 molecules per μm^2^ (termed “high ICAM-1”) or 4 ICAM-1 molecules per μm^2^ (termed “low ICAM-1”). T-cells were allowed to spread on SLBs and were fixed after 5–10 (orange), 10 (green), or 10–15 (red) minutes post seeding. As a control, unlabeled T-cells were seeded on SLBs containing I-E^k^/MCC labeled with AF647. The recorded localizations were filtered for *z*_SMLM_ < 100 nm in order to exclude contributions from the upper surface of lamellipodia. (a) The variance of *z*_SMLM_ per cell was plotted for different conditions. Arrows indicate the difference in variance to the control I-E^k^/MCC data, numbers indicate the corresponding square root. (b) Mean *z*_SMLM_ per cell was plotted for different conditions. Arrows indicate the difference with the control I-E^k^/MCC data. To calculate the mean distance of TCR microclusters from the glass surface we considered only localizations within microclusters (termed “only MCs”) in experiments performed both at high and low densities of ICAM-1 (*n* = 15 cells per condition).

For quantification, we determined the roughness of the T-cell surface within the immunological synapse. To prevent the inclusion of data originating from the top membrane of lamellipodia we only considered localizations with *z*_SMLM_ < 100 nm. At nonactivating conditions and low ICAM-1 densities, we observed substantial fluctuations of the recorded *z*-positions (Figure 5a). For quantitative determination of the surface roughness, we compared the obtained variances σ_*z*,TCR_^2^ with values obtained for fluorescently labeled SLB-anchored I-E^k^/MCC, σ_*z*,MHC_^2^, according to , yielding a standard deviation of σ = 37 nm. Fluctuations decreased to 29 nm when we increased the density of the adhesion molecule ICAM-1 in the lipid bilayer. Upon activation via higher densities of I-E^k^/MCC, T-cells adhered more smoothly to the surface, as indicated by reduced overall *z*-fluctuations of 19 nm. Also, in the case of activation we observed the T-cell surface flattening out to a considerable extent with ICAM-1 present at higher densities.

According to the kinetic segregation model, the axial dimension of the intercellular cleft determines the accessibility of the large phosphatase CD45. We hence quantified the absolute distance of the TCR from the SLB, when compared to fluorescently labeled SLB-anchored I-E^k^/MCC. Generally, we observed similar trends as for the standard deviations: with increasing densities of I-E^k^/MCC and ICAM-1 the TCR was observed to be closer to the SLB surface (Figure 5b). The separation varied between 18 nm at high densities of I-E^k^/MCC and ICAM-1 up to 31 nm for scanning T-cells recorded at low densities of ICAM-1. When selecting only signals corresponding to TCR microclusters for our distance analysis, we observed the expected close contact between TCR and I-E^k^/MCC with a calculated separation of 5 nm. The residual separation reflected in all likelihood the distance separating the dye site-specifically conjugated to the single chain antibody fragment and the dye coupled to the MCC peptide’s C-terminus as presented by I-E^k^.^6^

## Discussion

We applied here a novel 3D-SMLM method to map and analyze the position of the TCR within the immunological synapse at isotropic localization precision below 15 nm and put it in the context of IRM imaging. IRM contrast arises from interferences due to optical path-length differences between the beam reflected at the glass–water interface and beams reflected from surfaces within the sample. Recording 3D-SMLM localization maps is fundamentally different from IRM. Using supercritical angle detection combined with defocused imaging, our method essentially determines the three-dimensional position of all visible dye molecules. We employed here an AlexaF647-conjugated single chain antibody fragment that specifically recognizes the TCR β subunit.^6^ In previous studies, we have shown that labeling neither activates T-cells nor impedes specific antigen recognition.^6^ Of note, we used TIR excitation in order to confine imaging to the synapse region. While the evanescent field was narrow enough to prevent contributions from TCRs at the top of the T-cell, we could observe both the bottom and the top membranes of lamellipodia.

Indeed, there was in general a good qualitative agreement between the two imaging modalities. Substantial differences, however, arose when distal membrane regions contributed to the IRM contrast values. Then, contrast values did not unequivocally correspond to the axial membrane separation. This was especially observed in lamellipodia, where both the bottom and the top membrane contributed to the signal.

Two aspects of our study contain important immunological implications for understanding T-cell antigen recognition

### i What Is the Physical Reason for the Observation of TCR Microclusters?

In TIR excitation, fluorescent molecules close to the glass coverslip naturally contribute with higher brightness than distal molecules. This effect is further amplified by the collection of supercritical angle fluorescence when using high NA objectives.^25^ Taken together, what appears as a bright spot in diffraction-limited fluorescence microscopy could be the consequence of increased density or of increased brightness of the fluorophores. Given the close contact between TCR microclusters and the surface,^12^ researchers became concerned whether microclusters actually reflect TCR enrichment.

Three-dimensional SMLM provides ground truth information on the origin of apparent TCR microclusters in diffraction-limited TIR microscopy, as it allows for disentangling molecular enrichment from brightness changes. We observed only marginal contributions from single molecule brightness increase, as brightness was largely attributable to molecular enrichment. This finding is in accord with our observation of a rather smooth interface between the SLB and the T-cell membrane under activating conditions, so that there are globally only minor variations in the single molecule’s *z*-distance in comparison to the TIR penetration depth.

### ii Does the Observed Cleft Size Support CD45 Segregation?

A prominent model for TCR triggering involves the balance in the activities of the kinase lck and the phosphatase CD45 for ITAM phosphorylation. In this kinetic segregation model, proteins with bulky extracellular domains, such as the large phosphatase CD45, are proposed to be segregated locally from the comparably short pMHC-TCR complexes.^4^ Although there are ample reports that would be consistent with this model,^34−38^ the community has not reached a consensus yet.^39−41^

One difficulty has been the precise measurement of the cleft size between the T-cell surface and the opposing membrane. Given the dimension of the TCR-pMHC-CD4 ternary complex of 10 nm^42^ and our apparent distance measurements between TCR and I-E^k^/MCC within TCR microclusters of 5 nm, the obtained distance undervalues the cleft size by 5 nm. Upon correcting for this effect, an average cleft size within the whole synapse ranging between 23 and 36 nm for the conditions shown in Figure 5b can be estimated. In addition to the average cleft size, however, distance fluctuations within the synapse were of the same order (Figure 5a), indicating the presence of multiple contact sites between the two membranes. Assuming an axial length of CD45R0, the smallest CD45 isoform of approximately 22 nm,^34^ our measurements hence indicate the existence of numerous membrane contact sites that would be too narrow to host CD45, both for resting and activating conditions. In particular, an average cleft size of 30 nm together with an average surface roughness of 29 nm, as observed for scanning T-cells at high ICAM-1 densities, render the presence of multiple CD45 exclusion zones likely. Similar data were recently reported for the tips of microvilli which showed segregation of the TCR and CD45 prior to T-cell activation.^22^ Given that none of these scenarios promoted T-cell activation, it is possible that the ITAMs of these segregated TCRs are not accessible to kinases.^43^ In addition, TCR-CD45 segregation may be too transient to trigger stable phosphorylation of TCR ITAMs. Kinetic data about protein mobility in conjunction with the superresolution images would be needed in order to obtain a quantitative understanding of these key aspects of the TCR triggering process.

## Supporting Information Available

Additional supporting Figures 1–6; materials and methods including a description of the T-cell imaging experiment using supported lipid bilayers, an animal model and ethical compliance statement, as well as a description of the microscopy system, the performed data analysis, and the simulations; supporting gallery (PDF)
