# Supplementary material for: Three-Dimensional Single Molecule Localization Microscopy Reveals the Topography of the Immunological Synapse at Isotropic Precision below 15 nm
Source: Nano Lett. 2021 Oct 28;21(21):9247–55. doi: 10.1021/acs.nanolett.1c03160 (PMC8587899; doi:10.1021/acs.nanolett.1c03160)
Supplement: Supplementary file 1 — nl1c03160_si_001.pdf [file nl1c03160_si_001.pdf]

3D single molecule localization microscopy reveals the topography of the immunological synapse at isotropic precision below 15 nm

Lukas Velas[1], Mario Brameshuber[1], Johannes B. Huppa[2], Elke Kurz[3], Michael L. Dustin[3], Philipp Zelger[4], Alexander Jesacher[4], Gerhard J. Schütz[1,‡]

[1] Institute of Applied Physics, TU Wien, 1040 Vienna, Austria

[2] Institute for Hygiene and Applied Immunology, Center for Pathophysiology, Infectiology and Immunology, Medical University of Vienna, 1090 Vienna, Austria

[3] Kennedy Institute of Rheumatology, University of Oxford, OX3 7FY Oxford, UK

[4] Division for Biomedical Physics, Medical University of Innsbruck, 6020 Innsbruck, Austria

[‡] please send correspondence to: schuetz@iap.tuwien.ac.at

SUPPORTING FIGURES

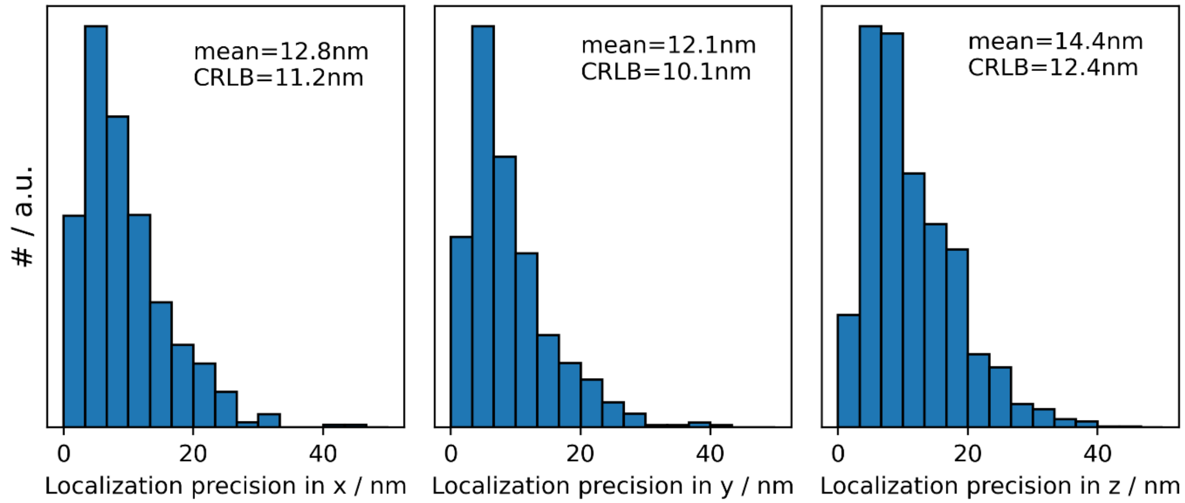

Supporting Figure 1: Histogram of localization precision in x, y and z direction. Localization precision was calculated as the standard deviation of localizations of the same molecule appearing in consecutive frames. The mean localization precision and the CRLB are indicated for the different dimensions.

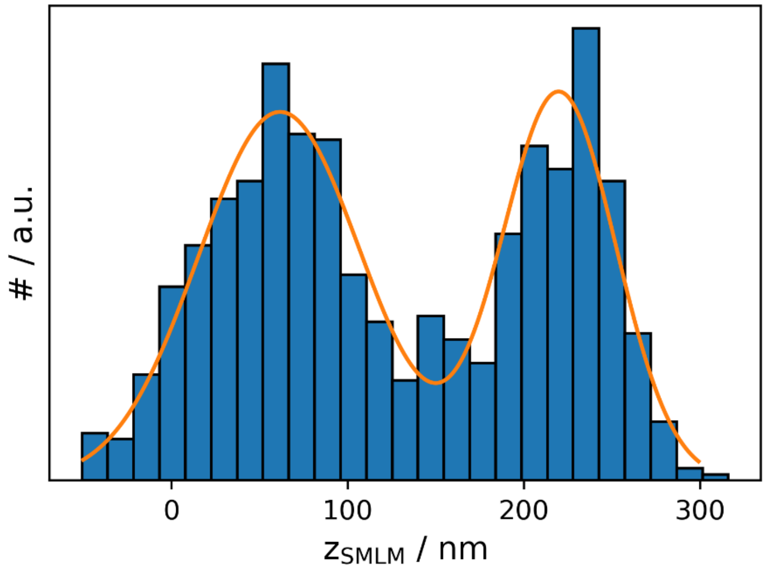

Supporting Figure 2: Histogram of z_{SMLM} positions in the area of the lamellipodium shown in **Fig. 2bii**, yielding 2 peaks at a mutual distance of 160 nm.

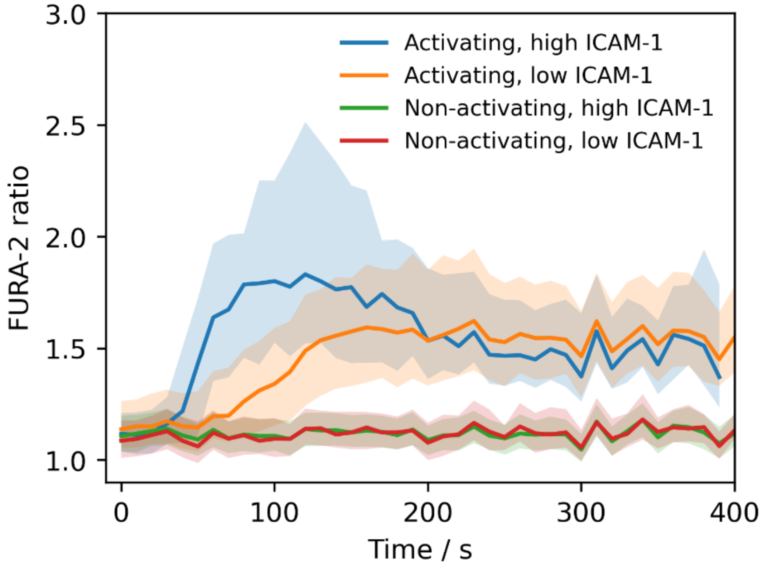

Supporting Figure 3: Release of intracellular calcium assessed via the median FURA-2 ratio. Data are shown as a function of time post seeding for T-cells subjected to activating and non-activating conditions while employing the ICAM-1 at indicated densities.

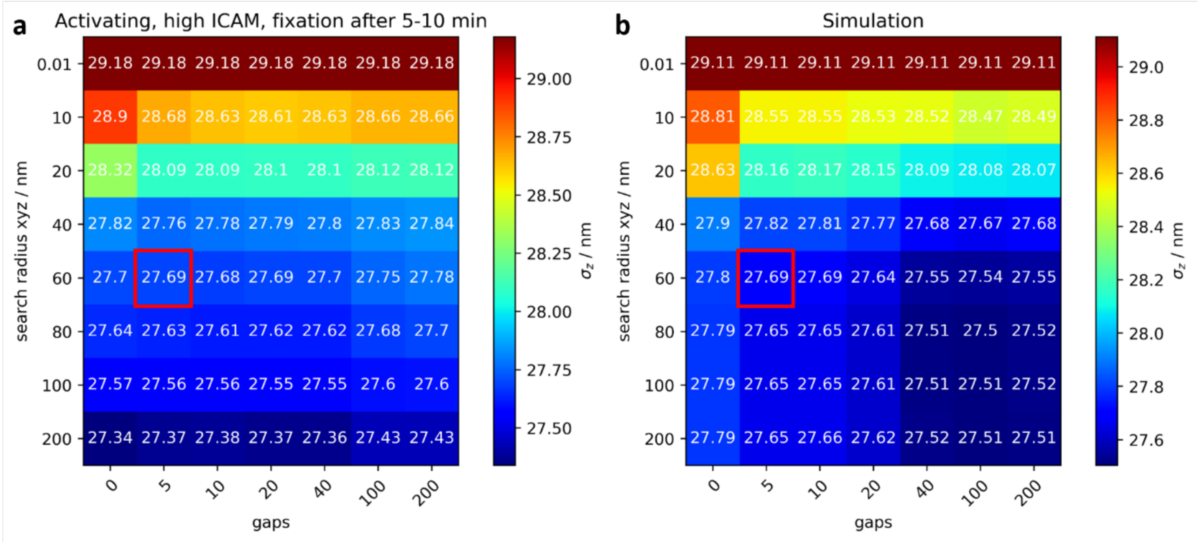

Supporting Figure 4: Selecting the appropriate parameters for correction of overcounts. **a)** 3D SMLM experiments were performed on T-cells recorded under activating conditions as described for experiments shown in **Fig. 3**. We determined the standard deviation of z_{SMLM}, σ_z, of all localization obtained after merging using the indicated three-dimensional search radius and maximally allowed gaps.

The chosen value is highlighted by the red square. **b)** For comparison, we analyzed simulated 3D-SMLM data via the same approach. The simulation was based on experimentally derived single molecule blinking statistics and localization precision (see Methods for details). For the simulations we assumed a surface roughness of 26nm.

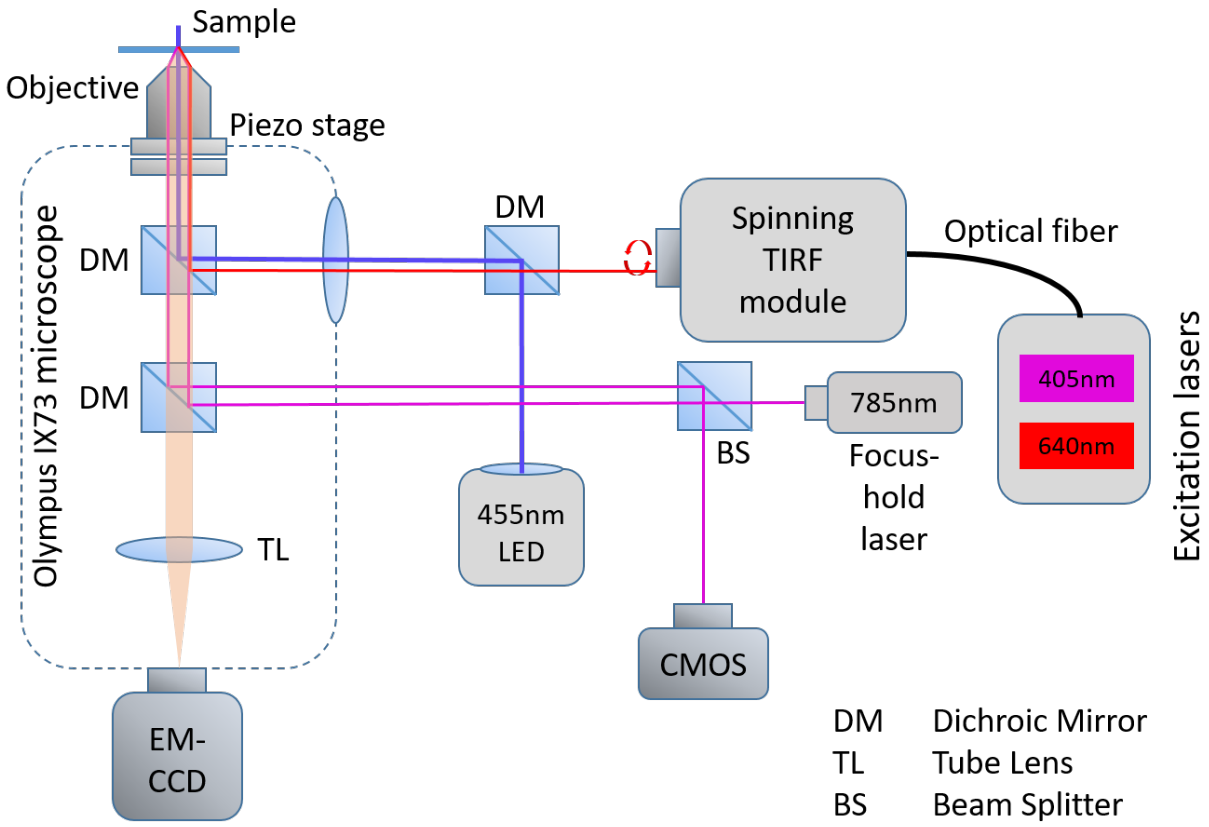

Supporting Figure 5: Scheme of the experimental setup.

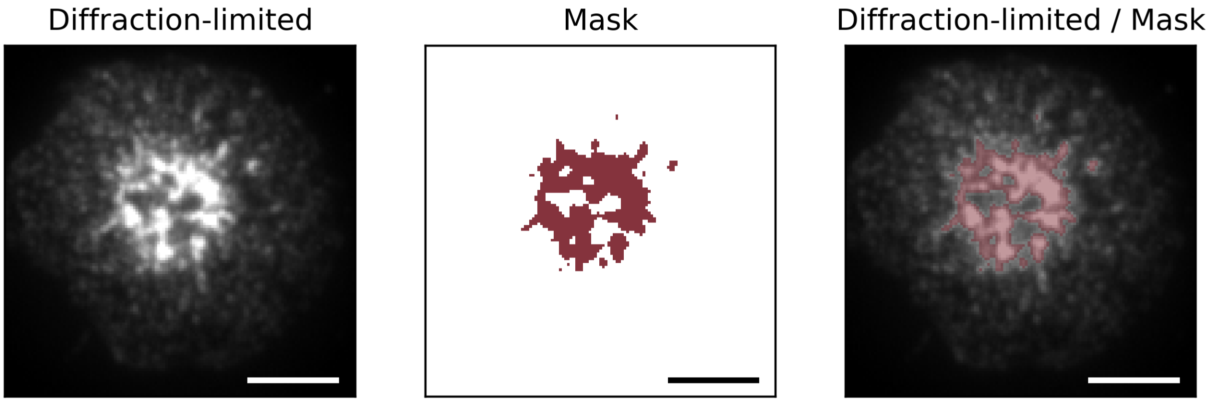

Supporting Figure 6: Intensity thresholding was applied to the diffraction-limited images of T-cells to identify TCR microclusters. Pixels with an intensity exceeding the mean intensity of the cell by a factor of 1.5 were considered as part of microclusters (“in”), all other pixels as outside of the microclusters (“out”). Scale bar 5 µm.

SUPPORTING MATERIALS AND METHODS

Reagents and proteins

Phosphate buffer saline (PBS), Hanks' balanced salt solution (HBSS), biotin labeled bovine albumin (BSA-biotin), fetal bovine serum (FCS), Histopaque-1119, glucose, glucose oxidase, catalase and cysteamine were purchased from Sigma Aldrich (Merck KGaA, Germany). 1,2-dioleoyl-sn-glycero-3-phosphocholine (DOPC), 1,2-dipalmitoyl-sn-glycero-3-phosphocholine (DPPC) and 1,2-dioleoyl-sn-glycero-3-[(N-(5-amino-1-arboxypentyl)iminodiacetic acid)succinyl] (nickel salt) (Ni-NTA-DGS) were from Avanti Polar Lipids, Inc. (USA). Streptavidin-AF647, IL-2 and formaldehyde were from Thermo Fischer scientific (USA). Cell medium RPMI 1640, penicillin, streptomycin, glutamate, sodium pyruvate, 1x non-essential amino acids and mercaptoenthanol were from Gibco, Life Technologies (USA). B7-1 and ICAM-1 were from Sino Biological (China). I-E^k and MCC peptide were prepared and labeled as described previously [1]. H57-scFv was expressed in inclusion bodies, refolded, site-specifically labeled with maleimide functionalized AF647 (Thermo Fisher Scientific, USA), and purified as described in [1].

Coating of glass spheres

Glass spheres (d=2mm, Schott, Germany) were plasma-cleaned (PDC-002, Plasma Cleaner Harrick Plasma, USA) for 10 minutes and incubated with 5 % BSA-biotin for 60 minutes. The spheres were then washed with PBS and incubated with 1 µg/ml Streptavidin-AF647 for 30 minutes. Afterwards, spheres were rinsed with PBS and placed into 8-well chambers (Nunc Lab-Tek, Thermo Scientific, USA) with plasma cleaned glass coverslip (MENZEL-Gläser Deckgläser 24 x 60 mm #1.5, Thermo Fisher Scientific, USA) for imaging.

Supported lipid bilayers

DOPC and Ni-NTA-DGS were dissolved in chloroform and mixed in a glass tube at a molar ratio of 98 (DOPC) : 2 (Ni-NTA-DGS), and subsequently dried under N_2 flow for 20 minutes. Next, the lipid mixture was resuspended in 1 ml of PBS and sonicated for 10 minutes (ultrasound bath USC500TH, VWR, England) to form small unilamellar vesicles (SUVs). Glass slides (MENZEL-Gläser Deckgläser 24 x 60 mm #1.5, Thermo Fisher Scientific, USA) were plasma cleaned for 10 minutes and glued on 8-well chambers. To form supported lipid bilayers, the freshly cleaned glass slides were incubated with SUVs for 20 min and washed extensively with PBS. Next, SLBs were incubated with His-tag proteins (Activating conditions: 10 ng I-E^k/MCC, 50 ng B7-1 and 30 ng or 0.3 ng ICAM-1; resting conditions: 30 or 0.3 ng ICAM-1) for 1 hour, and extensively rinsed with PBS. Before seeding the cells, PBS was exchanged for HBSS. Immobile SLBs were prepared identically, except for the following changes: we used a lipid mixture of 98 % DPPC and 2

% Ni-NTA-DGS. For sonication the ultrasound bath was preheated to 50 °C and coverslips were incubated with SUVs on a hot plate (65°C). Finally, SLBs were incubated with 10 ng of I-E^k/MCC-AF647.

T-cells

T-cells were obtained from transgenic 5c.c7 mice as described previously [2]. Briefly, T-cells were isolated from lymph nodes and stimulated with 2 µM HPLC-purified moth cytochrome C (MCC) peptide (ANERADLIAYLKQATK, Intavis Bioanalytical Instruments) in a T-cell medium (RPMI 1640 containing 10% FCS, 100 U/mL penicillin/streptomycin, 2mM glutamate, 1 mM sodium pyruvate, 1x non-essential amino acids, and 50 µM-Mercaptoenthanol). Culture volume was doubled and 100 U/mL IL-2 was added on day 2. On days 3 and 5, T-cell cultures were expanded in a ratio of 1:1. On day 6, dead cells were removed by centrifugation trough a Histopaque-1119 cushion. T-cell experiments were performed on days 7–9 after initial stimulation.

Animal model and ethical compliance statement

5c.c7 αβ TCR-transgenic mice bred onto the B10.A background were a kind gift from Michael Dustin (University of Oxford, UK). Both male and female mice at 8-12 weeks old were randomly selected and sacrificed for isolation of T-cells from lymph nodes and spleen, which was evaluated by the ethics committees of the Medical University of Vienna and approved by the Federal Ministry of Science, Research and Economy, BMWFW (BMWFW-66.009/0378-WF/V/3b/2016). Animal husbandry, breeding and sacrifice of mice was performed in accordance to Austrian law (Federal Ministry for Science and Research, Vienna, Austria), the guidelines of the ethics committees of the Medical University of Vienna and the guidelines of the Federation of Laboratory Animal Science Associations (FELASA), which match those of Animal Research: Reporting in vivo Experiments (ARRIVE). Further, animal husbandry, breeding and sacrifice for T-cell isolation was conducted under Project License (I4BD9B9A8L) which was evaluated by the Animal Welfare and Ethical Review Body of the University of Oxford and approved by the Secretary of State of the UK Home Department. They were performed in accordance to Animals (Scientific Procedures) Act 1986, the guidelines of the ethics committees of the Medical Science of University of Oxford and the guidelines of the Federation of Laboratory Animal Science Associations (FELASA), which match those of Animal Research: Reporting in vivo Experiments (ARRIVE).

TCR staining for imaging

In order to label the T-cell receptor, appr. 10^6 cells were washed twice by centrifugation (300 RCF for 3 min) with 2 ml of HBSS containing 2% FCS. Next, T-cells were incubated with H57-scFv conjugated with

AF647 at saturating conditions for 20 min on ice. After incubation the cells were washed twice by centrifugation with 2 ml of HBSS + 2% FCS at 4°C. The cells were then immediately seeded on the prepared SLBs and fixed after 5-15 minutes of spreading with 4% formaldehyde + 0.2% glutaraldehyde (Serva, Germany) for 10 min. Afterwards, the fixing solution was rinsed with PBS. Finally, PBS was exchanged with blinking buffer for dSTORM (PBS (pH 7.4), 10% glucose, 500 µg/ml glucose oxidase, 40 µg/ml catalase and 50 mM cysteamine) and cells were imaged immediately.

Microscopy

The home-built experimental setup was based on an Olympus IX73 (Japan) microscope body equipped with a high NA objective (Carl Zeiss, alpha-plan apochromat, 1.46 NA, 100x, Germany) (**Supporting Fig. 5**). Samples were illuminated with 640 nm laser light (100 mW nominal laser power, OBIS Laser box, Coherent, USA) coupled by an optical fiber into a high-speed galvo back focal scanner (iLas^2, Visitron Systems, Germany). The iLas^2 system was used for rapid spinning of the laser in the back focal plane of the objective (spinning TIR configuration), yielding an excitation intensity of 1 kW/cm^2 at the sample. Finally, lasers were reflected to the specimen and filtered from the fluorescence signal by a quad dichroic mirror (Di01-R405/488/532/635, Semrock, USA) and an emission filter (ZET405/488/532/642m, Chroma, USA) placed in the upper deck of the microscope body. The same dichroic mirror was used for coupling in light from a blue LED (455 nm, Thor Labs, USA) for IRM imaging. An additional shortpass beamsplitter (HC BS 750 SP, Semrock, USA) was placed in the lower deck of the microscope for coupling in a home-built focus hold system based on a 785 nm laser diode (LM9LP, Thor Labs, USA), a camera (ac640-750um, Basler, Germany) and an objective piezo stage (P721.SL2, Physik Instrumente, Germany). Finally, the specimen was imaged on a camera (EM-CCD Ixon Ultra, Andor, UK). Illumination and image acquisition was operated by VisiView (Visitron Systems, Germany).

We used defocused imaging for determining the three-dimensional single molecule position, as described previously (Zelger et al 2020). Briefly, we used dSTORM to record spatially separated single molecule signals of the fluorescently labeled T-cells. Before the experiment, single molecules adhered to the glass coverslip next to the cells were brought into focus, and the objective was displaced by 500 nm towards the sample by the piezo stage (P721.SL2, Physik Instrumente, Germany). For the dSTORM experiment, 20.000 frames at 20 ms illumination time and 9 ms delay time were recorded for each analyzed image. After 10.000 frames a 405 nm UV laser was added to the illumination sequence (1 mW nominal laser power, OBIS Laser box, Coherent, USA). The distance of the objective to the coverslip was maintained by the focus hold system for the entire recording of the cells.

Data Analysis

Localization of single molecules: The single molecule coordinates x, y, and z, the single molecule brightness (B) and the background signal (bg) were determined by maximum likelihood fitting of the calculated psf model on a 21 by 21 pixel subregion around the signal, as previously described [3]. The psf model includes optical aberrations of the experimental setup retrieved from z-stack measurements of 100 nm fluorescent beads (TetraSpeck Microspheres, Thermo Fisher Scientific, USA). The defocus value was adjusted in the software so that the lowest localizations in z appeared close to 0. Single molecule signals closer than 1500 nm were discarded in order to avoid psf overlaps.

SMLM corrections: It is necessary to correct for field-dependent tilt aberrations, as they would introduce bias into the determined z-positions of the localized signals. For this purpose, we imaged immobile supported lipid bilayers functionalized with I-E^k/MCC AF647 as a ground truth sample, in which all dye molecule should be located in the same z-plane. The localizations were fitted with a planar surface, which was then subtracted from all localizations for correction. In order to correct for overcounting artifacts, localizations were merged by a tracking algorithm (Trackpy) [4], yielding the average three-dimensional position and the average single molecule brightness. Parameters of Trackpy were the three-dimensional search radius, and the maximally allowed size of gaps within a given trajectory which may occur due to molecular blinking or fitting problems. Both parameters should be chosen sufficiently large to include most blinks originating from the same molecule, and sufficiently small to avoid connecting blinks from different molecules. To identify an appropriate choice of the three-dimensional search radius and the maximally allowed gap size, we analyzed the standard deviation of all determined z-coordinates σ_z. Beyond a search radius of 60 nm and a gap size of 5 we observed a plateau in σ_z (**Supporting Fig. 4a**); in the following we hence analyzed all data with a search radius of 60 nm and a gap size of 5. To find out whether the obtained values of σ_z correspond to the underlying surface roughness we simulated 3D-SMLM experiments (**Supporting Fig. 4b**), using experimentally derived single molecule blinking statistics and localization precision. For a simulated surface roughness of 26 nm we found high quantitative agreement between our simulations and data recorded on T-cells seeded on activating supported lipid bilayers. For the selected values of 60 nm search radius and 5 gaps we overestimated the surface roughness only by 1.5 nm. Finally, localizations were filtered for the single molecule brightness B ($500 < B < 4000$) in order to avoid fitting of camera noise or of strong signals arising from overlapping molecules.

Data recorded on a glass sphere:

First, the contact point of the sphere to the glass coverslip was determined by fitting a 2D second-order polynomial to the IRM intensity profile in **Fig.1b** between the central intensity minimum and the first intensity maximum. The expected distance z_0 of the glass sphere from the coverslip was calculated via

$$z_0 = R - \sqrt{R^2 - {r_0}^2} \qquad \text{(Eq. 1)}$$

where $R = 1$ mm is the radius of the sphere and r_0 is the radial distance to the contact point. The dependence of the normalized IRM intensity on the expected distance of the sphere to the coverslip z_0 was fitted with

$$IRM = \alpha_1 e^{-\alpha_2 z_0} \cos\left(2\pi \frac{z_0}{\alpha_3}\right) + \alpha_4 \qquad \text{(Eq. 2)}$$

yielding $\alpha_1 = -0.54$, $\alpha_2 = 0.0036$ nm^{-1}, $\alpha_3 = 188{,}42$ nm, $\alpha_4 = 0.56$.

For **Fig. 1d** we put the origin of the z-coordinates to the surface of the glass coverslip, to which we referenced all obtained z-positions. In **Fig.1e** the distance of the localizations to the coverslip z_{SMLM} was correlated with the IRM intensity of the corresponding pixels and overlaid with the fit from Eq. 2. The Cramér-Rao lower bounds shown as dashed lines were calculated considering the localizations' brightness, background level, z position and the psf model including aberrations.

Data recorded on T-cells: All z-positions were referenced against z-positions determined from signals observed around the cells, which correspond to traces of fluorescent H57-scFv molecules adsorbed to the glass surface.

In **Fig.2c**, localizations were plotted versus the IRM intensity of the corresponding pixels and the localizations with $z_{SMLM} < 100$ nm were fitted with

$$z_{SMLM} = \frac{\beta_1}{2\pi} arccos\left(\frac{IRM}{\beta_2} - \beta_3\right) \qquad \text{(Eq. 3)}$$

yielding $\beta_1 = -0.50$, $\beta_2 = 225.69$ nm, $\beta_3 = 0.39$.

To determine the localization precision for x, y and z direction (**Supporting Fig. 1**) we first calculated the standard deviation of localizations from signals occurring in subsequent frames within a three-dimensional distance of 60 nm and a maximum gap size of 5, yielding the single frame localization precision $\sigma_{1,i}$ for molecule i. Merged localizations show an improved localization precision $\sigma_{2,i} = \sigma_{1,i}/\sqrt{N_i}$, with N_i the number of observations per molecule. The overall localization precision is then given by $\sigma = \sqrt{\alpha \cdot \langle \sigma_{1,i}^2 \rangle_i + (1-\alpha) \cdot \langle \sigma_{2,i}^2 \rangle_i}$, where α denotes the fraction of molecules observed only once. CRLBs were calculated from the estimates for each single molecule observation, $CRLB_i$, accordingly. The histograms in **Supporting Fig. 1** show the weighted sums of the normalized histograms of $\sigma_{1,i}$ and $\sigma_{2,i}$.

The z histogram of the lamellipodium shown in Fig. 2b (ii) was fitted with a sum of 2 gaussian functions with center positions at 68 and 228 nm (**Supporting Fig. 2**).

To reconstruct diffraction-limited images from SMLM recordings (**Fig. 3ii**), localizations were convolved with the psf model taking into account the single molecule brightness B and the z position. The psf model was calculated for a focus position at z = 0 and included the aberrations of our optical setup.

For specific analysis of TCR microclusters, we ascribed pixels to TCR microclusters based on intensity thresholding of the diffraction limited images of activated T-cells (threshold: MC intensity per pixel > 1.5-times mean intensity per pixel of the whole cell) (see **Supporting Fig. 6** for an example). Localizations were assigned either to microcluster pixels (termed "in") or to the complementary pixels (termed "out"). The ratio in/out of the single molecule brightness values was calculated based on the single molecule brightness B. The density ratio was calculated from counting the number of localizations, N, per IN and OUT area.

Simulations

To produce a continuous, rough surface we first generated a random 3D surface on a 10 nm grid with a size of 10 x 10 µm^2 by placing normally distributed z-coordinates on every grid-point; the normal distribution was centered around zero with a standard deviation of 26 nm. Next, a Gaussian smoothing with a standard deviation of 0.5 µm (in x/y) was applied, and the z-coordinates were rescaled to preserve a standard deviation of 26 nm in z. Finally, the resulting surface was shifted along the z-axis so that the lowest point of the surface yielded z=0 nm. Onto this surface, we placed 1000 molecules randomly distributed in the x/y plane. For each molecule a blinking sequence of on- and off-states was generated (total of 20000 frames), using the single molecule blinking statistics determined by analyzing the blinking

kinetics of AF647 in underlabeled T-cells. We observed a slight z-dependence of the mean on-time $\bar{t}_{on} = k_1 + k_2 \cdot z_{SMLM}$, with $k_1 = 1.5\,frames$ and $k_2 = 0.01\,frames/nm$, which was taken into account by including each molecule's z-position; the off-time $\bar{t}_{off} = 5000 frames$ was largely independent of the z-coordinate. Both on- and off-times were assigned using exponential distributions with the corresponding mean values $\bar{t}_{on}$ and $\bar{t}_{off}$. Localizations were assigned to each molecule's position in the on-periods according to the determined localization errors of 12 nm in x and y direction. In case of localization errors along the z-direction we took into account the decreased excitation along the optical axis upon using TIR excitation, which was determined from underlabeled cells; we obtained $\sigma_z = k_3 + k_4 \cdot z_{SMLM}$, with $k_3 = 12\,nm$ and $k_4 = 0.05$. The final data set was used for merging analysis via Trackpy with the different search radii and gap sizes (see **Supporting Fig. 4**).

SUPPORTING REFERENCES

1. Huppa, J. B.; Axmann, M.; Mortelmaier, M. A.; Lillemeier, B. F.; Newell, E. W.; Brameshuber, M.; Klein, L. O.; Schütz, G. J.; Davis, M. M. *Nature* **2010,** 463, (7283), 963-967.
2. Huppa, J. B.; Gleimer, M.; Sumen, C.; Davis, M. M. *Nat Immunol* **2003,** 4, (8), 749-55.
3. Zelger, P.; Kaser, K.; Rossboth, B.; Velas, L.; Schütz, G. J.; Jesacher, A. *Optics Express* **2018,** 26, (25), 33166-33179.
4. Allan, D. B.; Caswell, T.; Keim, N. C.; van der Wel, C. M.; Verweij, R. W. *Zenodo* **2021**, http://doi.org/10.5281/zenodo.4682814.

SUPPORTING GALLERY

Figure 1a: Activating conditions, high ICAM-1 density, fixation: 5-10 min post seeding 14

Figure 1b: Activating conditions, high ICAM-1 density, fixation: 10 min post seeding 20

Figure 1c: Activating conditions, high ICAM-1 density, fixation: 10-15 min post seeding 26

Figure 2a: Activating conditions, low ICAM-1 density, fixation: 5-10 min post seeding 32

Figure 2b: Activating conditions, low ICAM-1 density, fixation: 10 min post seeding 38

Figure 2c: Activating conditions, low ICAM-1 density, fixation: 10-15 min post seeding 44

Figure 3a: Non-activating conditions, high ICAM-1 density, fixation: 5-10 min post seeding 50

Figure 3b: Non-activating conditions, high ICAM-1 density, fixation: 10 min post seeding 56

Figure 3c: Non-activating conditions, high ICAM-1 density, fixation: 10-15 min post seeding 62

Figure 4a: Non-activating conditions, low ICAM-1 density, fixation: 5-10 min post seeding 68

Figure 4b: Non-activating conditions, low ICAM-1 density, fixation: 10 min post seeding 74

Figure 4c: Non-activating conditions, low ICAM-1 density, fixation: 10-15 min post seeding 80

Figure 1a: Activating conditions, high ICAM-1 density, fixation: 5-10 min post seeding

Correlative 3D-SMLM, IRM, and diffraction-limited TIR microscopy of the immunological synapse. T cells were activated on an SLB functionalized with I-E^k/MCC, B7-1 and high density of ICAM-1, and fixed 5-10 minutes post seeding. The T cell was imaged with IRM and fluorescence microscopy: (i) Diffraction-limited TIR image of the T cell. (ii) Reconstruction of the diffraction-limited image by convolving the 3D-SMLM image with the corresponding psf. (iii) Overlay of the diffraction-limited TIR image with the 3D-SMLM image. Color-code indicates distance to the coverslip z_{SMLM}. (iv) IRM image. (v) Overlay of the IRM image with the diffraction-limited image. (vi) Overlay of the IRM and the 3D-SMLM image. Bottom row images were generated by calculating the pixel-wise average of the 3D-SMLM images (pixel size of 146nm is consistent with diffraction-limited image) according to pixelated mean single molecule (SM) intensity (vii), pixelated number of localizations (viii) and pixelated mean z_{SMLM} (ix). Scale bars 5 µm.

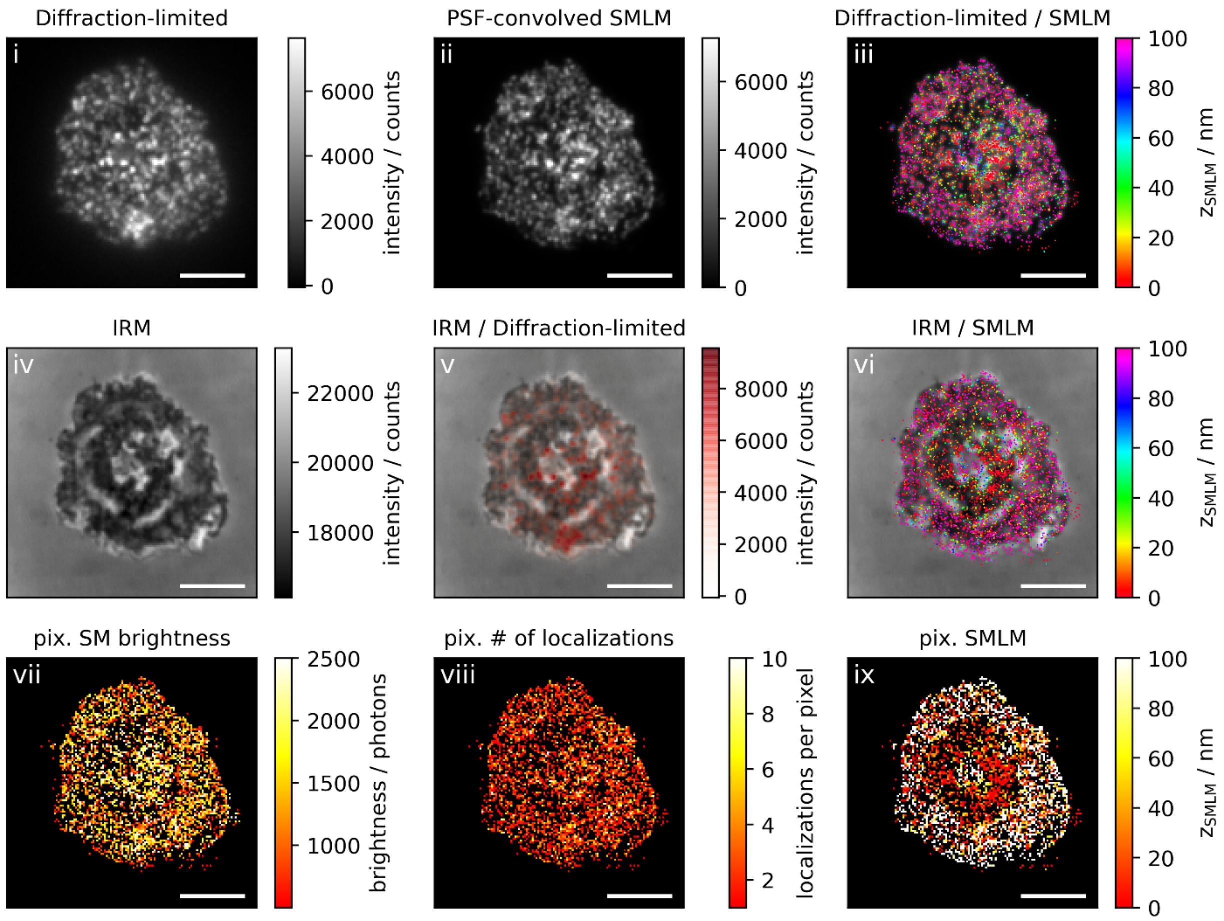
Diffraction-limited
i
intensity / counts
PSF-convolved SMLM
ii
intensity / counts
Diffraction-limited / SMLM
iii
z_{SMLM} / nm
IRM
iv
intensity / counts
IRM / Diffraction-limited
v
intensity / counts
IRM / SMLM
vi
z_{SMLM} / nm
pix. SM brightness
vii
brightness / photons
pix. # of localizations
viii
localizations per pixel
pix. SMLM
ix
z_{SMLM} / nm

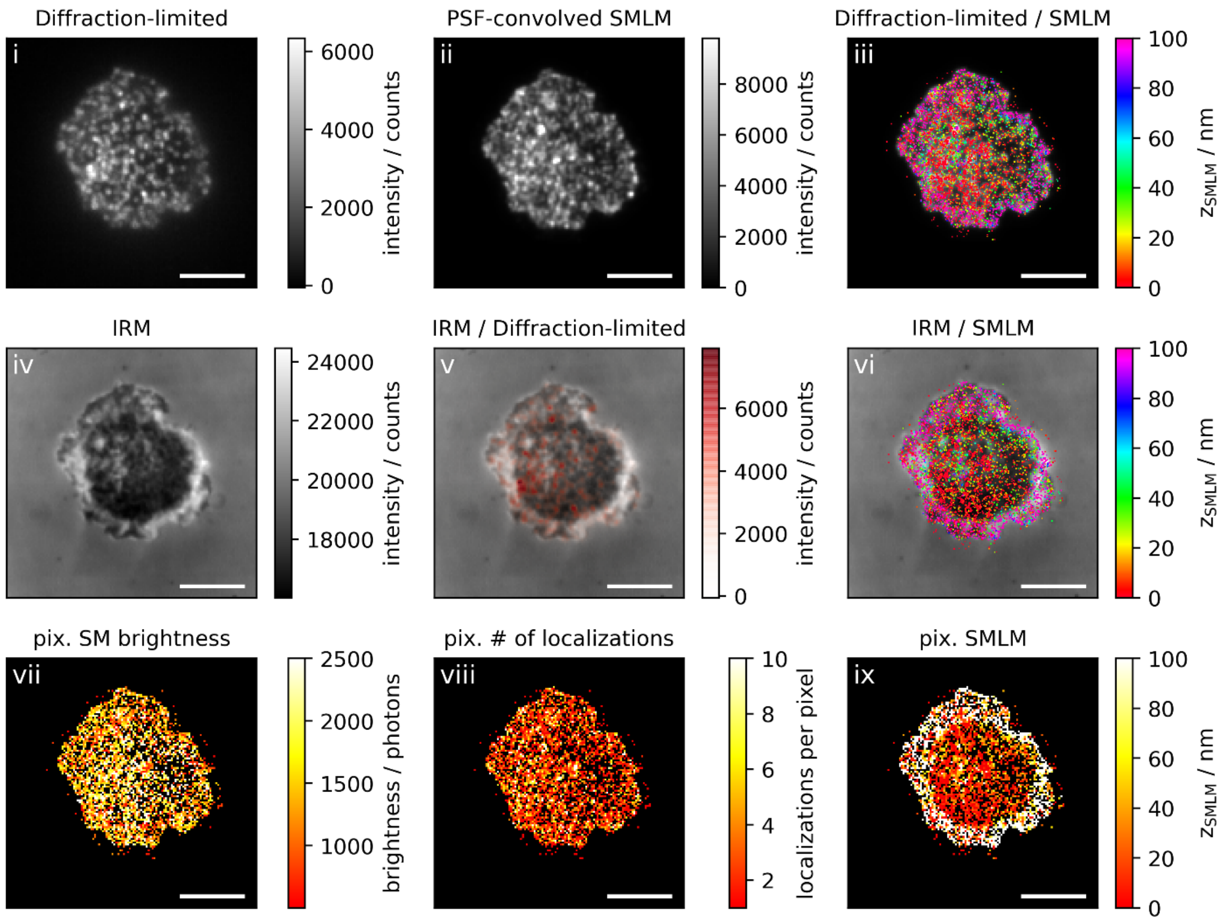

Diffraction-limited
i
6000
4000
2000
0
intensity / counts
PSF-convolved SMLM
ii
8000
6000
4000
2000
0
intensity / counts
Diffraction-limited / SMLM
iii
100
80
60
40
20
0
z_{SMLM} / nm
IRM
iv
24000
22000
20000
18000
intensity / counts
IRM / Diffraction-limited
v
6000
4000
2000
0
intensity / counts
IRM / SMLM
vi
100
80
60
40
20
0
z_{SMLM} / nm
pix. SM brightness
vii
2500
2000
1500
1000
brightness / photons
pix. # of localizations
viii
10
8
6
4
2
localizations per pixel
pix. SMLM
ix
100
80
60
40
20
0
z_{SMLM} / nm

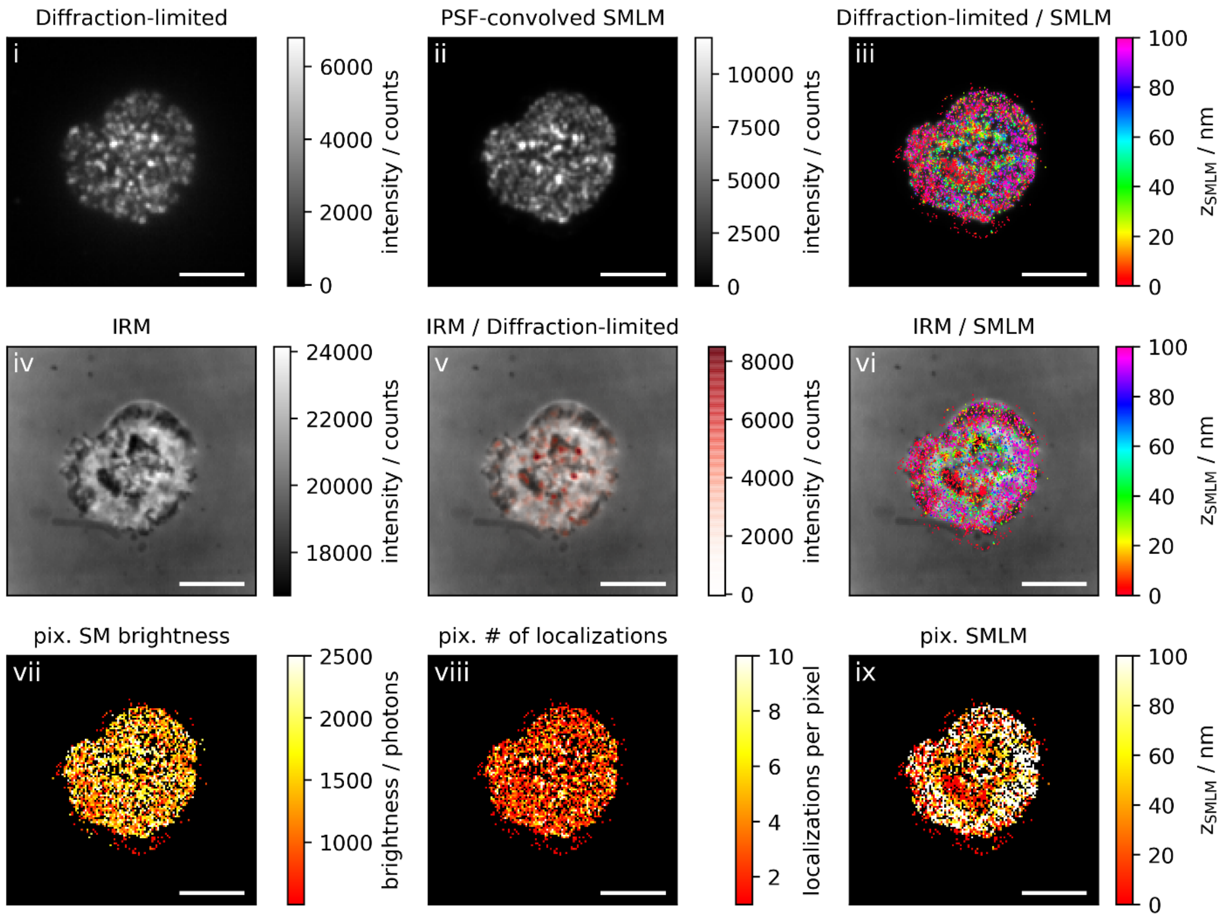

Diffraction-limited
i
intensity / counts
6000
4000
2000
0
PSF-convolved SMLM
ii
10000
7500
5000
2500
0
Diffraction-limited / SMLM
iii
z_{SMLM} / nm
100
80
60
40
20
0
IRM
iv
24000
22000
20000
18000
IRM / Diffraction-limited
v
8000
6000
4000
2000
0
IRM / SMLM
vi
pix. SM brightness
vii
brightness / photons
2500
2000
1500
1000
pix. # of localizations
viii
localizations per pixel
10
8
6
4
2
pix. SMLM
ix

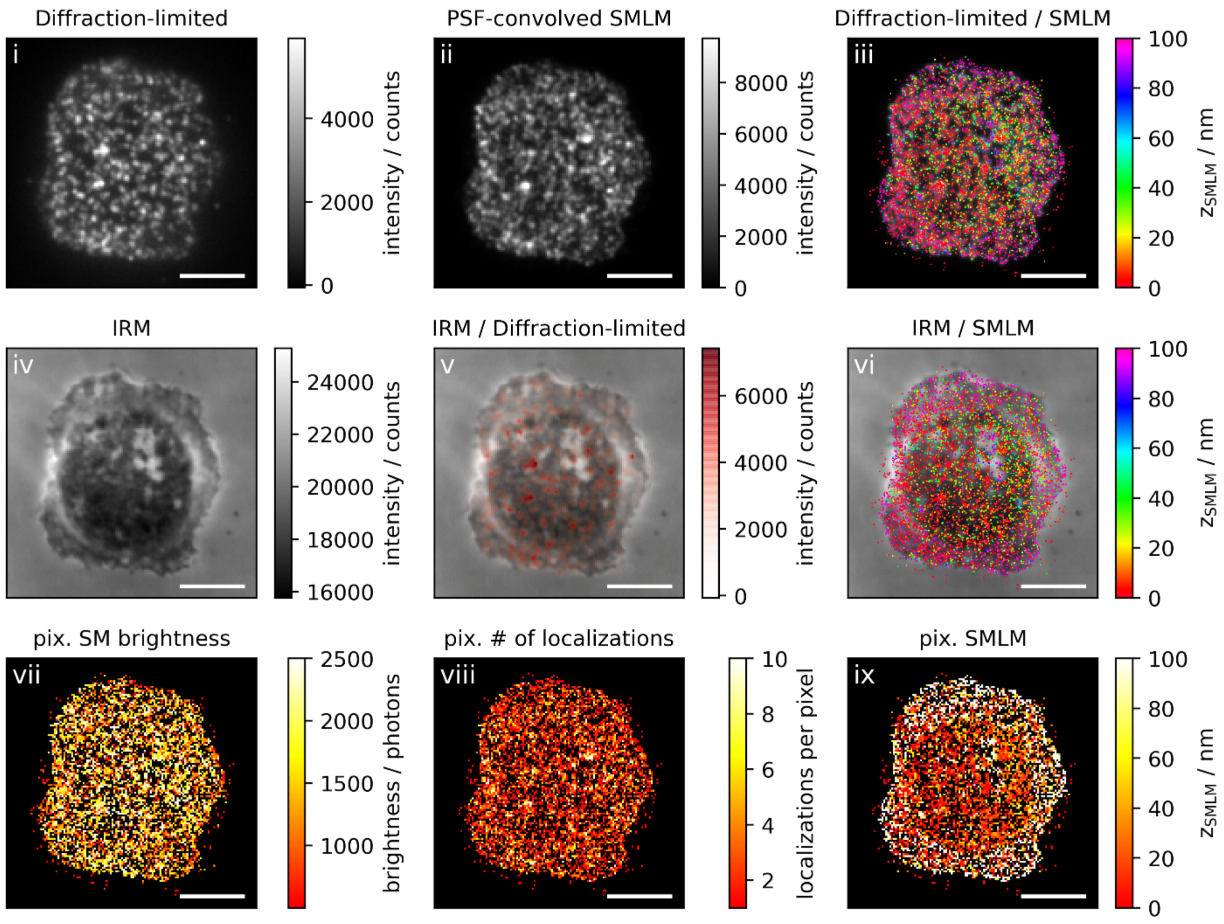

Diffraction-limited
i
4000
2000
0
intensity / counts
PSF-convolved SMLM
ii
8000
6000
4000
2000
0
intensity / counts
Diffraction-limited / SMLM
iii
100
80
60
40
20
0
z_{SMLM} / nm
IRM
iv
24000
22000
20000
18000
16000
intensity / counts
IRM / Diffraction-limited
v
6000
4000
2000
0
intensity / counts
IRM / SMLM
vi
100
80
60
40
20
0
z_{SMLM} / nm
pix. SM brightness
vii
2500
2000
1500
1000
brightness / photons
pix. # of localizations
viii
10
8
6
4
2
localizations per pixel
pix. SMLM
ix
100
80
60
40
20
0
z_{SMLM} / nm

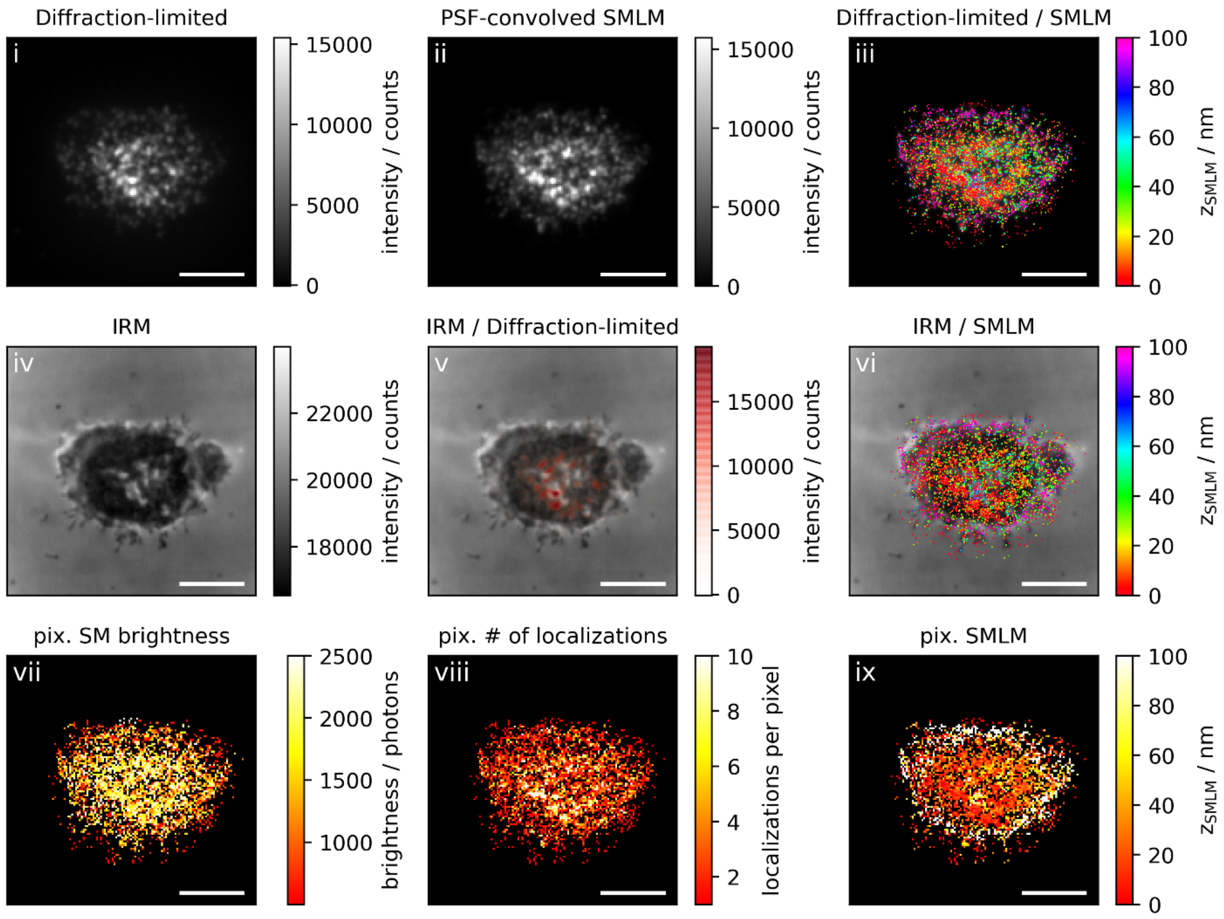

Diffraction-limited
i
15000
10000
5000
0
intensity / counts
PSF-convolved SMLM
ii
15000
10000
5000
0
intensity / counts
Diffraction-limited / SMLM
iii
100
80
60
40
20
0
z_{SMLM} / nm
IRM
iv
22000
20000
18000
intensity / counts
IRM / Diffraction-limited
v
15000
10000
5000
0
intensity / counts
IRM / SMLM
vi
100
80
60
40
20
0
z_{SMLM} / nm
pix. SM brightness
vii
2500
2000
1500
1000
brightness / photons
pix. # of localizations
viii
10
8
6
4
2
localizations per pixel
pix. SMLM
ix
100
80
60
40
20
0
z_{SMLM} / nm

Figure 1b: Activating conditions, high ICAM-1 density, fixation: 10 min post seeding

Correlative 3D-SMLM, IRM, and diffraction-limited TIR microscopy of the immunological synapse. T cells were activated on an SLB functionalized with I-E^k/MCC, B7-1 and high density of ICAM-1, and fixed 10 minutes post seeding. The T cell was imaged with IRM and fluorescence microscopy: (i) Diffraction-limited TIR image of the T cell. (ii) Reconstruction of the diffraction-limited image by convolving the 3D-SMLM image with the corresponding psf. (iii) Overlay of the diffraction-limited TIR image with the 3D-SMLM image. Color-code indicates distance to the coverslip z_{SMLM}. (iv) IRM image. (v) Overlay of the IRM image with the diffraction-limited image. (vi) Overlay of the IRM and the 3D-SMLM image. Bottom row images were generated by calculating the pixel-wise average of the 3D-SMLM images (pixel size of 146nm is consistent with diffraction-limited image) according to pixelated mean single molecule (SM) intensity (vii), pixelated number of localizations (viii) and pixelated mean z_{SMLM} (ix). Scale bars 5 µm.

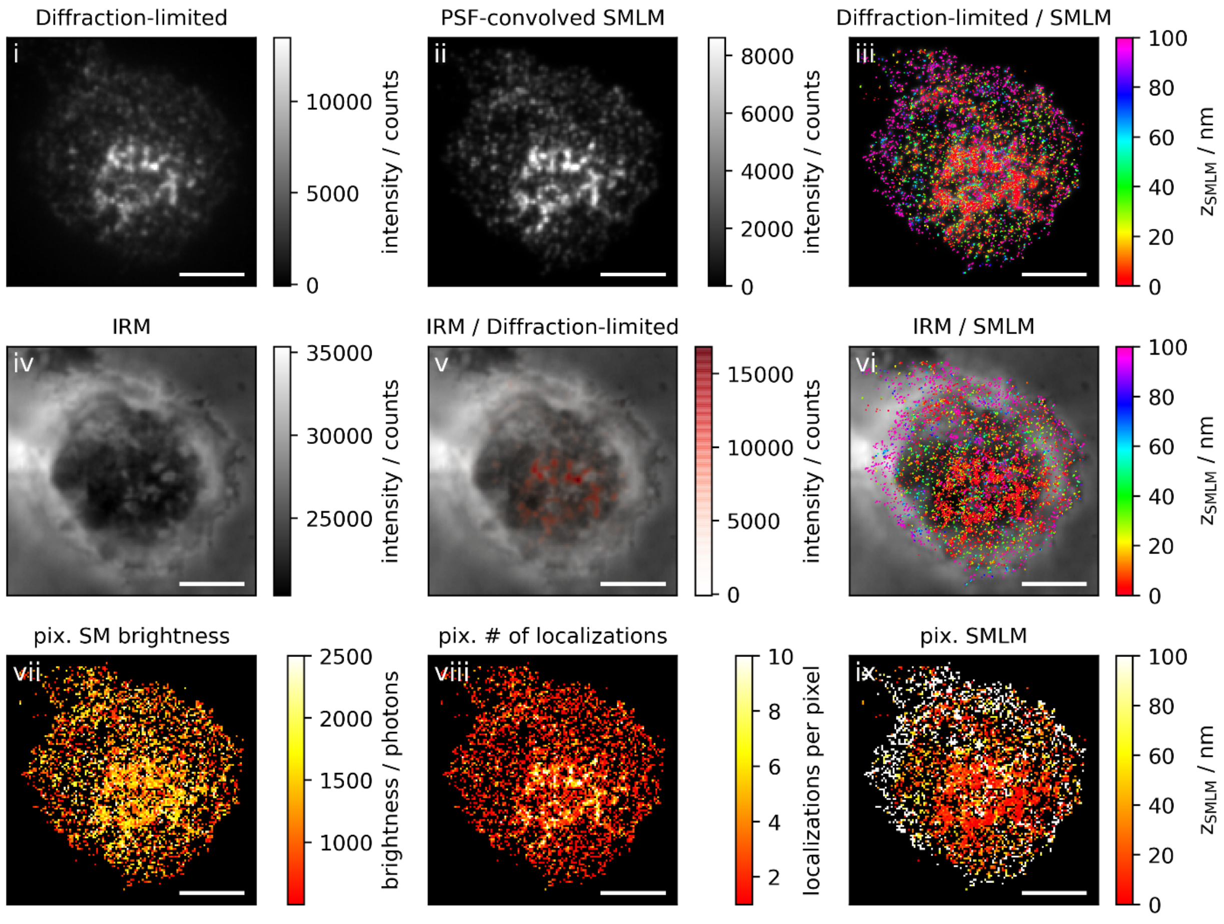
Diffraction-limited
i
10000
5000
0
intensity / counts
PSF-convolved SMLM
ii
8000
6000
4000
2000
0
intensity / counts
Diffraction-limited / SMLM
iii
100
80
60
40
20
0
z_{SMLM} / nm
IRM
iv
35000
30000
25000
intensity / counts
IRM / Diffraction-limited
v
15000
10000
5000
0
intensity / counts
IRM / SMLM
vi
100
80
60
40
20
0
z_{SMLM} / nm
pix. SM brightness
vii
2500
2000
1500
1000
brightness / photons
pix. # of localizations
viii
10
8
6
4
2
localizations per pixel
pix. SMLM
ix
100
80
60
40
20
0
z_{SMLM} / nm

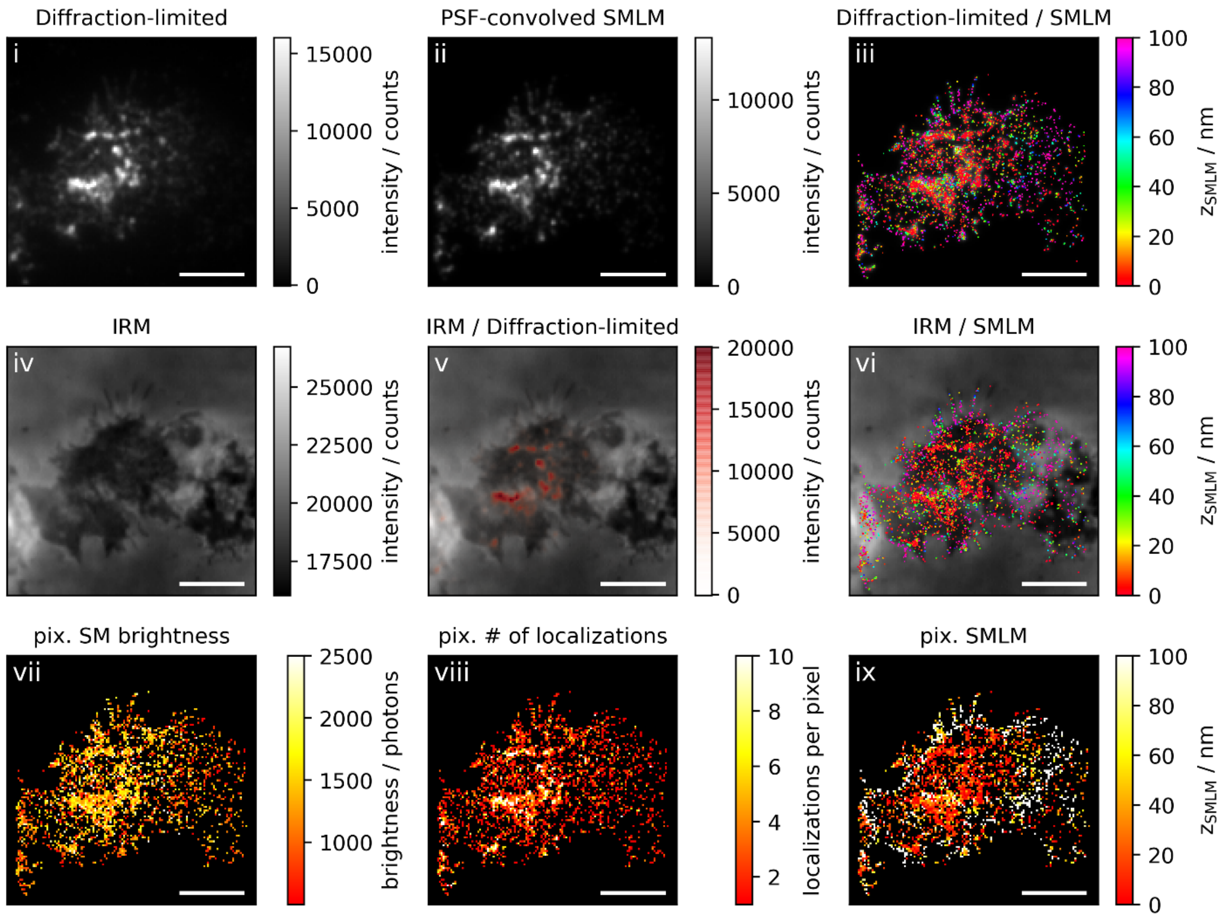
Diffraction-limited
i
15000
10000
5000
0
intensity / counts
PSF-convolved SMLM
ii
10000
5000
0
intensity / counts
Diffraction-limited / SMLM
iii
100
80
60
40
20
0
z_{SMLM} / nm
IRM
iv
25000
22500
20000
17500
intensity / counts
IRM / Diffraction-limited
v
20000
15000
10000
5000
0
intensity / counts
IRM / SMLM
vi
100
80
60
40
20
0
z_{SMLM} / nm
pix. SM brightness
vii
2500
2000
1500
1000
brightness / photons
pix. # of localizations
viii
10
8
6
4
2
localizations per pixel
pix. SMLM
ix
100
80
60
40
20
0
z_{SMLM} / nm

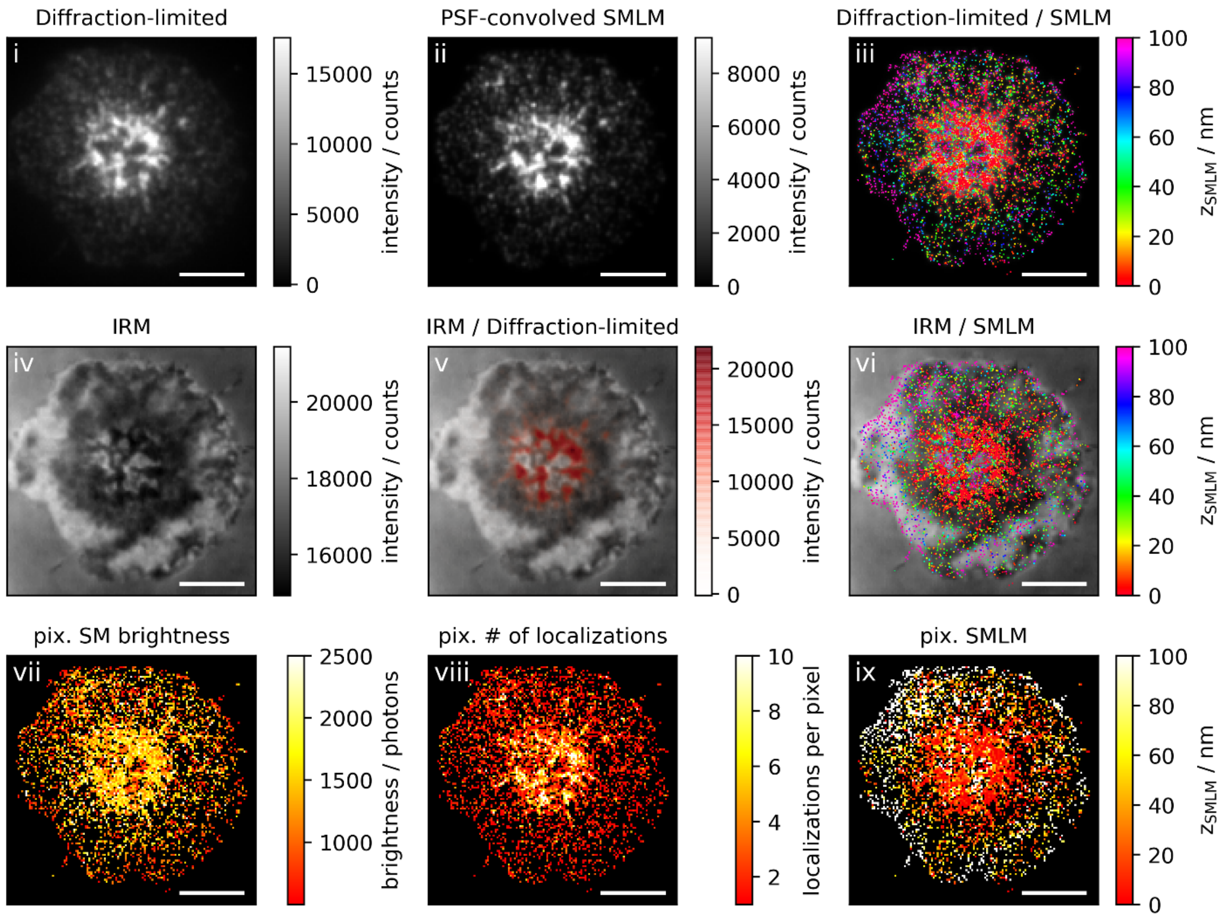

Diffraction-limited
i
15000
10000
5000
0
intensity / counts
PSF-convolved SMLM
ii
8000
6000
4000
2000
0
intensity / counts
Diffraction-limited / SMLM
iii
100
80
60
40
20
0
z_{SMLM} / nm
IRM
iv
20000
18000
16000
intensity / counts
IRM / Diffraction-limited
v
20000
15000
10000
5000
0
intensity / counts
IRM / SMLM
vi
100
80
60
40
20
0
z_{SMLM} / nm
pix. SM brightness
vii
2500
2000
1500
1000
brightness / photons
pix. # of localizations
viii
10
8
6
4
2
localizations per pixel
pix. SMLM
ix
100
80
60
40
20
0
z_{SMLM} / nm

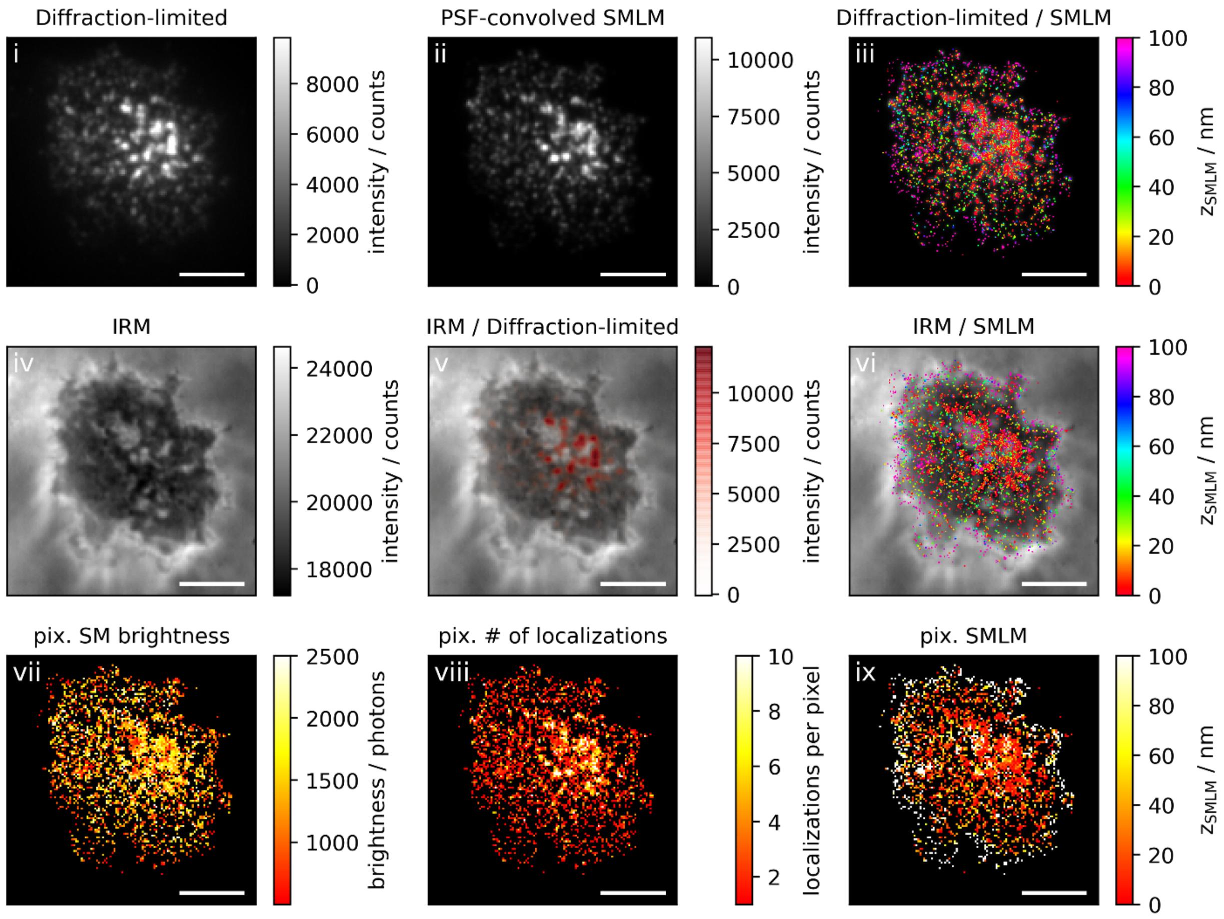

Diffraction-limited
PSF-convolved SMLM
Diffraction-limited / SMLM
i
ii
iii
intensity / counts
intensity / counts
z_{SMLM} / nm
0
2000
4000
6000
8000
0
2500
5000
7500
10000
0
20
40
60
80
100
IRM
IRM / Diffraction-limited
IRM / SMLM
iv
v
vi
intensity / counts
intensity / counts
z_{SMLM} / nm
18000
20000
22000
24000
0
2500
5000
7500
10000
0
20
40
60
80
100
pix. SM brightness
pix. # of localizations
pix. SMLM
vii
viii
ix
brightness / photons
localizations per pixel
z_{SMLM} / nm
1000
1500
2000
2500
2
4
6
8
10
0
20
40
60
80
100

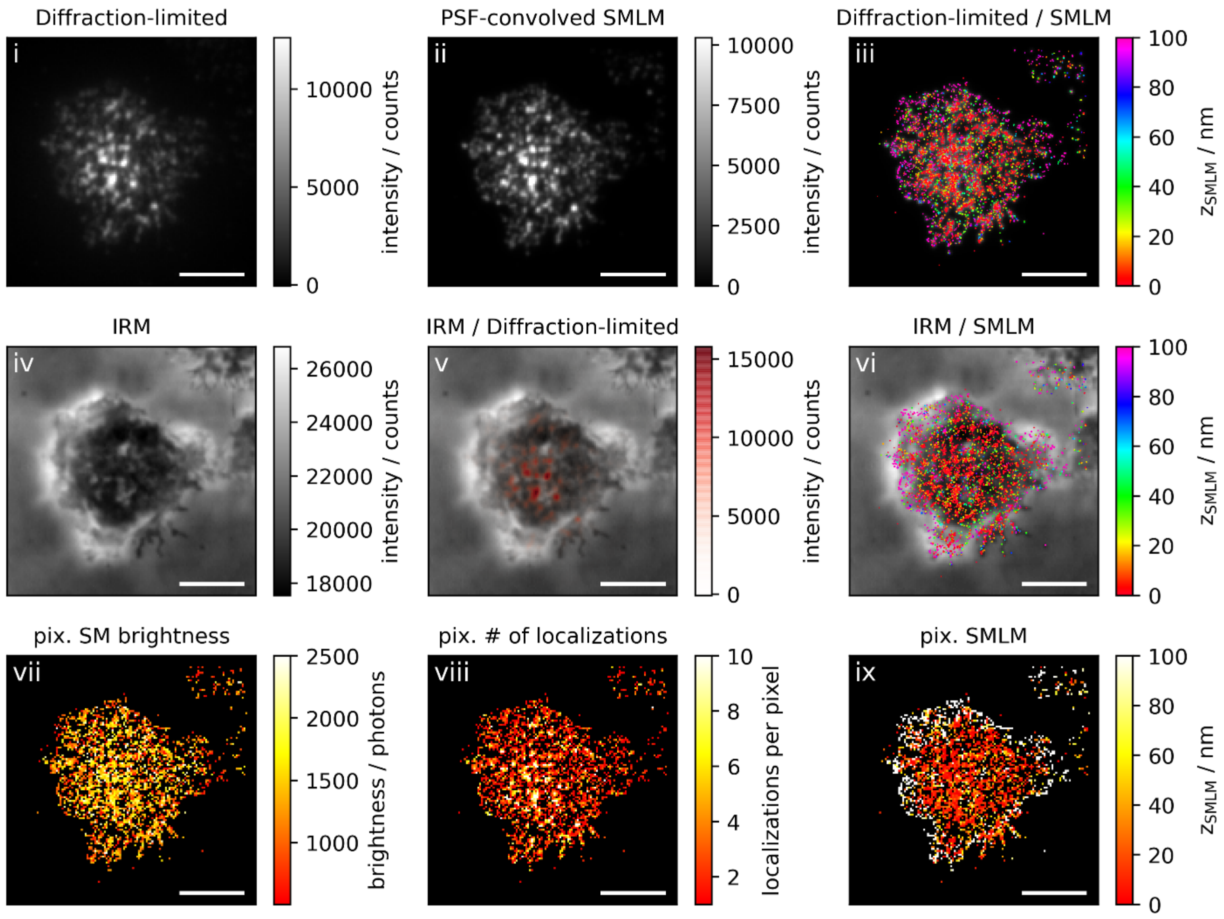
Diffraction-limited
i
10000
5000
0
intensity / counts
PSF-convolved SMLM
ii
10000
7500
5000
2500
0
intensity / counts
Diffraction-limited / SMLM
iii
100
80
60
40
20
0
z_{SMLM} / nm
IRM
iv
26000
24000
22000
20000
18000
intensity / counts
IRM / Diffraction-limited
v
15000
10000
5000
0
intensity / counts
IRM / SMLM
vi
100
80
60
40
20
0
z_{SMLM} / nm
pix. SM brightness
vii
2500
2000
1500
1000
brightness / photons
pix. # of localizations
viii
10
8
6
4
2
localizations per pixel
pix. SMLM
ix
100
80
60
40
20
0
z_{SMLM} / nm

Figure 1c: Activating conditions, high ICAM-1 density, fixation: 10-15 min post seeding

Correlative 3D-SMLM, IRM, and diffraction-limited TIR microscopy of the immunological synapse. T cells were activated on an SLB functionalized with I-E^k/MCC, B7-1 and high density of ICAM-1, and fixed 10-15 minutes post seeding. The T cell was imaged with IRM and fluorescence microscopy: (i) Diffraction-limited TIR image of the T cell. (ii) Reconstruction of the diffraction-limited image by convolving the 3D-SMLM image with the corresponding psf. (iii) Overlay of the diffraction-limited TIR image with the 3D-SMLM image. Color-code indicates distance to the coverslip z_{SMLM}. (iv) IRM image. (v) Overlay of the IRM image with the diffraction-limited image. (vi) Overlay of the IRM and the 3D-SMLM image. Bottom row images were generated by calculating the pixel-wise average of the 3D-SMLM images (pixel size of 146nm is consistent with diffraction-limited image) according to pixelated mean single molecule (SM) intensity (vii), pixelated number of localizations (viii) and pixelated mean z_{SMLM} (ix). Scale bars 5 µm.

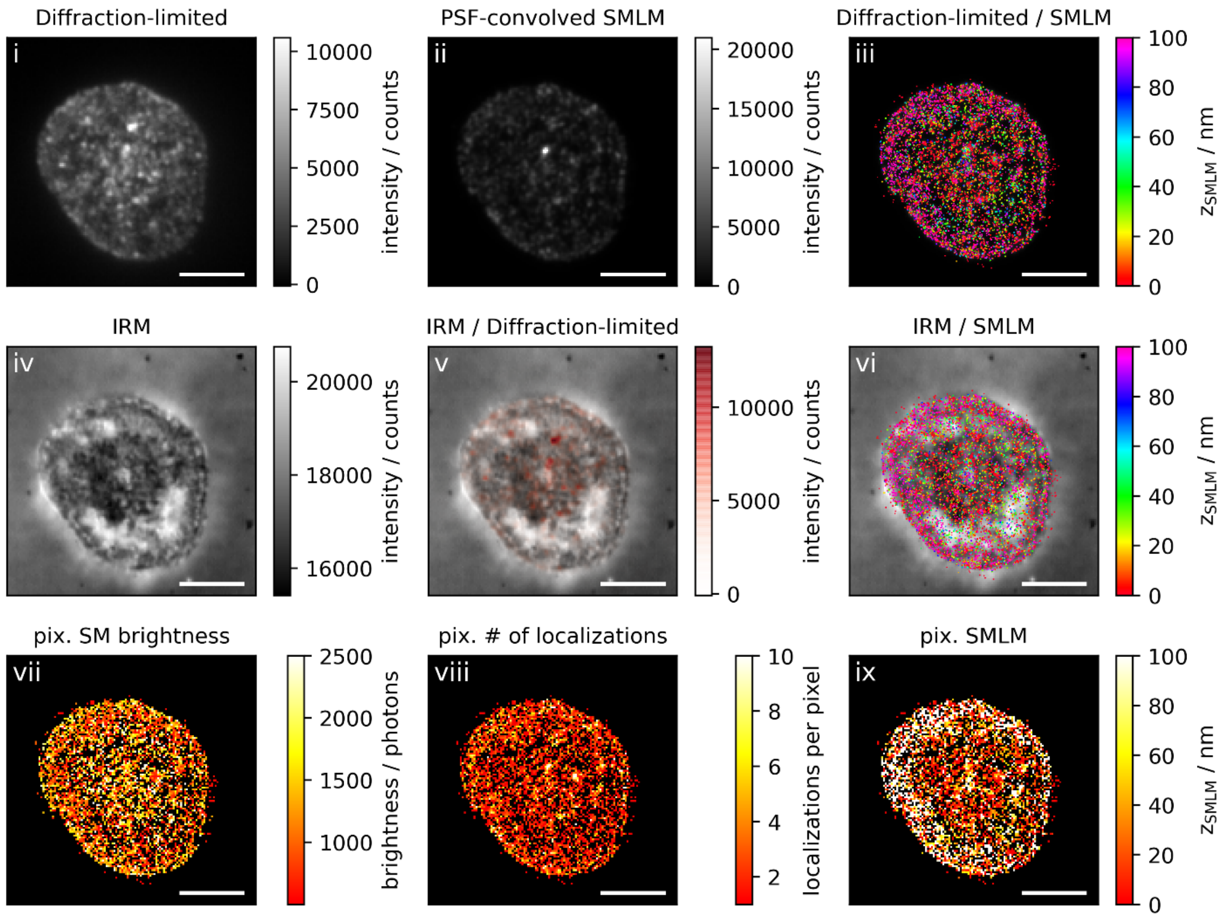

Diffraction-limited
i
10000
7500
5000
2500
0
intensity / counts
PSF-convolved SMLM
ii
20000
15000
10000
5000
0
intensity / counts
Diffraction-limited / SMLM
iii
100
80
60
40
20
0
zSMLM / nm
IRM
iv
20000
18000
16000
intensity / counts
IRM / Diffraction-limited
v
10000
5000
0
intensity / counts
IRM / SMLM
vi
100
80
60
40
20
0
zSMLM / nm
pix. SM brightness
vii
2500
2000
1500
1000
brightness / photons
pix. # of localizations
viii
10
8
6
4
2
localizations per pixel
pix. SMLM
ix
100
80
60
40
20
0
zSMLM / nm

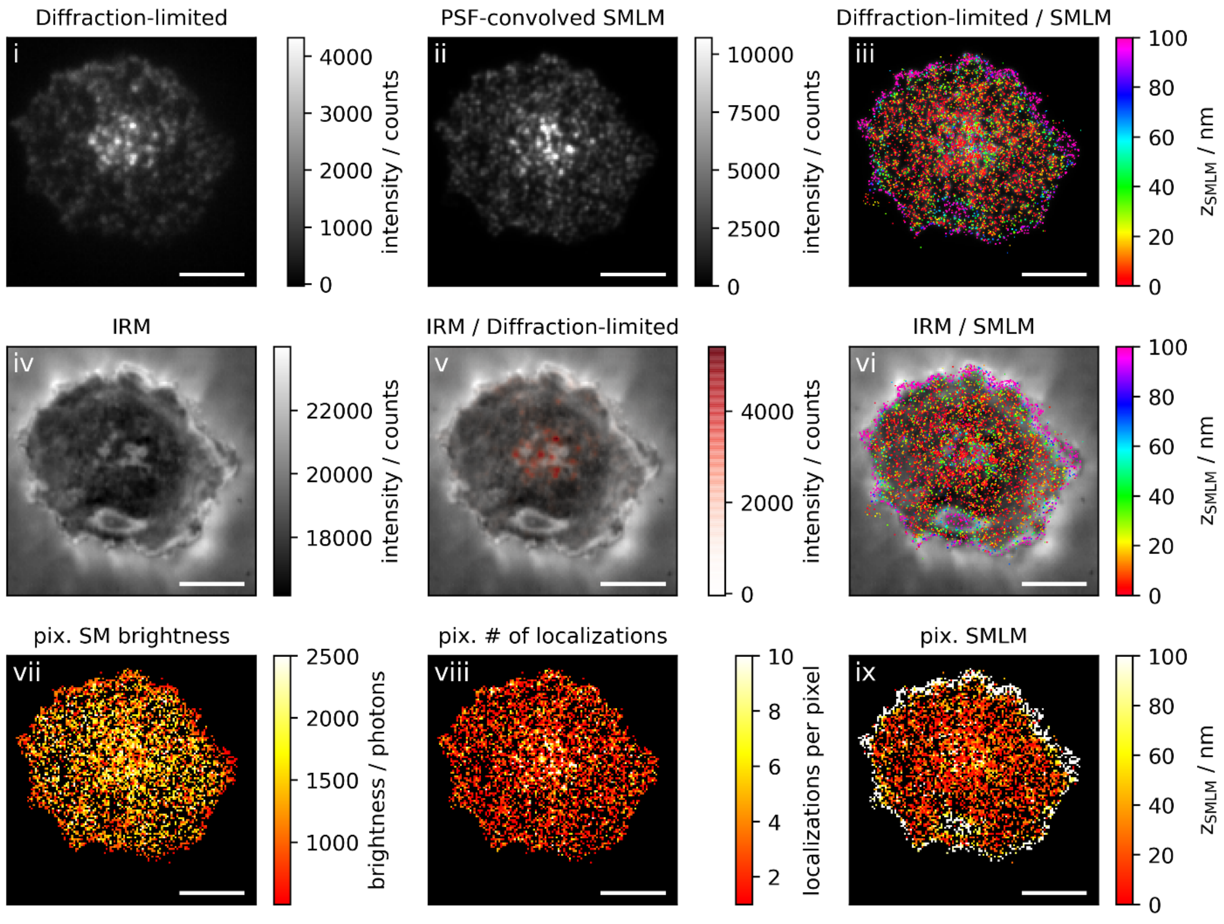
Diffraction-limited
i
4000
3000
2000
1000
0
intensity / counts
PSF-convolved SMLM
ii
10000
7500
5000
2500
0
intensity / counts
Diffraction-limited / SMLM
iii
100
80
60
40
20
0
zSMLM / nm
IRM
iv
22000
20000
18000
intensity / counts
IRM / Diffraction-limited
v
4000
2000
0
intensity / counts
IRM / SMLM
vi
100
80
60
40
20
0
zSMLM / nm
pix. SM brightness
vii
2500
2000
1500
1000
brightness / photons
pix. # of localizations
viii
10
8
6
4
2
localizations per pixel
pix. SMLM
ix
100
80
60
40
20
0
zSMLM / nm

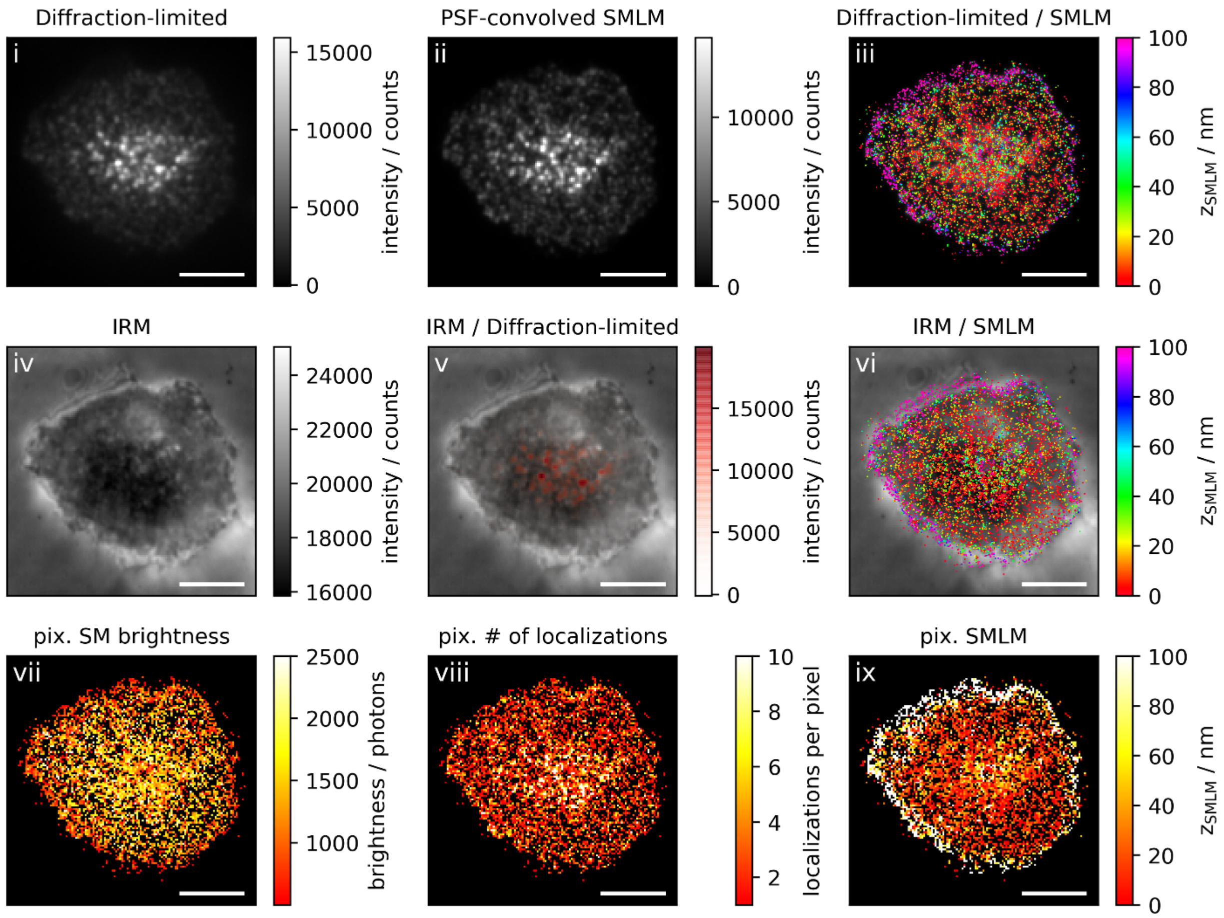

Diffraction-limited
i
15000
10000
5000
0
intensity / counts
PSF-convolved SMLM
ii
10000
5000
0
intensity / counts
Diffraction-limited / SMLM
iii
100
80
60
40
20
0
z_{SMLM} / nm
IRM
iv
24000
22000
20000
18000
16000
intensity / counts
IRM / Diffraction-limited
v
15000
10000
5000
0
intensity / counts
IRM / SMLM
vi
100
80
60
40
20
0
z_{SMLM} / nm
pix. SM brightness
vii
2500
2000
1500
1000
brightness / photons
pix. # of localizations
viii
10
8
6
4
2
localizations per pixel
pix. SMLM
ix
100
80
60
40
20
0
z_{SMLM} / nm

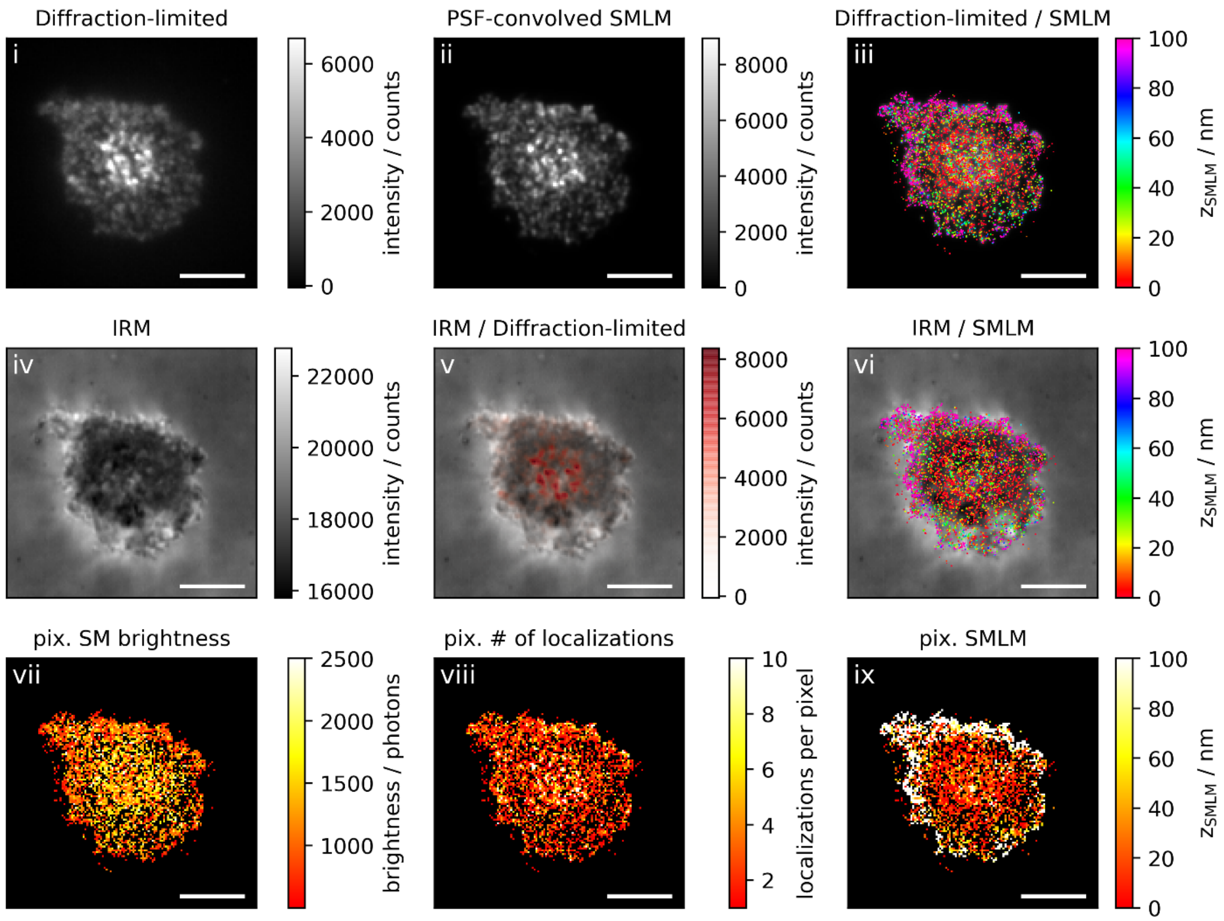
Diffraction-limited
i
intensity / counts
PSF-convolved SMLM
ii
intensity / counts
Diffraction-limited / SMLM
iii
z_{SMLM} / nm
IRM
iv
intensity / counts
IRM / Diffraction-limited
v
intensity / counts
IRM / SMLM
vi
z_{SMLM} / nm
pix. SM brightness
vii
brightness / photons
pix. # of localizations
viii
localizations per pixel
pix. SMLM
ix
z_{SMLM} / nm

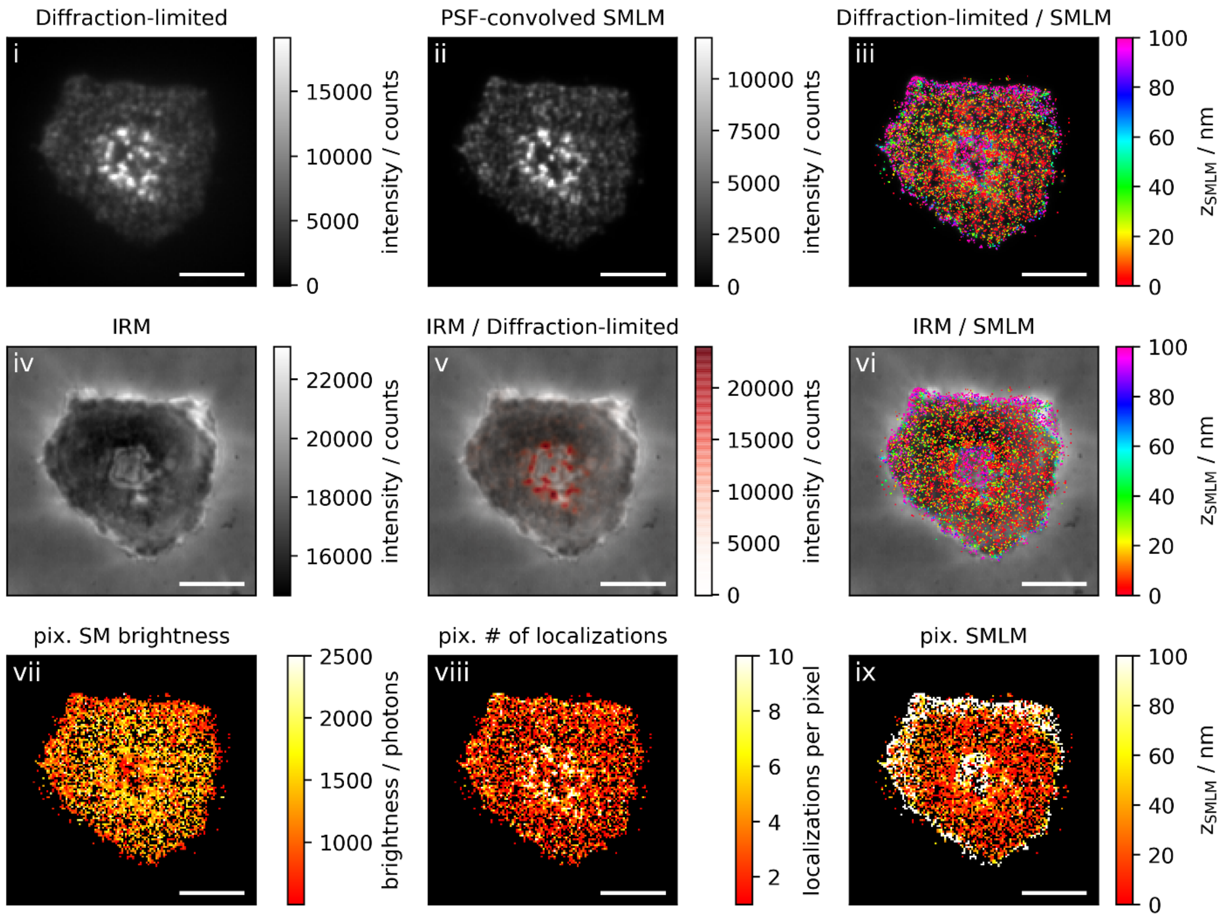

Diffraction-limited
i
15000
10000
5000
0
intensity / counts
PSF-convolved SMLM
ii
10000
7500
5000
2500
0
intensity / counts
Diffraction-limited / SMLM
iii
100
80
60
40
20
0
z_{SMLM} / nm
IRM
iv
22000
20000
18000
16000
intensity / counts
IRM / Diffraction-limited
v
20000
15000
10000
5000
0
intensity / counts
IRM / SMLM
vi
100
80
60
40
20
0
z_{SMLM} / nm
pix. SM brightness
vii
2500
2000
1500
1000
brightness / photons
pix. # of localizations
viii
10
8
6
4
2
localizations per pixel
pix. SMLM
ix
100
80
60
40
20
0
z_{SMLM} / nm

Figure 2a: Activating conditions, low ICAM-1 density, fixation: 5-10 min post seeding

Correlative 3D-SMLM, IRM, and diffraction-limited TIR microscopy of the immunological synapse. T cells were activated on an SLB functionalized with I-E^k/MCC, B7-1 and low density of ICAM-1, and fixed 5-10 minutes post seeding. The T cell was imaged with IRM and fluorescence microscopy: (i) Diffraction-limited TIR image of the T cell. (ii) Reconstruction of the diffraction-limited image by convolving the 3D-SMLM image with the corresponding psf. (iii) Overlay of the diffraction-limited TIR image with the 3D-SMLM image. Color-code indicates distance to the coverslip z_{SMLM}. (iv) IRM image. (v) Overlay of the IRM image with the diffraction-limited image. (vi) Overlay of the IRM and the 3D-SMLM image. Bottom row images were generated by calculating the pixel-wise average of the 3D-SMLM images (pixel size of 146nm is consistent with diffraction-limited image) according to pixelated mean single molecule (SM) intensity (vii), pixelated number of localizations (viii) and pixelated mean z_{SMLM} (ix). Scale bars 5 µm.

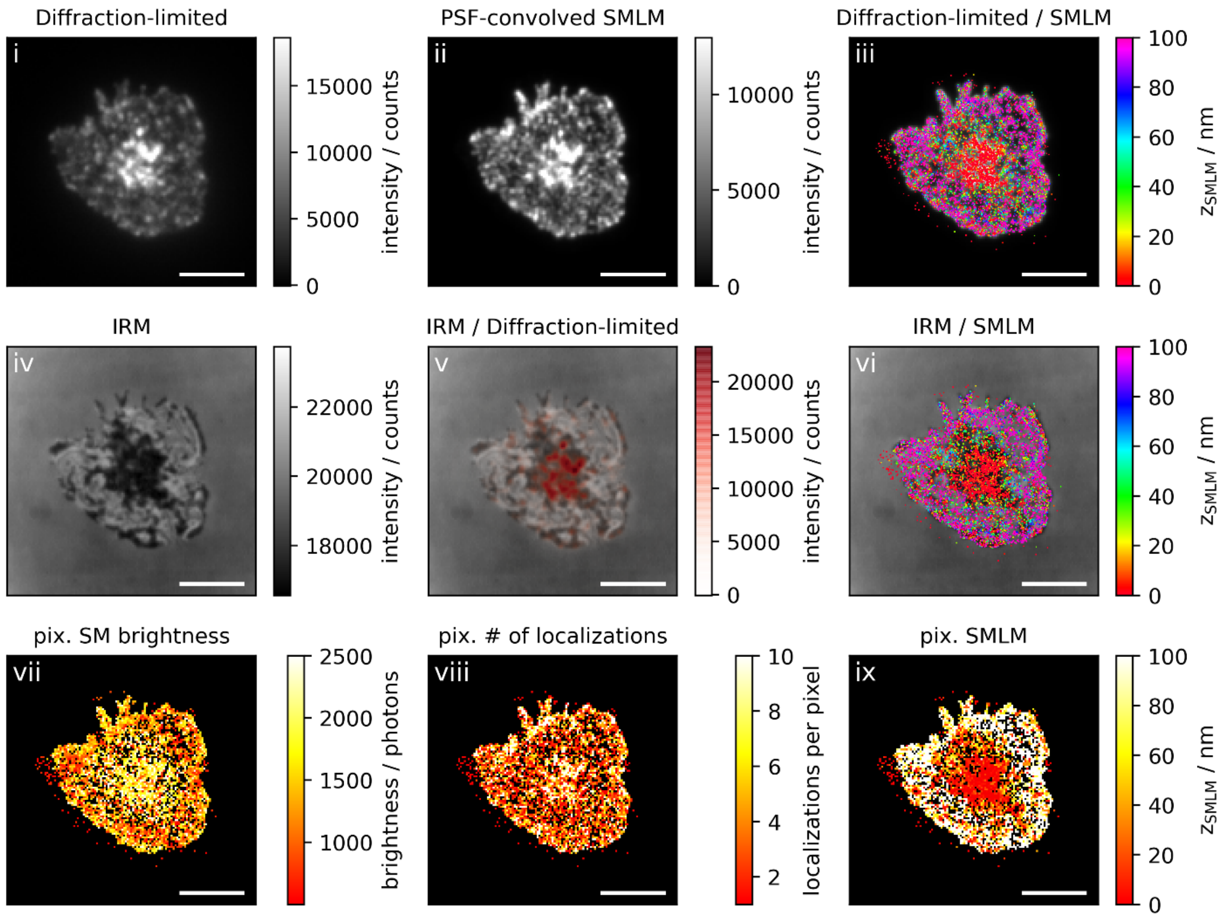

Diffraction-limited
i
15000
10000
5000
0
intensity / counts
PSF-convolved SMLM
ii
10000
5000
0
intensity / counts
Diffraction-limited / SMLM
iii
100
80
60
40
20
0
z_SMLM / nm
IRM
iv
22000
20000
18000
intensity / counts
IRM / Diffraction-limited
v
20000
15000
10000
5000
0
intensity / counts
IRM / SMLM
vi
100
80
60
40
20
0
z_SMLM / nm
pix. SM brightness
vii
2500
2000
1500
1000
brightness / photons
pix. # of localizations
viii
10
8
6
4
2
localizations per pixel
pix. SMLM
ix
100
80
60
40
20
0
z_SMLM / nm

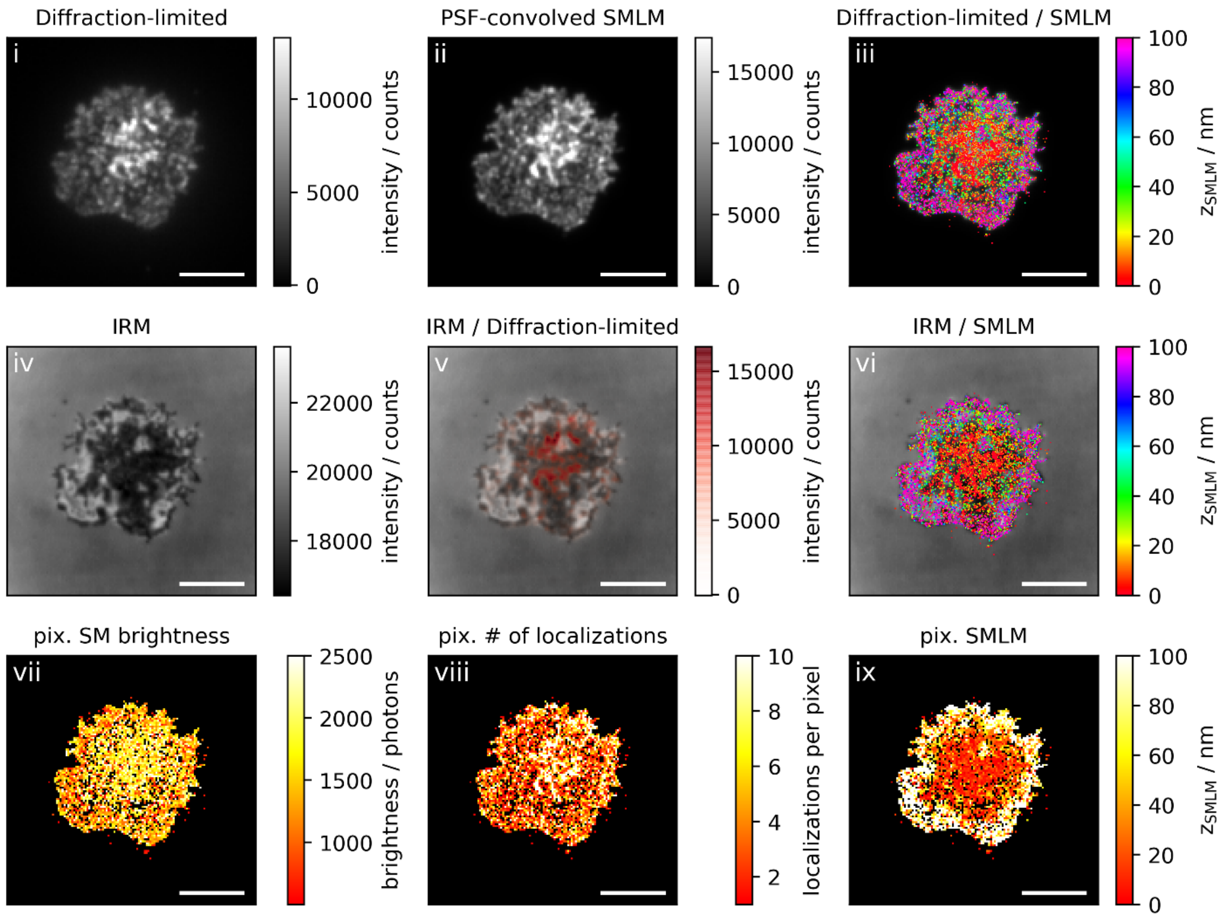
Diffraction-limited
i
10000
5000
0
intensity / counts
PSF-convolved SMLM
ii
15000
10000
5000
0
intensity / counts
Diffraction-limited / SMLM
iii
100
80
60
40
20
0
z_{SMLM} / nm
IRM
iv
22000
20000
18000
intensity / counts
IRM / Diffraction-limited
v
15000
10000
5000
0
intensity / counts
IRM / SMLM
vi
100
80
60
40
20
0
z_{SMLM} / nm
pix. SM brightness
vii
2500
2000
1500
1000
brightness / photons
pix. # of localizations
viii
10
8
6
4
2
localizations per pixel
pix. SMLM
ix
100
80
60
40
20
0
z_{SMLM} / nm

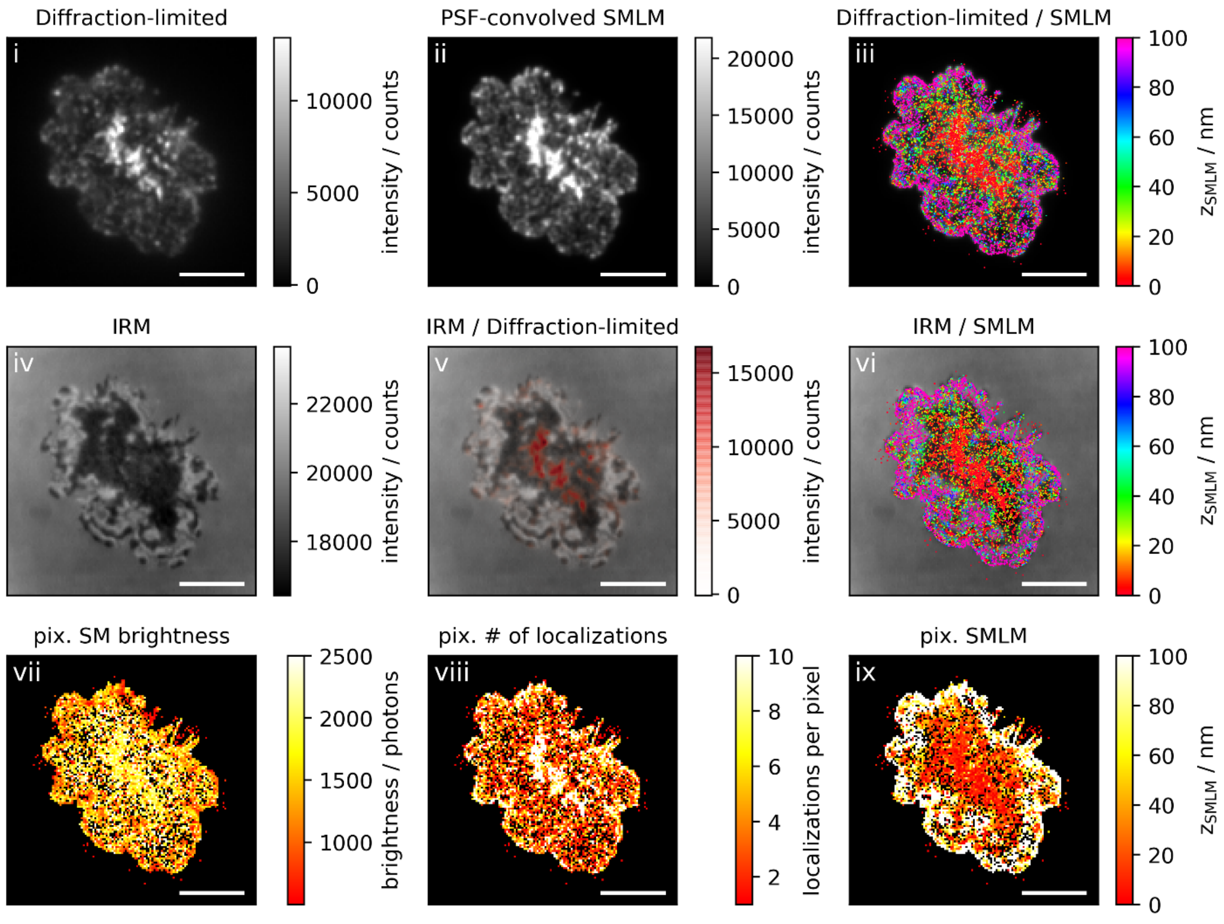

Diffraction-limited
i
intensity / counts
PSF-convolved SMLM
ii
intensity / counts
Diffraction-limited / SMLM
iii
z_SMLM / nm
IRM
iv
intensity / counts
IRM / Diffraction-limited
v
intensity / counts
IRM / SMLM
vi
z_SMLM / nm
pix. SM brightness
vii
brightness / photons
pix. # of localizations
viii
localizations per pixel
pix. SMLM
ix
z_SMLM / nm

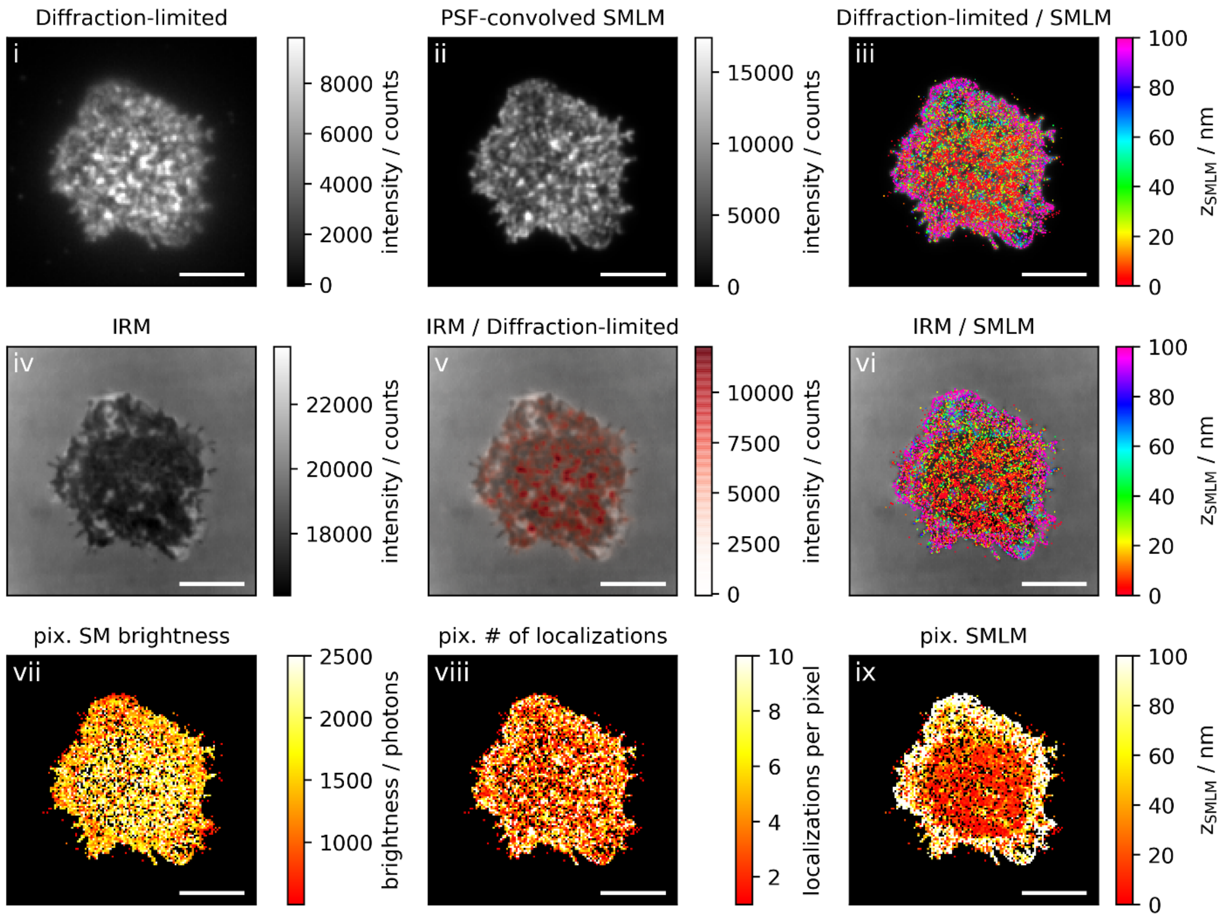

Diffraction-limited
i
8000
6000
4000
2000
0
intensity / counts
PSF-convolved SMLM
ii
15000
10000
5000
0
intensity / counts
Diffraction-limited / SMLM
iii
100
80
60
40
20
0
z_{SMLM} / nm
IRM
iv
22000
20000
18000
intensity / counts
IRM / Diffraction-limited
v
10000
7500
5000
2500
0
intensity / counts
IRM / SMLM
vi
100
80
60
40
20
0
z_{SMLM} / nm
pix. SM brightness
vii
2500
2000
1500
1000
brightness / photons
pix. # of localizations
viii
10
8
6
4
2
localizations per pixel
pix. SMLM
ix
100
80
60
40
20
0
z_{SMLM} / nm

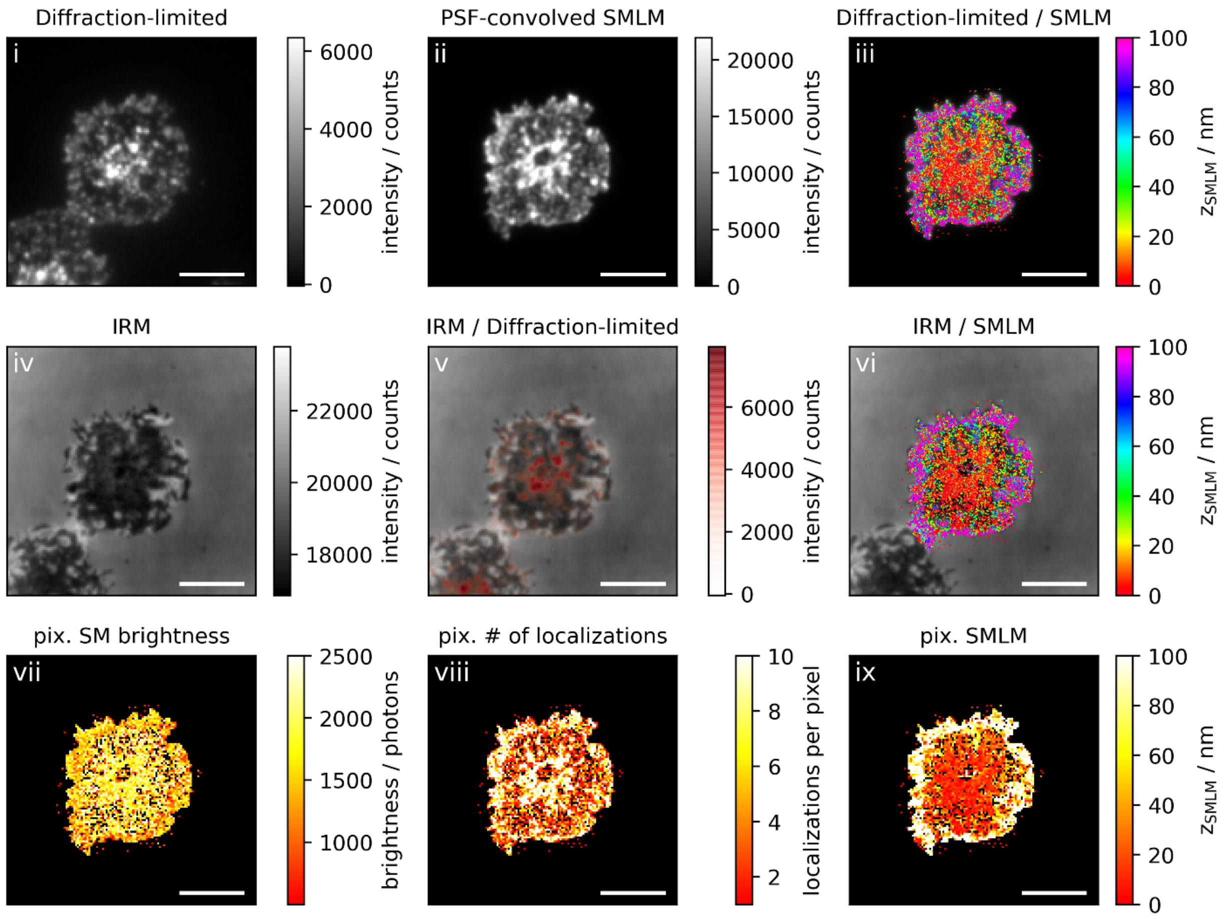
Diffraction-limited
i
6000
4000
2000
0
intensity / counts
PSF-convolved SMLM
ii
20000
15000
10000
5000
0
intensity / counts
Diffraction-limited / SMLM
iii
100
80
60
40
20
0
z_{SMLM} / nm
IRM
iv
22000
20000
18000
intensity / counts
IRM / Diffraction-limited
v
6000
4000
2000
0
intensity / counts
IRM / SMLM
vi
100
80
60
40
20
0
z_{SMLM} / nm
pix. SM brightness
vii
2500
2000
1500
1000
brightness / photons
pix. # of localizations
viii
10
8
6
4
2
localizations per pixel
pix. SMLM
ix
100
80
60
40
20
0
z_{SMLM} / nm

Figure 2b: Activating conditions, low ICAM-1 density, fixation: 10 min post seeding

Correlative 3D-SMLM, IRM, and diffraction-limited TIR microscopy of the immunological synapse. T cells were activated on an SLB functionalized with I-E^k/MCC, B7-1 and low density of ICAM-1, and fixed 10 minutes post seeding. The T cell was imaged with IRM and fluorescence microscopy: (i) Diffraction-limited TIR image of the T cell. (ii) Reconstruction of the diffraction-limited image by convolving the 3D-SMLM image with the corresponding psf. (iii) Overlay of the diffraction-limited TIR image with the 3D-SMLM image. Color-code indicates distance to the coverslip z_{SMLM}. (iv) IRM image. (v) Overlay of the IRM image with the diffraction-limited image. (vi) Overlay of the IRM and the 3D-SMLM image. Bottom row images were generated by calculating the pixel-wise average of the 3D-SMLM images (pixel size of 146nm is consistent with diffraction-limited image) according to pixelated mean single molecule (SM) intensity (vii), pixelated number of localizations (viii) and pixelated mean z_{SMLM} (ix). Scale bars 5 µm.

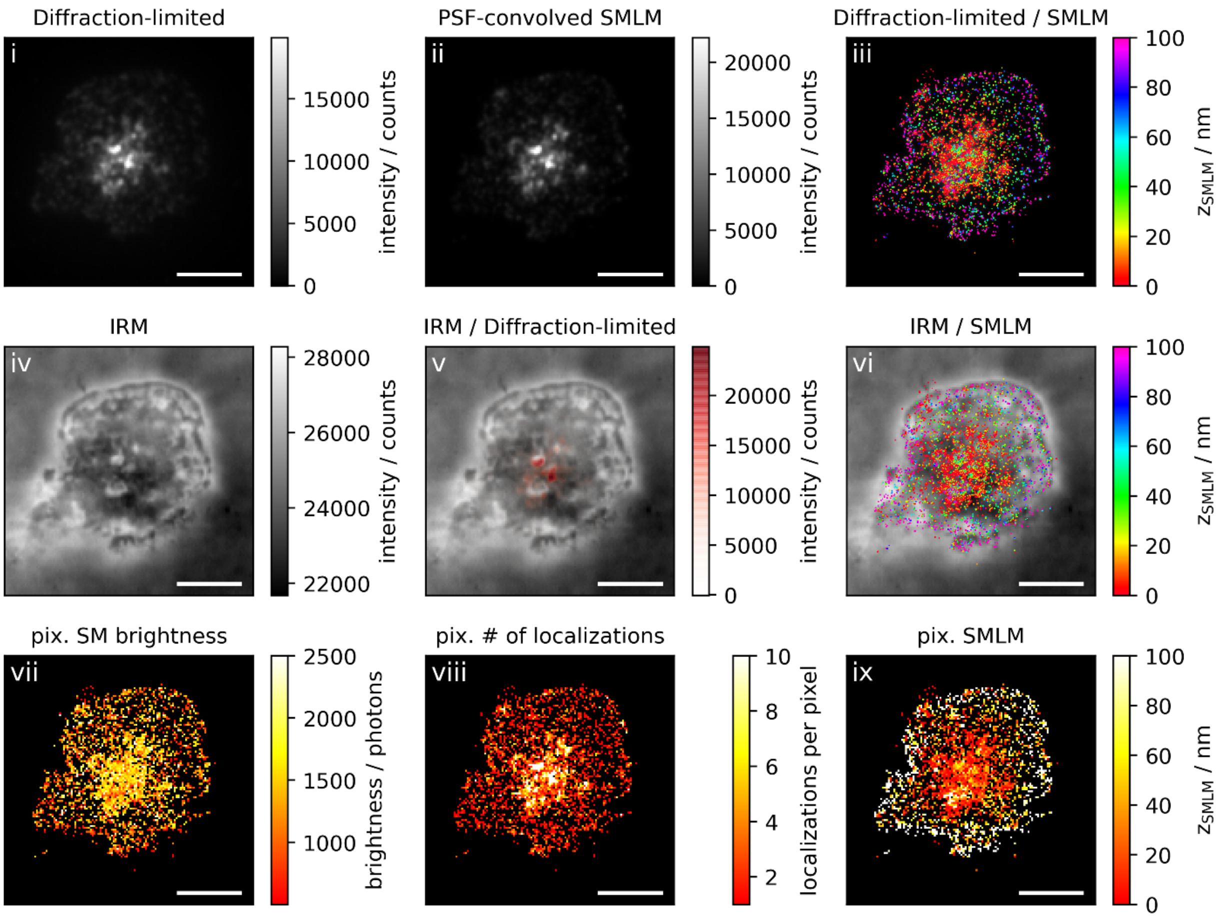
Diffraction-limited
i
intensity / counts
PSF-convolved SMLM
ii
Diffraction-limited / SMLM
iii
z_{SMLM} / nm
IRM
iv
IRM / Diffraction-limited
v
IRM / SMLM
vi
pix. SM brightness
vii
brightness / photons
pix. # of localizations
viii
localizations per pixel
pix. SMLM
ix

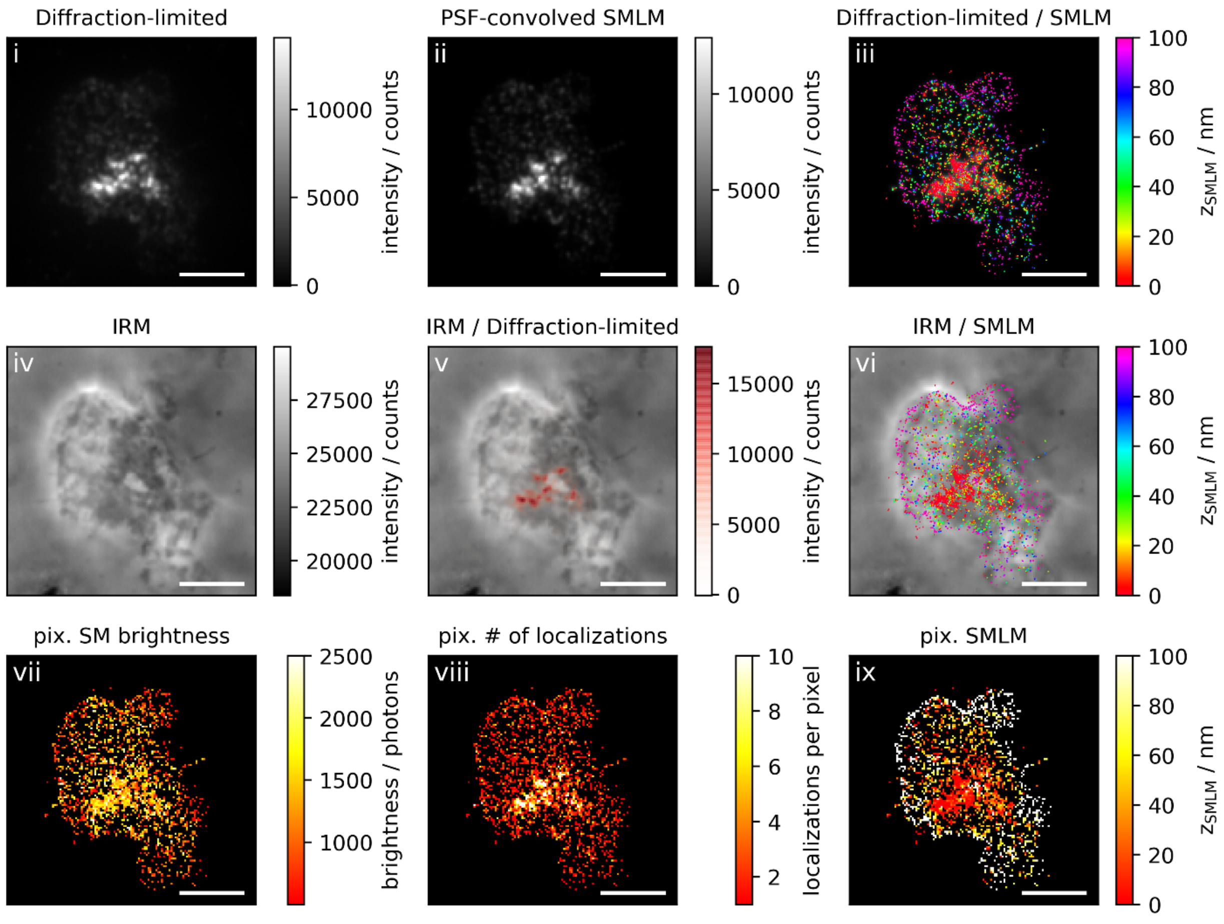
Diffraction-limited
PSF-convolved SMLM
Diffraction-limited / SMLM
i
ii
iii
10000
5000
0
intensity / counts
100
80
60
40
20
zSMLM / nm
IRM
IRM / Diffraction-limited
IRM / SMLM
iv
v
vi
27500
25000
22500
20000
15000
pix. SM brightness
pix. # of localizations
pix. SMLM
vii
viii
ix
2500
2000
1500
1000
brightness / photons
10
8
6
4
2
localizations per pixel

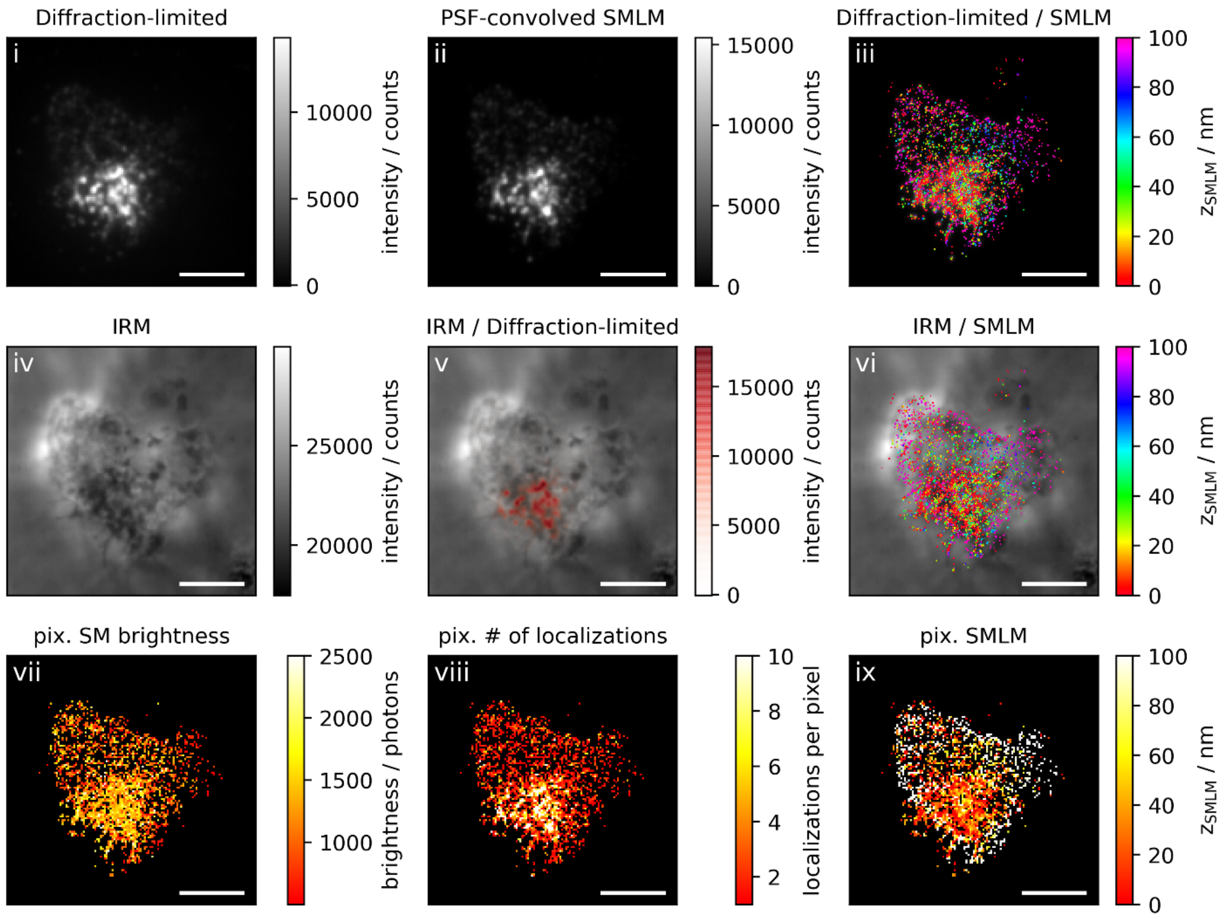

Diffraction-limited
i
10000
5000
0
intensity / counts
PSF-convolved SMLM
ii
15000
10000
5000
0
intensity / counts
Diffraction-limited / SMLM
iii
100
80
60
40
20
0
z_{SMLM} / nm
IRM
iv
25000
20000
intensity / counts
IRM / Diffraction-limited
v
15000
10000
5000
0
intensity / counts
IRM / SMLM
vi
100
80
60
40
20
0
z_{SMLM} / nm
pix. SM brightness
vii
2500
2000
1500
1000
brightness / photons
pix. # of localizations
viii
10
8
6
4
2
localizations per pixel
pix. SMLM
ix
100
80
60
40
20
0
z_{SMLM} / nm

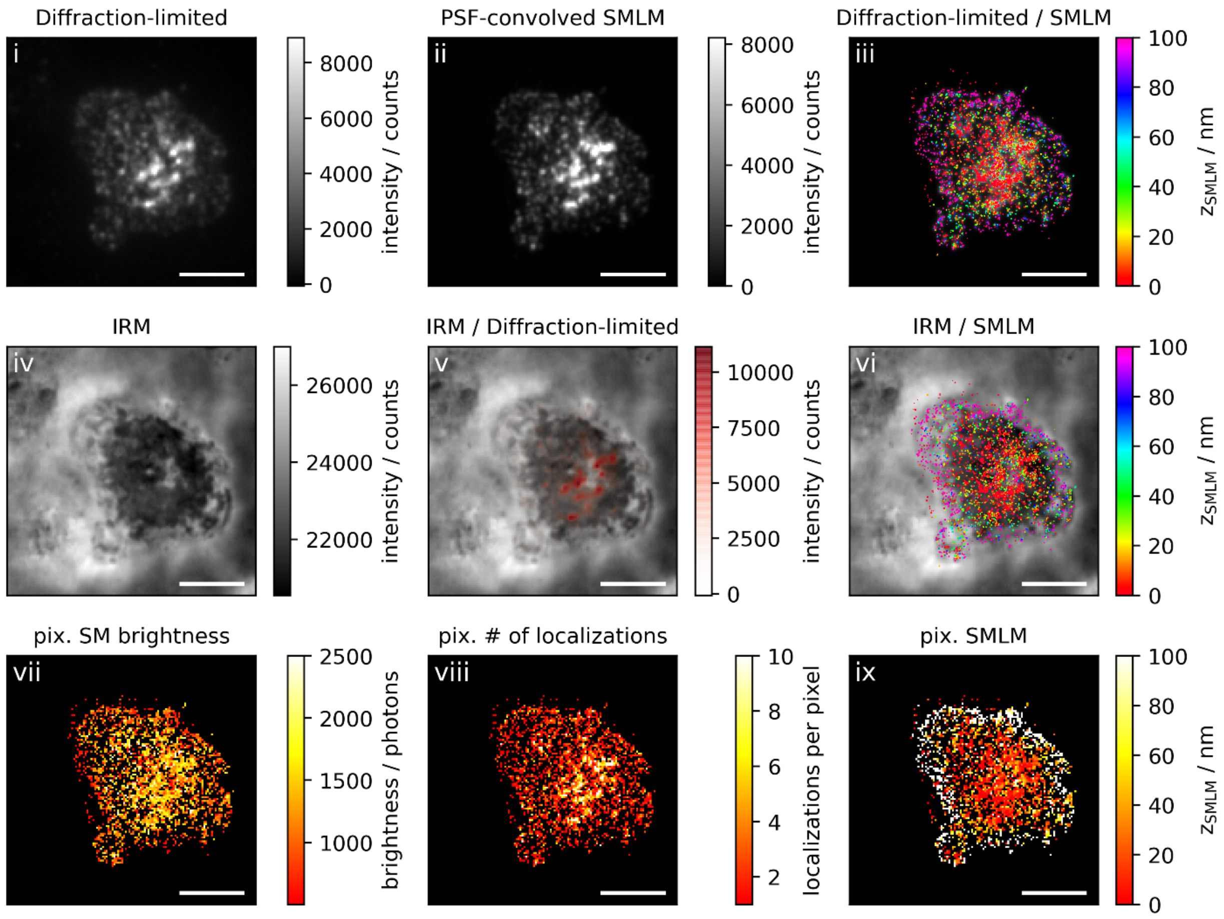

Diffraction-limited
i
8000
6000
4000
2000
0
intensity / counts
PSF-convolved SMLM
ii
8000
6000
4000
2000
0
intensity / counts
Diffraction-limited / SMLM
iii
100
80
60
40
20
0
z_{SMLM} / nm
IRM
iv
26000
24000
22000
intensity / counts
IRM / Diffraction-limited
v
10000
7500
5000
2500
0
intensity / counts
IRM / SMLM
vi
100
80
60
40
20
0
z_{SMLM} / nm
pix. SM brightness
vii
2500
2000
1500
1000
brightness / photons
pix. # of localizations
viii
10
8
6
4
2
localizations per pixel
pix. SMLM
ix
100
80
60
40
20
0
z_{SMLM} / nm

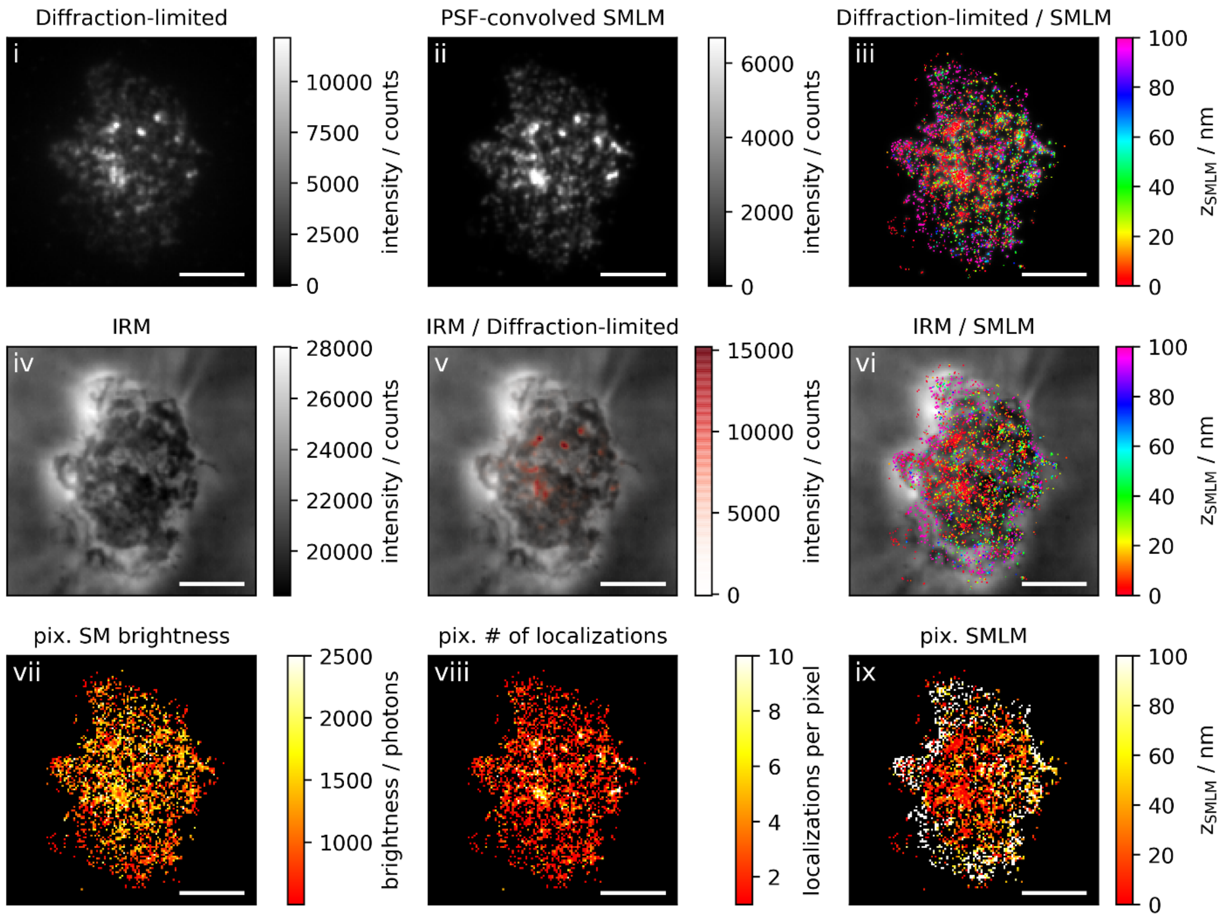
Diffraction-limited
i
intensity / counts
0
2500
5000
7500
10000
PSF-convolved SMLM
ii
intensity / counts
0
2000
4000
6000
Diffraction-limited / SMLM
iii
z_{SMLM} / nm
0
20
40
60
80
100
IRM
iv
intensity / counts
20000
22000
24000
26000
28000
IRM / Diffraction-limited
v
intensity / counts
0
5000
10000
15000
IRM / SMLM
vi
z_{SMLM} / nm
0
20
40
60
80
100
pix. SM brightness
vii
brightness / photons
1000
1500
2000
2500
pix. # of localizations
viii
localizations per pixel
2
4
6
8
10
pix. SMLM
ix
z_{SMLM} / nm
0
20
40
60
80
100

Figure 2c: Activating conditions, low ICAM-1 density, fixation: 10-15 min post seeding

Correlative 3D-SMLM, IRM, and diffraction-limited TIR microscopy of the immunological synapse. T cells were activated on an SLB functionalized with I-E^k/MCC, B7-1 and low density of ICAM-1, and fixed 10-15 minutes post seeding. The T cell was imaged with IRM and fluorescence microscopy: (i) Diffraction-limited TIR image of the T cell. (ii) Reconstruction of the diffraction-limited image by convolving the 3D-SMLM image with the corresponding psf. (iii) Overlay of the diffraction-limited TIR image with the 3D-SMLM image. Color-code indicates distance to the coverslip z_{SMLM}. (iv) IRM image. (v) Overlay of the IRM image with the diffraction-limited image. (vi) Overlay of the IRM and the 3D-SMLM image. Bottom row images were generated by calculating the pixel-wise average of the 3D-SMLM images (pixel size of 146nm is consistent with diffraction-limited image) according to pixelated mean single molecule (SM) intensity (vii), pixelated number of localizations (viii) and pixelated mean z_{SMLM} (ix). Scale bars 5 µm.

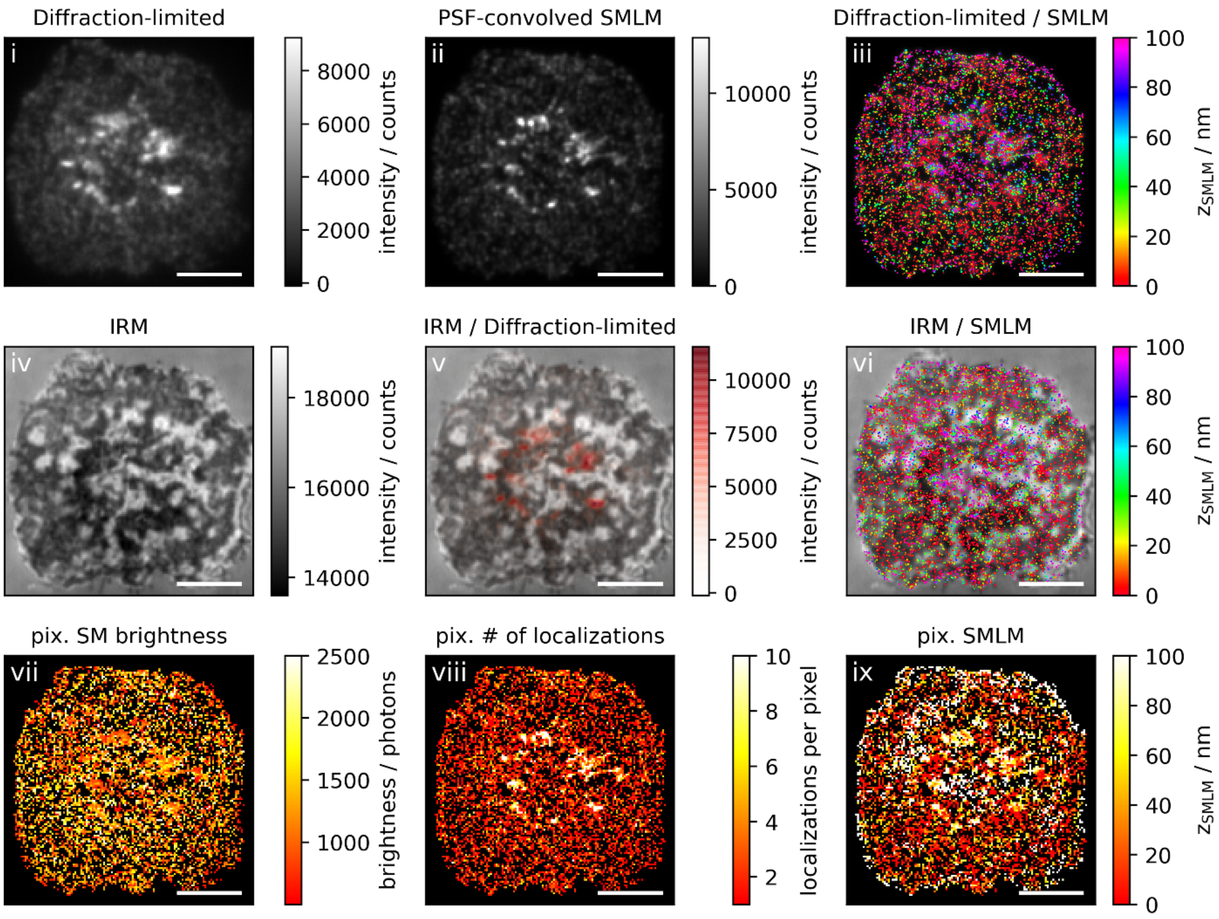

Diffraction-limited
PSF-convolved SMLM
Diffraction-limited / SMLM
i
ii
iii
8000
6000
4000
2000
0
intensity / counts
10000
5000
0
100
80
60
40
20
0
z_{SMLM} / nm
IRM
IRM / Diffraction-limited
IRM / SMLM
iv
v
vi
18000
16000
14000
10000
7500
5000
2500
pix. SM brightness
pix. # of localizations
pix. SMLM
vii
viii
ix
2500
2000
1500
1000
brightness / photons
10
8
6
4
2
localizations per pixel

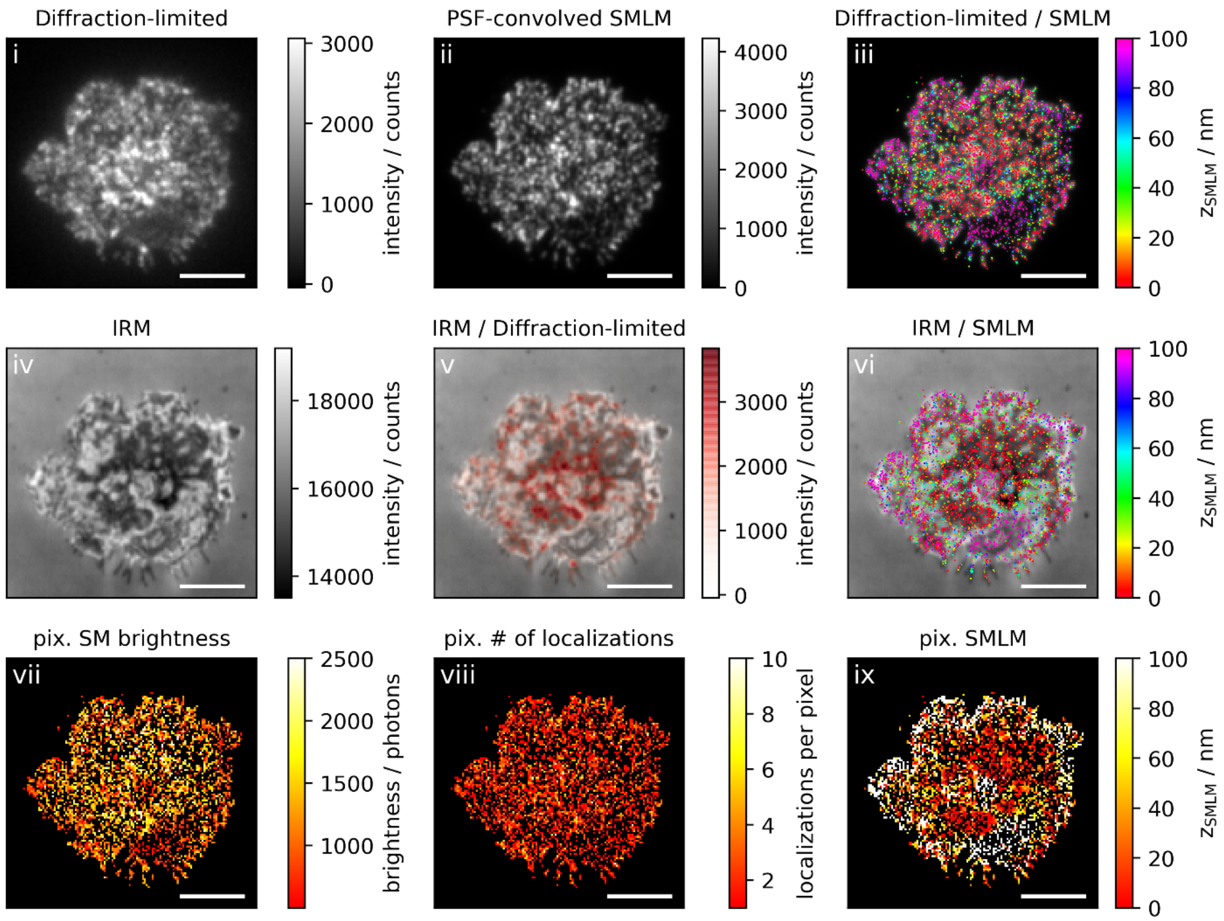

Diffraction-limited
i
3000
2000
1000
0
intensity / counts
PSF-convolved SMLM
ii
4000
3000
2000
1000
0
intensity / counts
Diffraction-limited / SMLM
iii
100
80
60
40
20
0
z_{SMLM} / nm
IRM
iv
18000
16000
14000
intensity / counts
IRM / Diffraction-limited
v
3000
2000
1000
0
intensity / counts
IRM / SMLM
vi
100
80
60
40
20
0
z_{SMLM} / nm
pix. SM brightness
vii
2500
2000
1500
1000
brightness / photons
pix. # of localizations
viii
10
8
6
4
2
localizations per pixel
pix. SMLM
ix
100
80
60
40
20
0
z_{SMLM} / nm

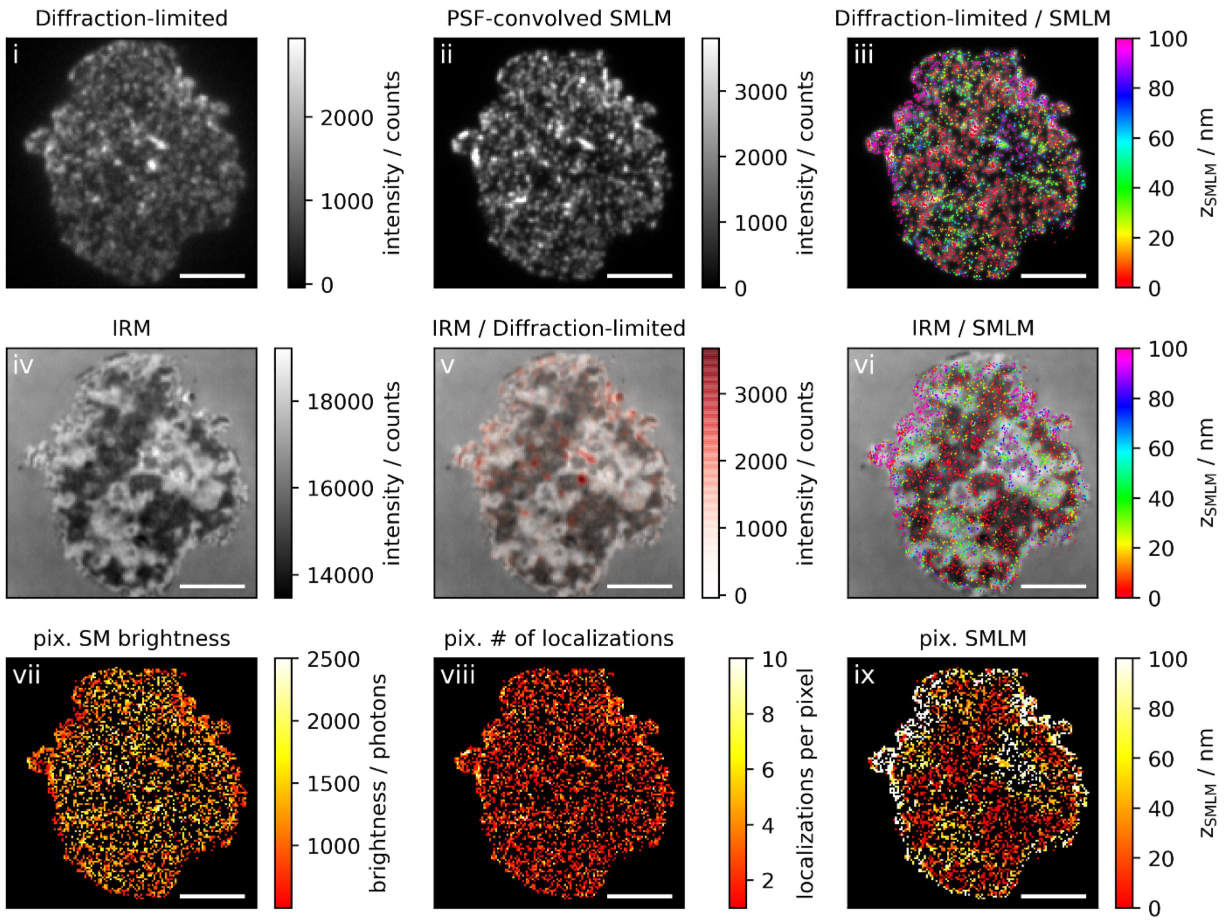
Diffraction-limited
i
intensity / counts
PSF-convolved SMLM
ii
intensity / counts
Diffraction-limited / SMLM
iii
z_{SMLM} / nm
IRM
iv
intensity / counts
IRM / Diffraction-limited
v
intensity / counts
IRM / SMLM
vi
z_{SMLM} / nm
pix. SM brightness
vii
brightness / photons
pix. # of localizations
viii
localizations per pixel
pix. SMLM
ix
z_{SMLM} / nm

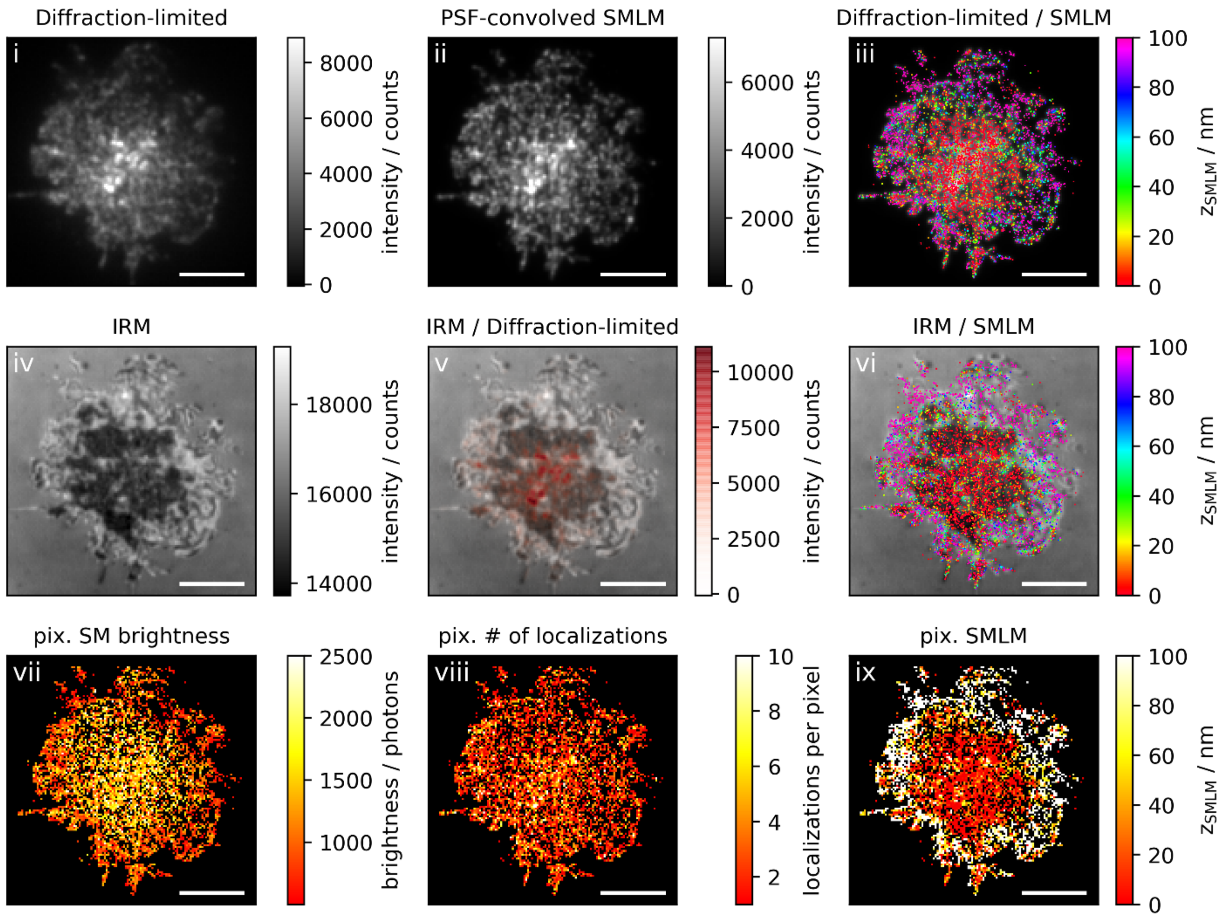

Diffraction-limited
i
8000
6000
4000
2000
0
intensity / counts
PSF-convolved SMLM
ii
6000
4000
2000
0
intensity / counts
Diffraction-limited / SMLM
iii
100
80
60
40
20
0
z_{SMLM} / nm
IRM
iv
18000
16000
14000
intensity / counts
IRM / Diffraction-limited
v
10000
7500
5000
2500
0
intensity / counts
IRM / SMLM
vi
100
80
60
40
20
0
z_{SMLM} / nm
pix. SM brightness
vii
2500
2000
1500
1000
brightness / photons
pix. # of localizations
viii
10
8
6
4
2
localizations per pixel
pix. SMLM
ix
100
80
60
40
20
0
z_{SMLM} / nm

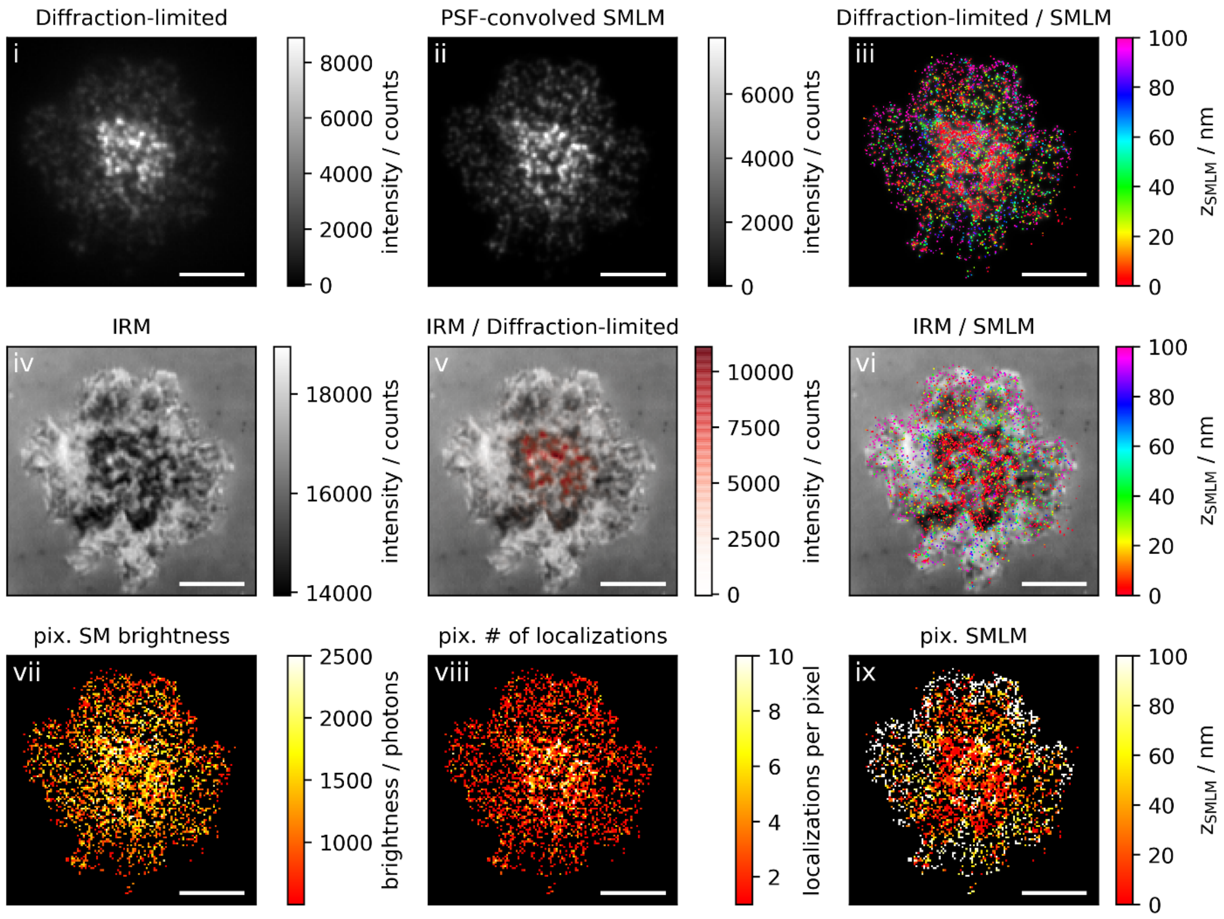
Diffraction-limited
i
8000
6000
4000
2000
0
intensity / counts
PSF-convolved SMLM
ii
6000
4000
2000
0
intensity / counts
Diffraction-limited / SMLM
iii
100
80
60
40
20
0
zSMLM / nm
IRM
iv
18000
16000
14000
intensity / counts
IRM / Diffraction-limited
v
10000
7500
5000
2500
0
intensity / counts
IRM / SMLM
vi
100
80
60
40
20
0
zSMLM / nm
pix. SM brightness
vii
2500
2000
1500
1000
brightness / photons
pix. # of localizations
viii
10
8
6
4
2
localizations per pixel
pix. SMLM
ix
100
80
60
40
20
0
zSMLM / nm

Figure 3a: Non-activating conditions, high ICAM-1 density, fixation: 5-10 min post seeding

Correlative 3D-SMLM, IRM, and diffraction-limited TIR microscopy of the immunological synapse. T cells were seeded on an SLB functionalized with high density of ICAM-1, and fixed 5-10 minutes post seeding. The T cell was imaged with IRM and fluorescence microscopy: (i) Diffraction-limited TIR image of the T cell. (ii) Reconstruction of the diffraction-limited image by convolving the 3D-SMLM image with the corresponding psf. (iii) Overlay of the diffraction-limited TIR image with the 3D-SMLM image. Color-code indicates distance to the coverslip z_{SMLM}. (iv) IRM image. (v) Overlay of the IRM image with the diffraction-limited image. (vi) Overlay of the IRM and the 3D-SMLM image. Bottom row images were generated by calculating the pixel-wise average of the 3D-SMLM images (pixel size of 146nm is consistent with diffraction-limited image) according to pixelated mean single molecule (SM) intensity (vii), pixelated number of localizations (viii) and pixelated mean z_{SMLM} (ix). Scale bars 5 µm.

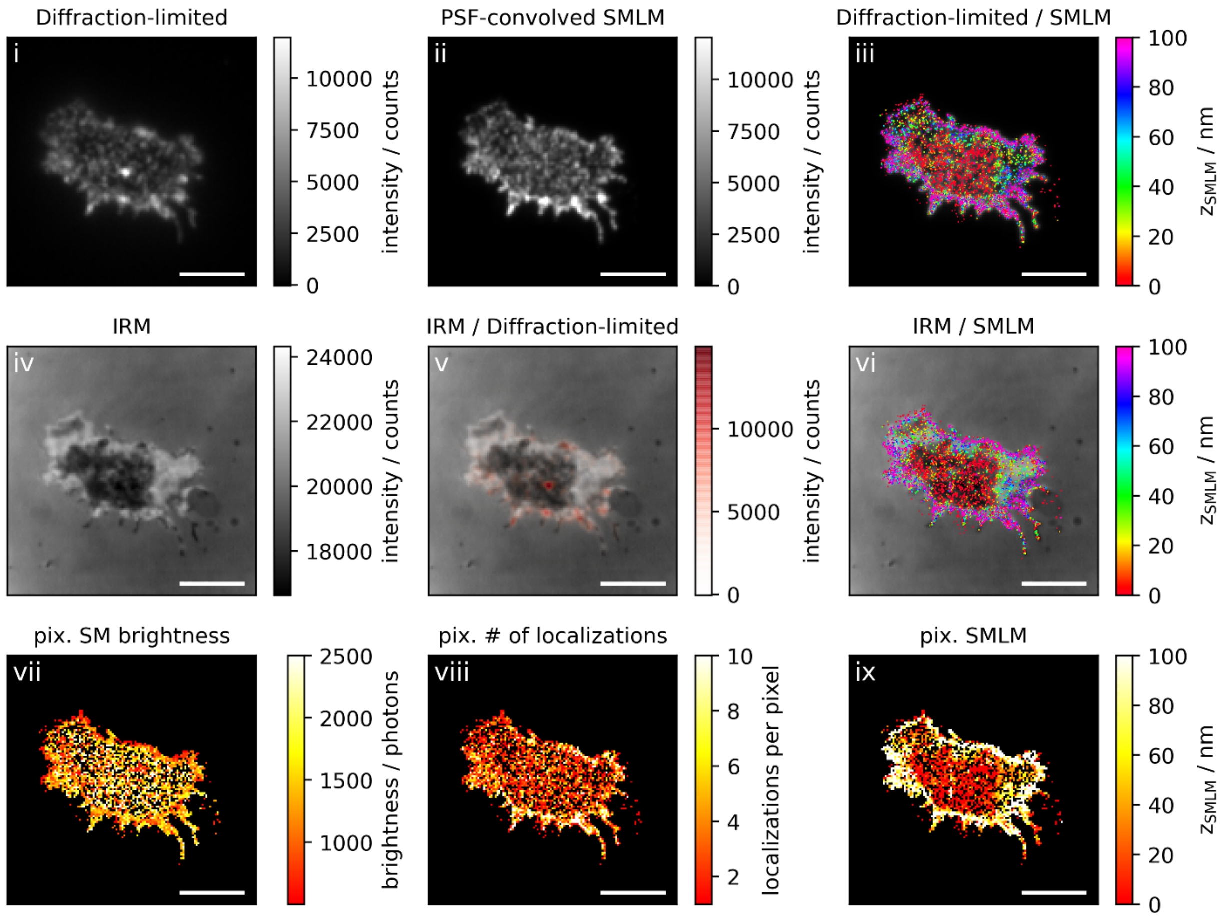
Diffraction-limited
i
intensity / counts
PSF-convolved SMLM
ii
Diffraction-limited / SMLM
iii
z_SMLM / nm
IRM
iv
IRM / Diffraction-limited
v
IRM / SMLM
vi
pix. SM brightness
vii
brightness / photons
pix. # of localizations
viii
localizations per pixel
pix. SMLM
ix

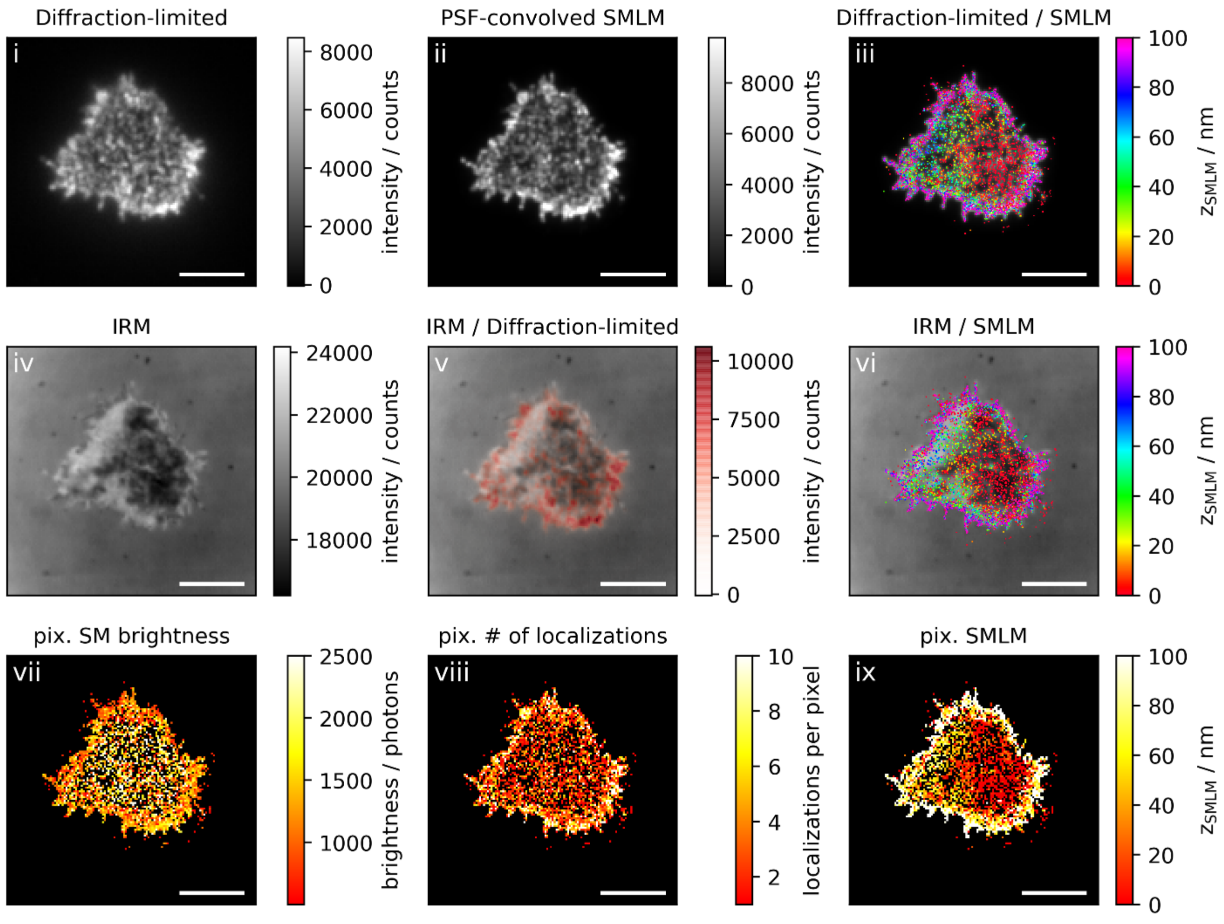

Diffraction-limited
i
intensity / counts
PSF-convolved SMLM
ii
Diffraction-limited / SMLM
iii
z_{SMLM} / nm
IRM
iv
IRM / Diffraction-limited
v
IRM / SMLM
vi
pix. SM brightness
vii
brightness / photons
pix. # of localizations
viii
localizations per pixel
pix. SMLM
ix

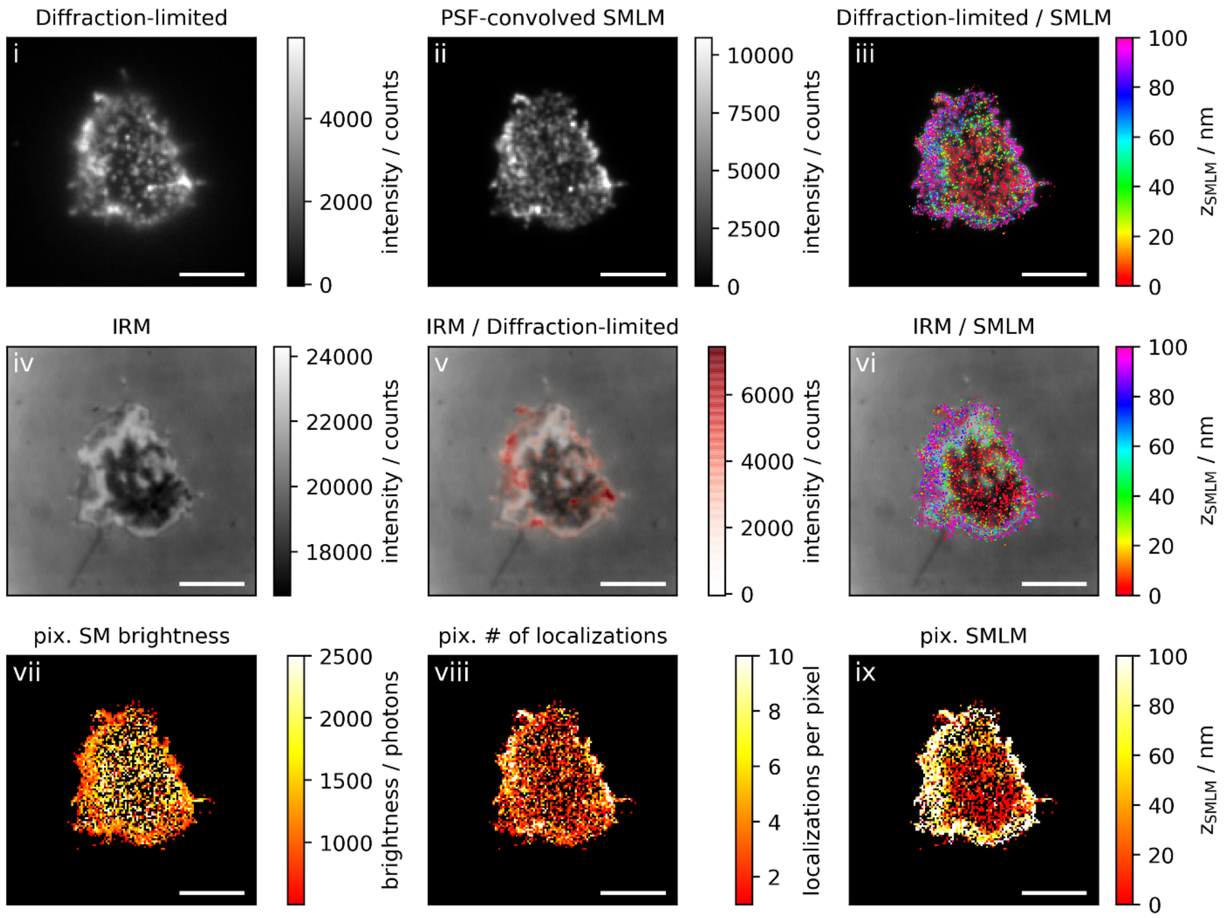
Diffraction-limited
i
4000
2000
0
intensity / counts
PSF-convolved SMLM
ii
10000
7500
5000
2500
0
intensity / counts
Diffraction-limited / SMLM
iii
100
80
60
40
20
0
z_{SMLM} / nm
IRM
iv
24000
22000
20000
18000
intensity / counts
IRM / Diffraction-limited
v
6000
4000
2000
0
intensity / counts
IRM / SMLM
vi
100
80
60
40
20
0
z_{SMLM} / nm
pix. SM brightness
vii
2500
2000
1500
1000
brightness / photons
pix. # of localizations
viii
10
8
6
4
2
localizations per pixel
pix. SMLM
ix
100
80
60
40
20
0
z_{SMLM} / nm

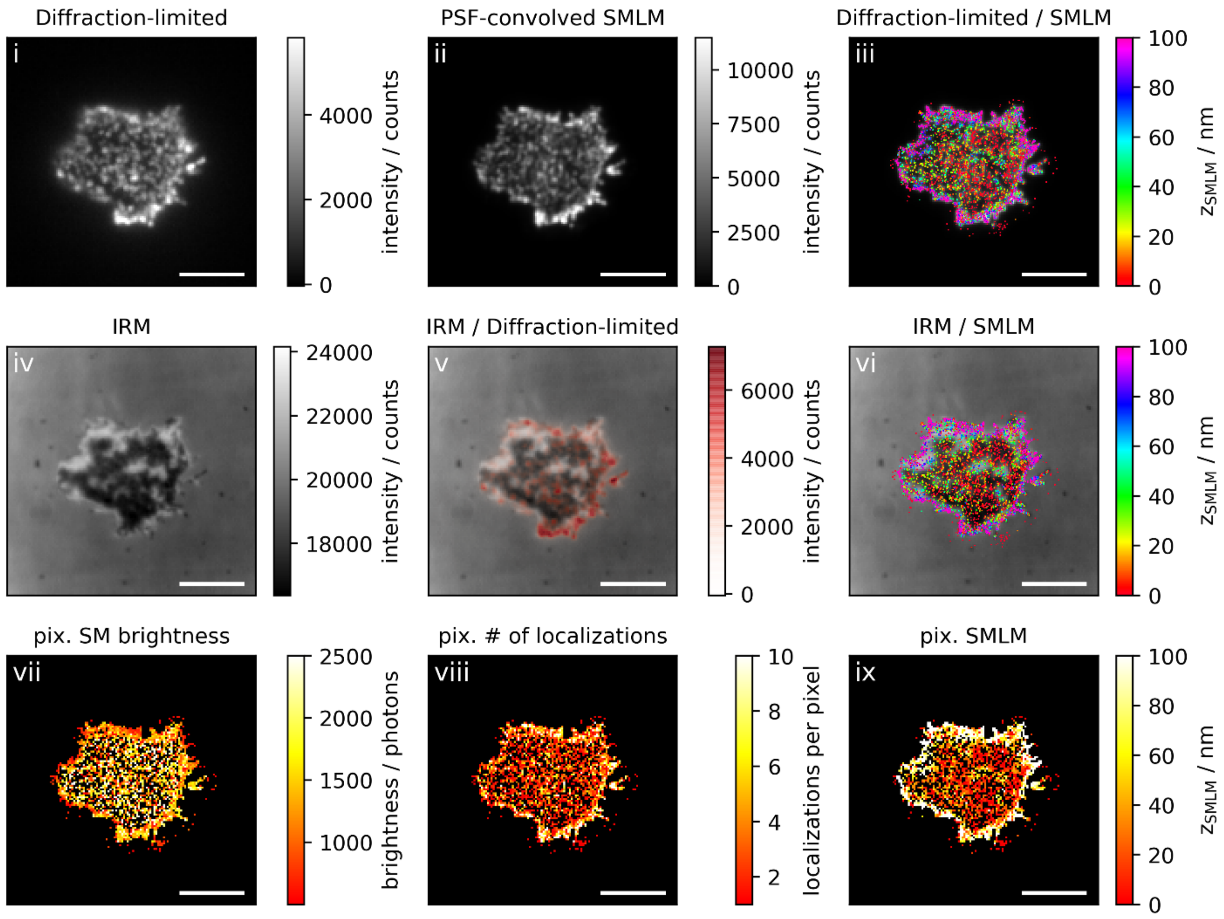

Diffraction-limited
i
4000
2000
0
intensity / counts
PSF-convolved SMLM
ii
10000
7500
5000
2500
0
intensity / counts
Diffraction-limited / SMLM
iii
100
80
60
40
20
0
zSMLM / nm
IRM
iv
24000
22000
20000
18000
intensity / counts
IRM / Diffraction-limited
v
6000
4000
2000
0
intensity / counts
IRM / SMLM
vi
100
80
60
40
20
0
zSMLM / nm
pix. SM brightness
vii
2500
2000
1500
1000
brightness / photons
pix. # of localizations
viii
10
8
6
4
2
localizations per pixel
pix. SMLM
ix
100
80
60
40
20
0
zSMLM / nm

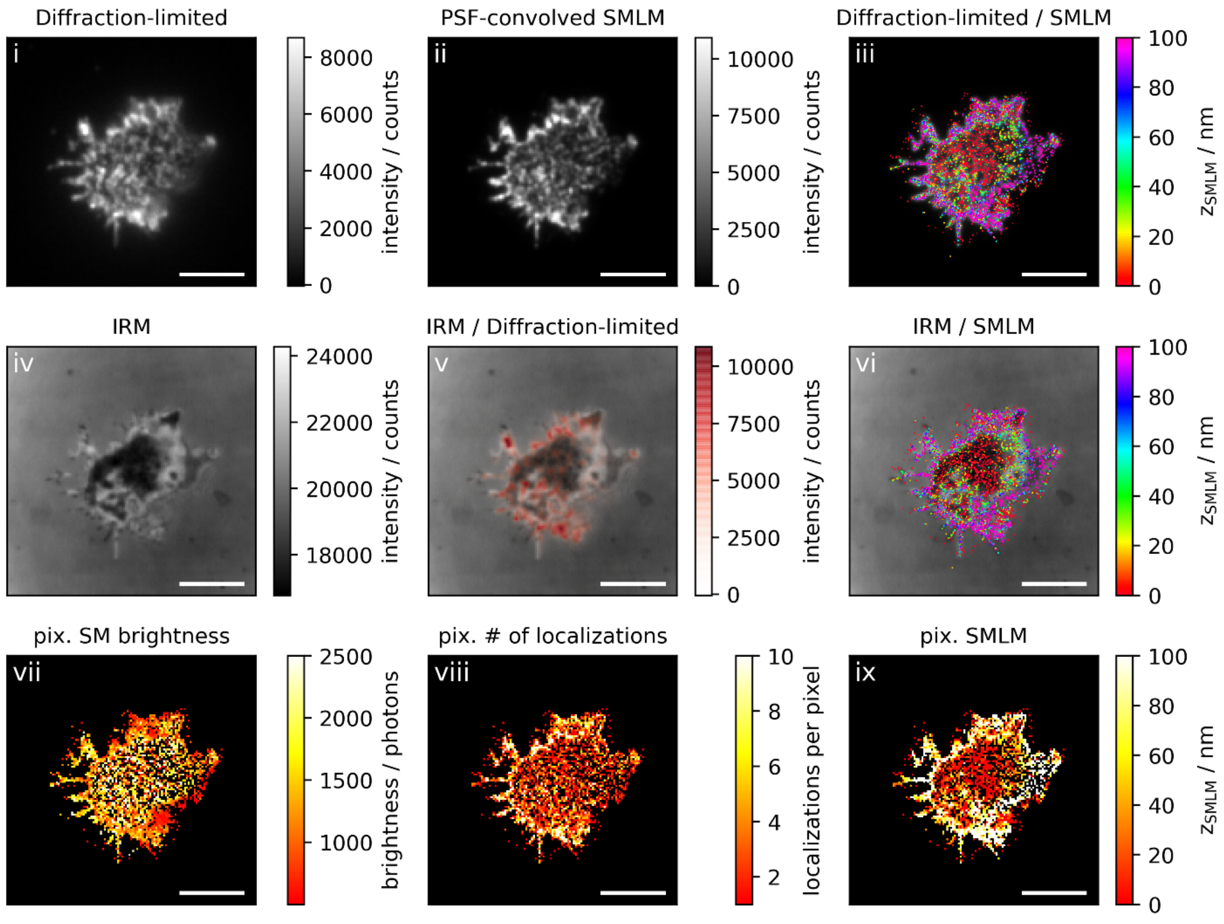
Diffraction-limited
i
intensity / counts
PSF-convolved SMLM
ii
intensity / counts
Diffraction-limited / SMLM
iii
z_{SMLM} / nm
IRM
iv
intensity / counts
IRM / Diffraction-limited
v
intensity / counts
IRM / SMLM
vi
z_{SMLM} / nm
pix. SM brightness
vii
brightness / photons
pix. # of localizations
viii
localizations per pixel
pix. SMLM
ix
z_{SMLM} / nm

Figure 3b: Non-activating conditions, high ICAM-1 density, fixation: 10 min post seeding

Correlative 3D-SMLM, IRM, and diffraction-limited TIR microscopy of the immunological synapse. T cells were seeded on an SLB functionalized with high density of ICAM-1, and fixed 10 minutes post seeding. The T cell was imaged with IRM and fluorescence microscopy: (i) Diffraction-limited TIR image of the T cell. (ii) Reconstruction of the diffraction-limited image by convolving the 3D-SMLM image with the corresponding psf. (iii) Overlay of the diffraction-limited TIR image with the 3D-SMLM image. Color-code indicates distance to the coverslip z_{SMLM}. (iv) IRM image. (v) Overlay of the IRM image with the diffraction-limited image. (vi) Overlay of the IRM and the 3D-SMLM image. Bottom row images were generated by calculating the pixel-wise average of the 3D-SMLM images (pixel size of 146nm is consistent with diffraction-limited image) according to pixelated mean single molecule (SM) intensity (vii), pixelated number of localizations (viii) and pixelated mean z_{SMLM} (ix). Scale bars 5 µm.

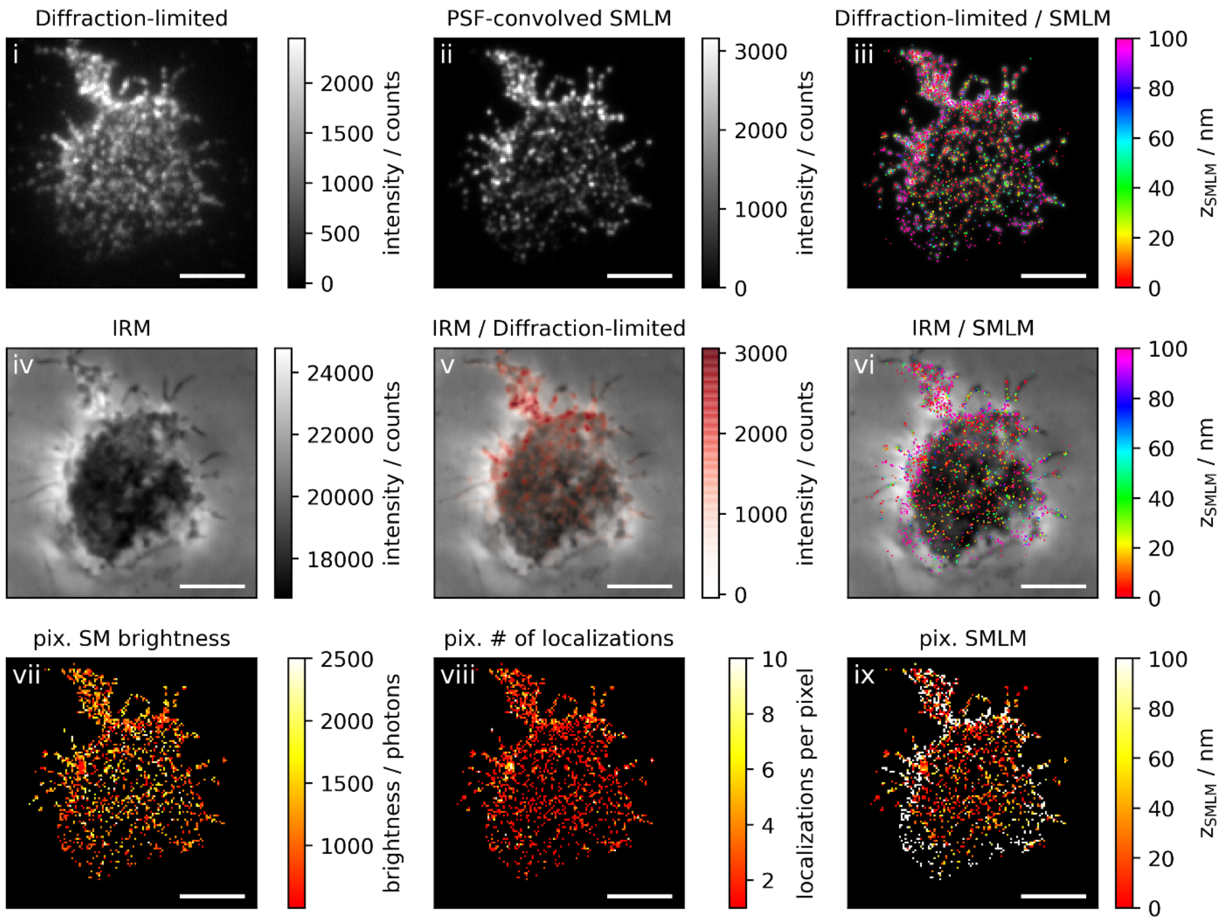
Diffraction-limited
i
2000
1500
1000
500
0
intensity / counts
PSF-convolved SMLM
ii
3000
2000
1000
0
intensity / counts
Diffraction-limited / SMLM
iii
100
80
60
40
20
0
z_{SMLM} / nm
IRM
iv
24000
22000
20000
18000
intensity / counts
IRM / Diffraction-limited
v
3000
2000
1000
0
intensity / counts
IRM / SMLM
vi
100
80
60
40
20
0
z_{SMLM} / nm
pix. SM brightness
vii
2500
2000
1500
1000
brightness / photons
pix. # of localizations
viii
10
8
6
4
2
localizations per pixel
pix. SMLM
ix
100
80
60
40
20
0
z_{SMLM} / nm

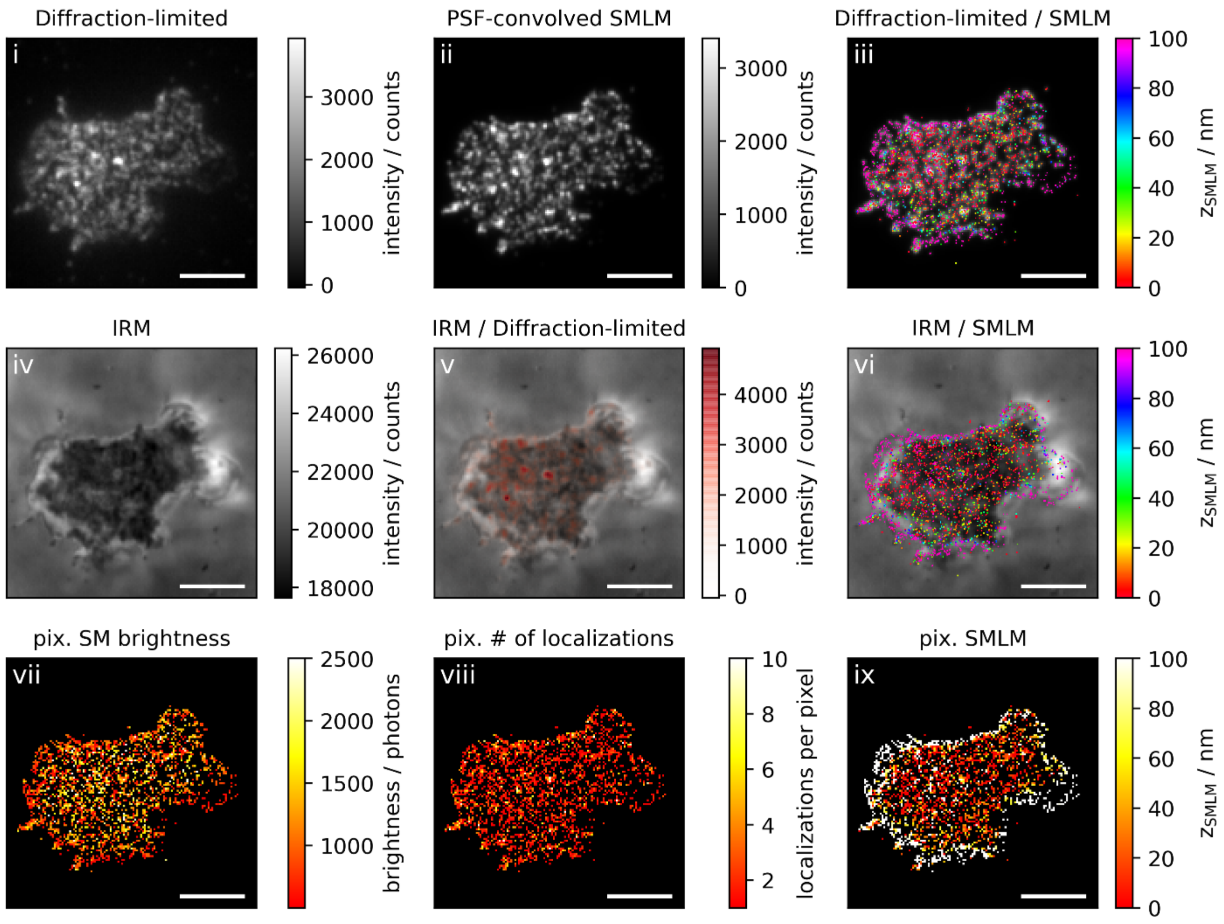
Diffraction-limited
i
3000
2000
1000
0
intensity / counts
PSF-convolved SMLM
ii
3000
2000
1000
0
intensity / counts
Diffraction-limited / SMLM
iii
100
80
60
40
20
0
zSMLM / nm
IRM
iv
26000
24000
22000
20000
18000
intensity / counts
IRM / Diffraction-limited
v
4000
3000
2000
1000
0
intensity / counts
IRM / SMLM
vi
100
80
60
40
20
0
zSMLM / nm
pix. SM brightness
vii
2500
2000
1500
1000
brightness / photons
pix. # of localizations
viii
10
8
6
4
2
localizations per pixel
pix. SMLM
ix
100
80
60
40
20
0
zSMLM / nm

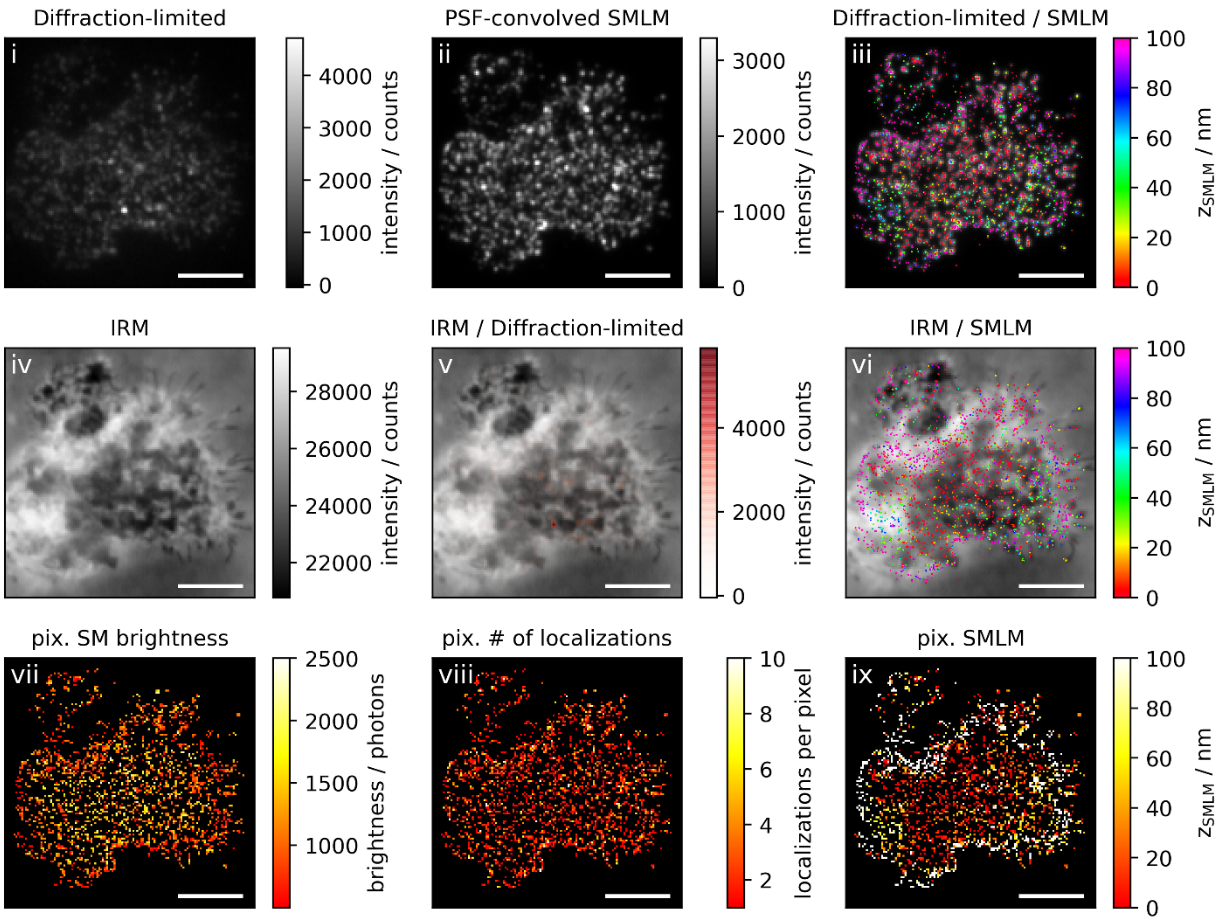

Diffraction-limited
i
intensity / counts
PSF-convolved SMLM
ii
Diffraction-limited / SMLM
iii
z_{SMLM} / nm
IRM
iv
IRM / Diffraction-limited
v
IRM / SMLM
vi
pix. SM brightness
vii
brightness / photons
pix. # of localizations
viii
localizations per pixel
pix. SMLM
ix

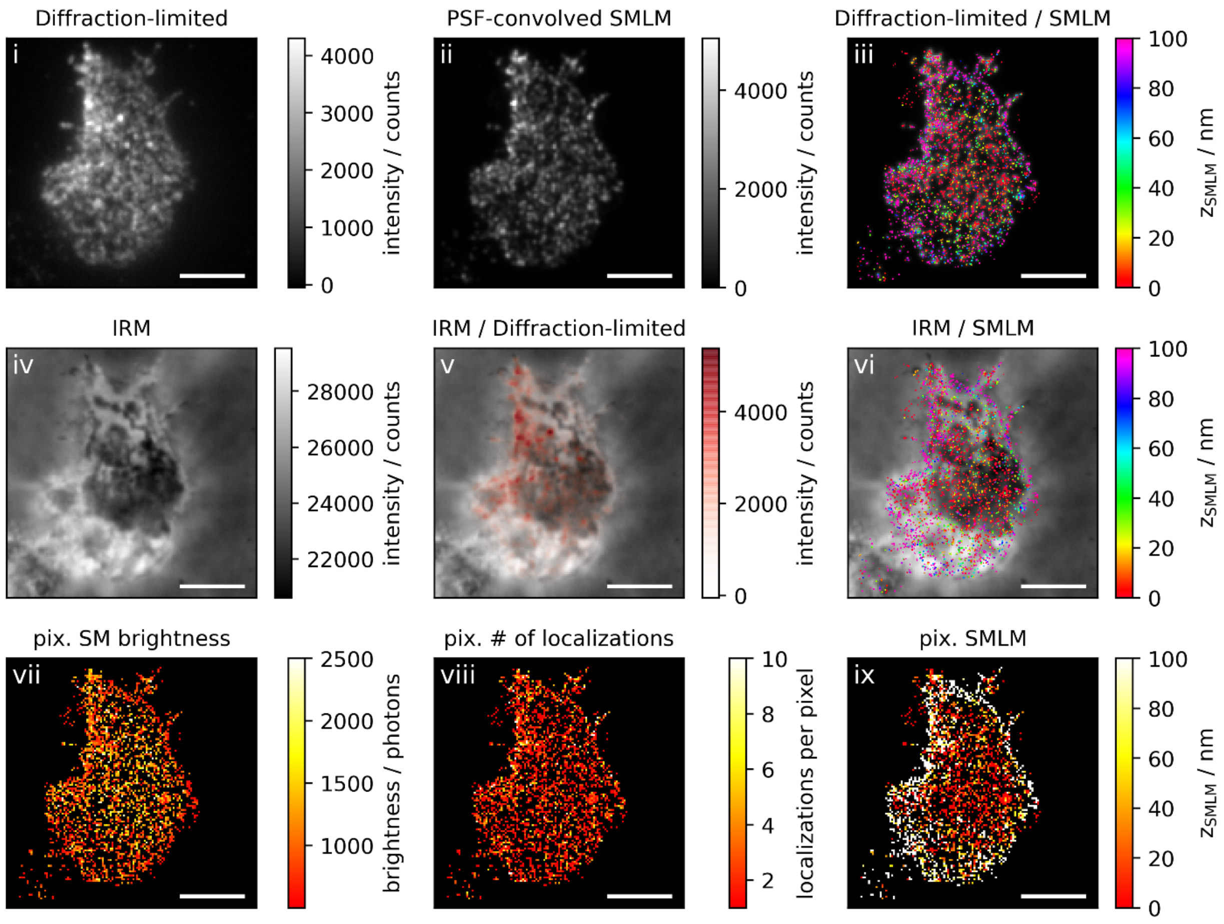
Diffraction-limited
i
4000
3000
2000
1000
0
intensity / counts
PSF-convolved SMLM
ii
4000
2000
0
intensity / counts
Diffraction-limited / SMLM
iii
100
80
60
40
20
0
z_SMLM / nm
IRM
iv
28000
26000
24000
22000
intensity / counts
IRM / Diffraction-limited
v
4000
2000
0
intensity / counts
IRM / SMLM
vi
100
80
60
40
20
0
z_SMLM / nm
pix. SM brightness
vii
2500
2000
1500
1000
brightness / photons
pix. # of localizations
viii
10
8
6
4
2
localizations per pixel
pix. SMLM
ix
100
80
60
40
20
0
z_SMLM / nm

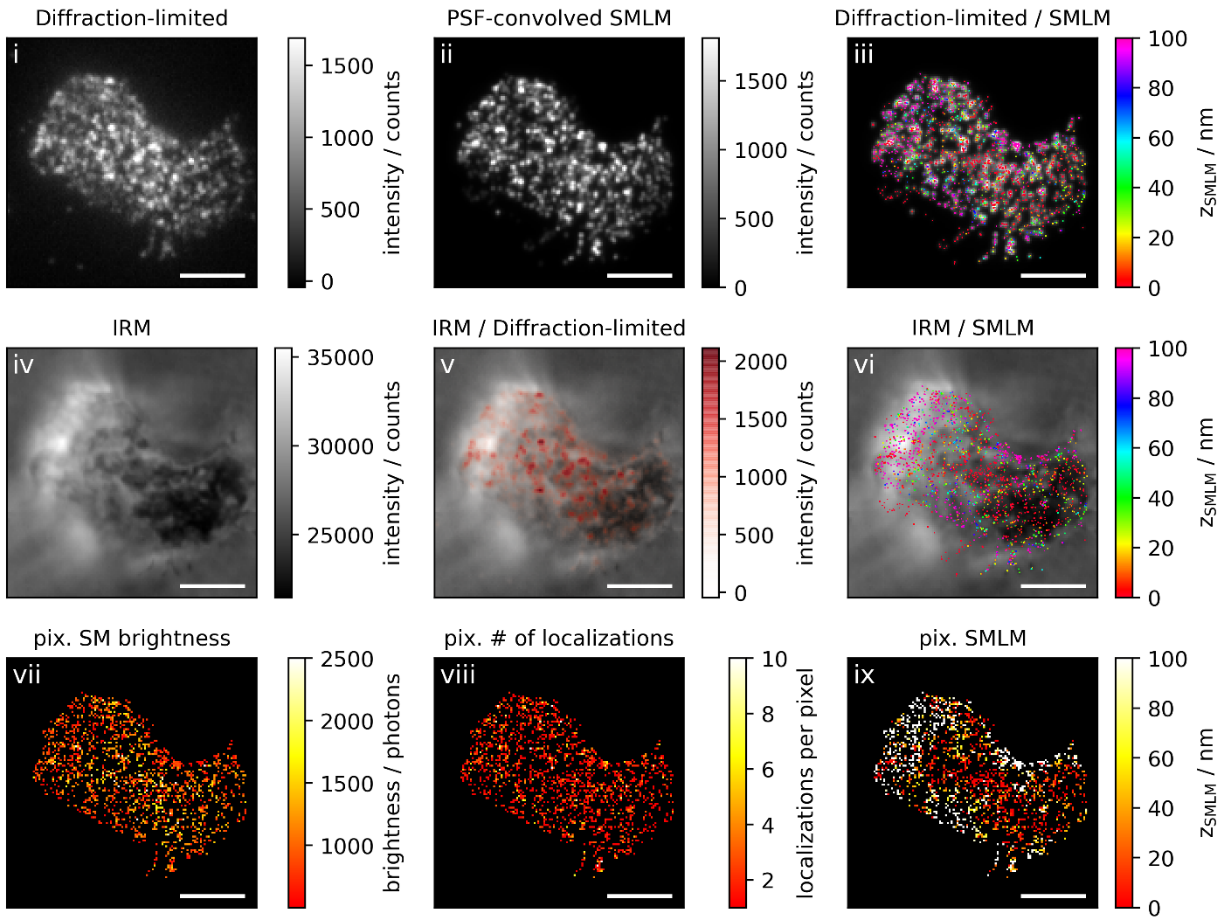

Diffraction-limited
i
intensity / counts
PSF-convolved SMLM
ii
intensity / counts
Diffraction-limited / SMLM
iii
z_{SMLM} / nm
IRM
iv
intensity / counts
IRM / Diffraction-limited
v
intensity / counts
IRM / SMLM
vi
z_{SMLM} / nm
pix. SM brightness
vii
brightness / photons
pix. # of localizations
viii
localizations per pixel
pix. SMLM
ix
z_{SMLM} / nm

Figure 3c: Non-activating conditions, high ICAM-1 density, fixation: 10-15 min post seeding

Correlative 3D-SMLM, IRM, and diffraction-limited TIR microscopy of the immunological synapse. T cells were seeded on an SLB functionalized with high density of ICAM-1, and fixed 10-15 minutes post seeding. The T cell was imaged with IRM and fluorescence microscopy: (i) Diffraction-limited TIR image of the T cell. (ii) Reconstruction of the diffraction-limited image by convolving the 3D-SMLM image with the corresponding psf. (iii) Overlay of the diffraction-limited TIR image with the 3D-SMLM image. Color-code indicates distance to the coverslip z_{SMLM}. (iv) IRM image. (v) Overlay of the IRM image with the diffraction-limited image. (vi) Overlay of the IRM and the 3D-SMLM image. Bottom row images were generated by calculating the pixel-wise average of the 3D-SMLM images (pixel size of 146nm is consistent with diffraction-limited image) according to pixelated mean single molecule (SM) intensity (vii), pixelated number of localizations (viii) and pixelated mean z_{SMLM} (ix). Scale bars 5 µm.

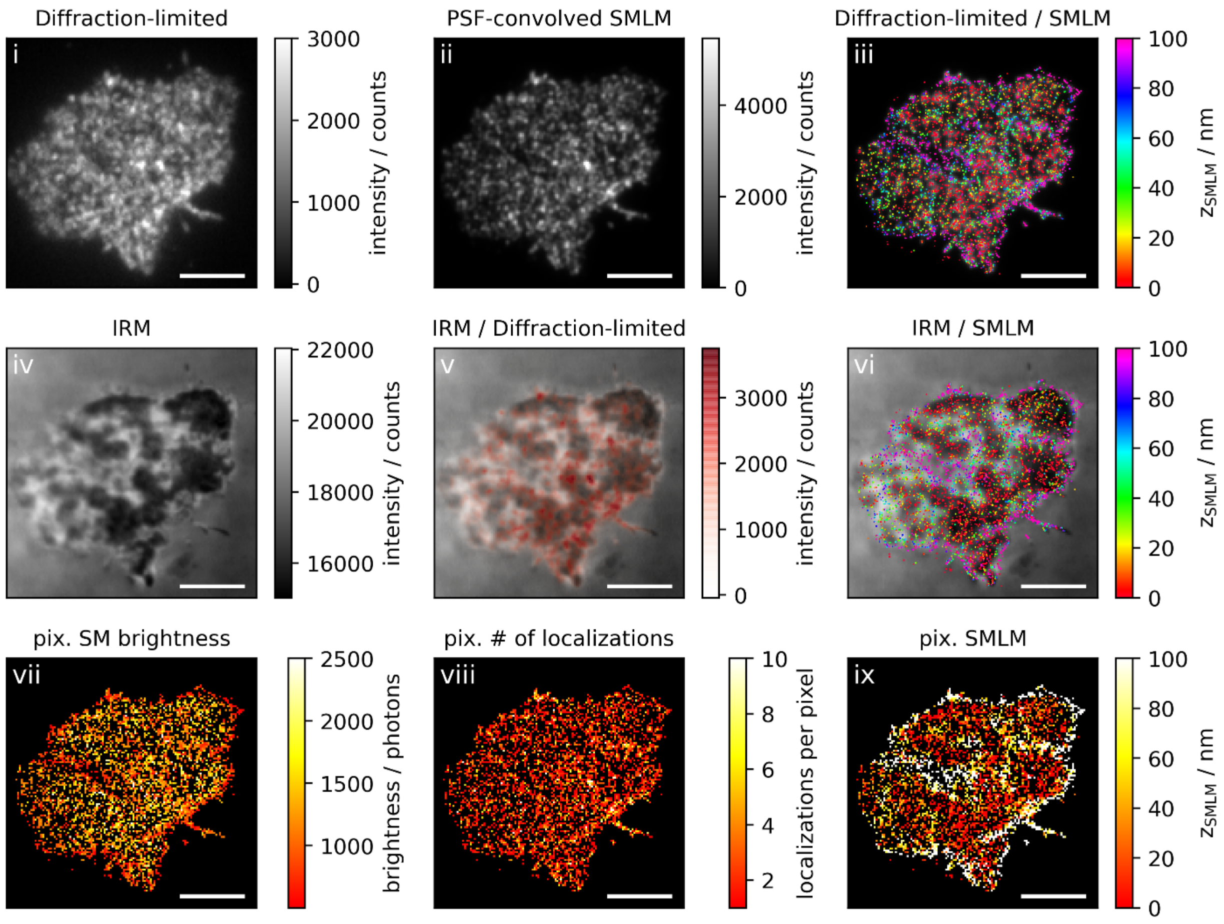

Diffraction-limited
i
3000
2000
1000
0
intensity / counts
PSF-convolved SMLM
ii
4000
2000
0
intensity / counts
Diffraction-limited / SMLM
iii
100
80
60
40
20
0
z_{SMLM} / nm
IRM
iv
22000
20000
18000
16000
intensity / counts
IRM / Diffraction-limited
v
3000
2000
1000
0
intensity / counts
IRM / SMLM
vi
100
80
60
40
20
0
z_{SMLM} / nm
pix. SM brightness
vii
2500
2000
1500
1000
brightness / photons
pix. # of localizations
viii
10
8
6
4
2
localizations per pixel
pix. SMLM
ix
100
80
60
40
20
0
z_{SMLM} / nm

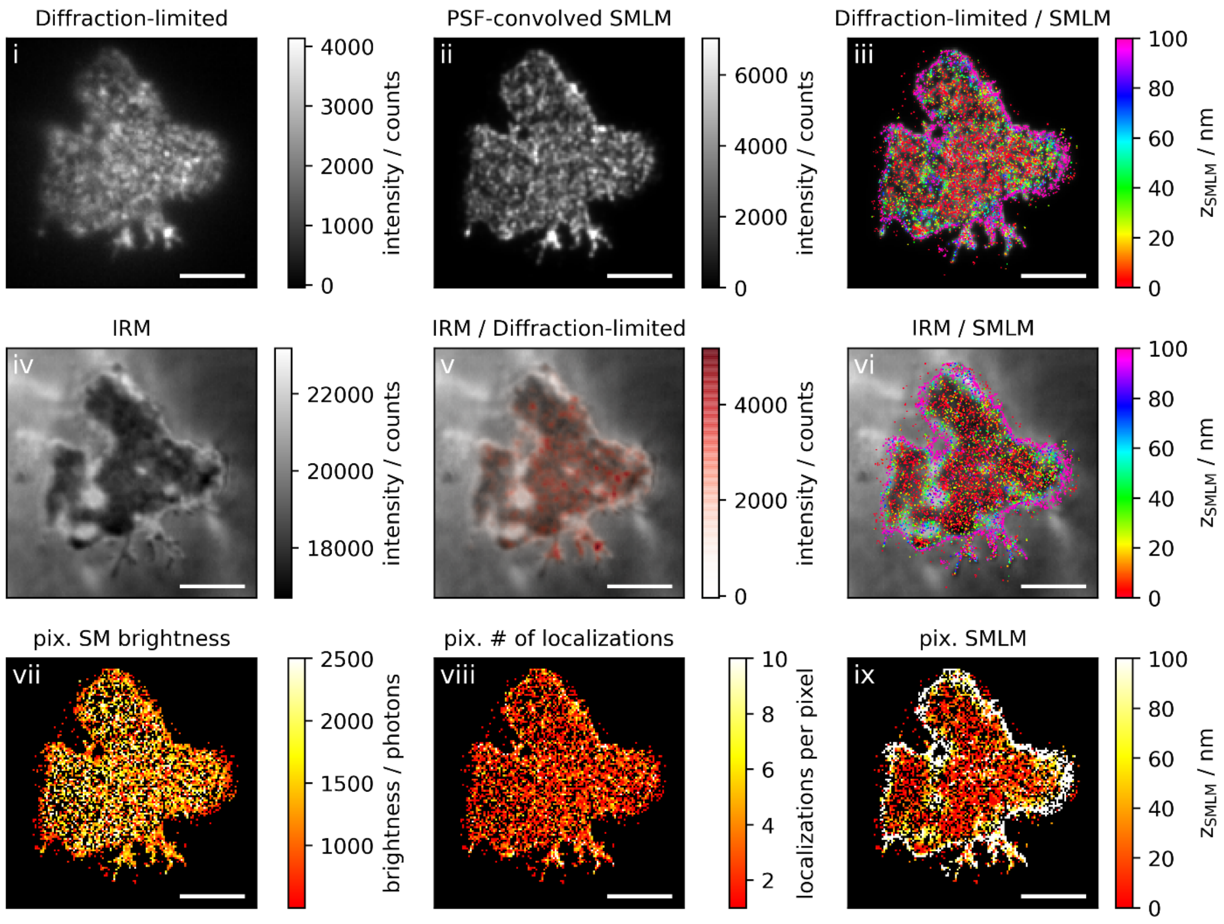

Diffraction-limited
i
intensity / counts
0
1000
2000
3000
4000
PSF-convolved SMLM
ii
intensity / counts
0
2000
4000
6000
Diffraction-limited / SMLM
iii
z_{SMLM} / nm
0
20
40
60
80
100
IRM
iv
intensity / counts
18000
20000
22000
IRM / Diffraction-limited
v
intensity / counts
0
2000
4000
IRM / SMLM
vi
z_{SMLM} / nm
0
20
40
60
80
100
pix. SM brightness
vii
brightness / photons
1000
1500
2000
2500
pix. # of localizations
viii
localizations per pixel
2
4
6
8
10
pix. SMLM
ix
z_{SMLM} / nm
0
20
40
60
80
100

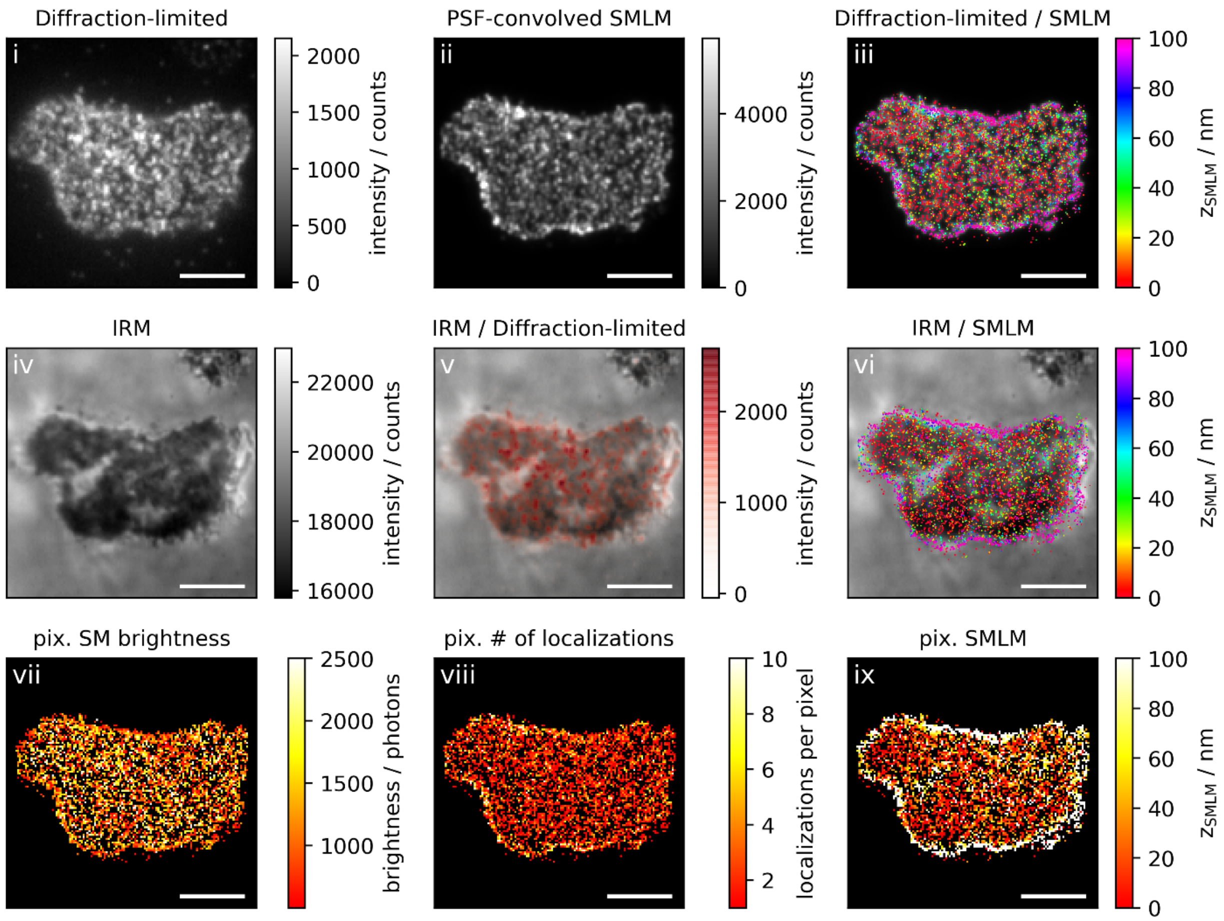
Diffraction-limited
i
2000
1500
1000
500
0
intensity / counts
PSF-convolved SMLM
ii
4000
2000
0
intensity / counts
Diffraction-limited / SMLM
iii
100
80
60
40
20
0
z_{SMLM} / nm
IRM
iv
22000
20000
18000
16000
intensity / counts
IRM / Diffraction-limited
v
2000
1000
0
intensity / counts
IRM / SMLM
vi
100
80
60
40
20
0
z_{SMLM} / nm
pix. SM brightness
vii
2500
2000
1500
1000
brightness / photons
pix. # of localizations
viii
10
8
6
4
2
localizations per pixel
pix. SMLM
ix
100
80
60
40
20
0
z_{SMLM} / nm

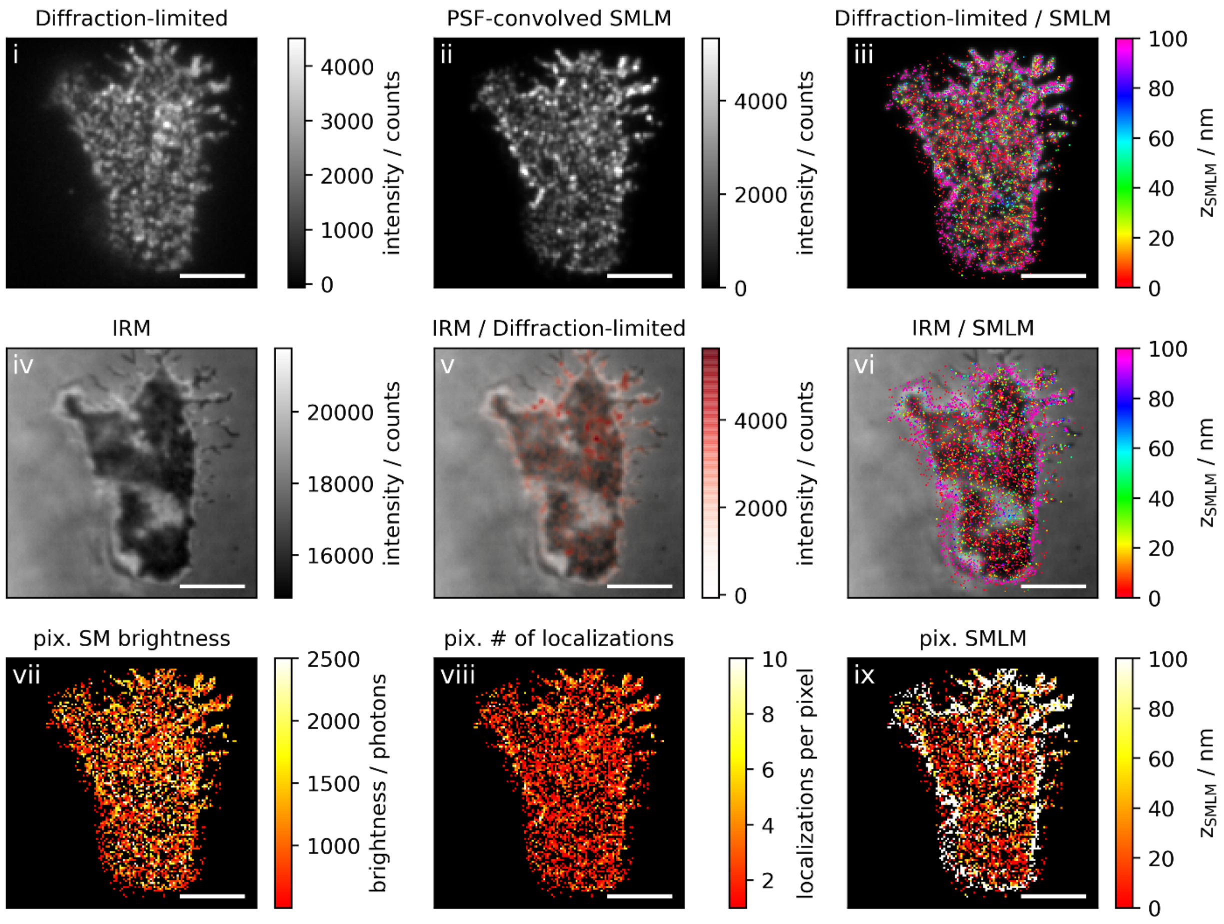

Diffraction-limited
i
intensity / counts
0
1000
2000
3000
4000
PSF-convolved SMLM
ii
intensity / counts
0
2000
4000
Diffraction-limited / SMLM
iii
z_{SMLM} / nm
0
20
40
60
80
100
IRM
iv
intensity / counts
16000
18000
20000
IRM / Diffraction-limited
v
intensity / counts
0
2000
4000
IRM / SMLM
vi
z_{SMLM} / nm
0
20
40
60
80
100
pix. SM brightness
vii
brightness / photons
1000
1500
2000
2500
pix. # of localizations
viii
localizations per pixel
2
4
6
8
10
pix. SMLM
ix
z_{SMLM} / nm
0
20
40
60
80
100

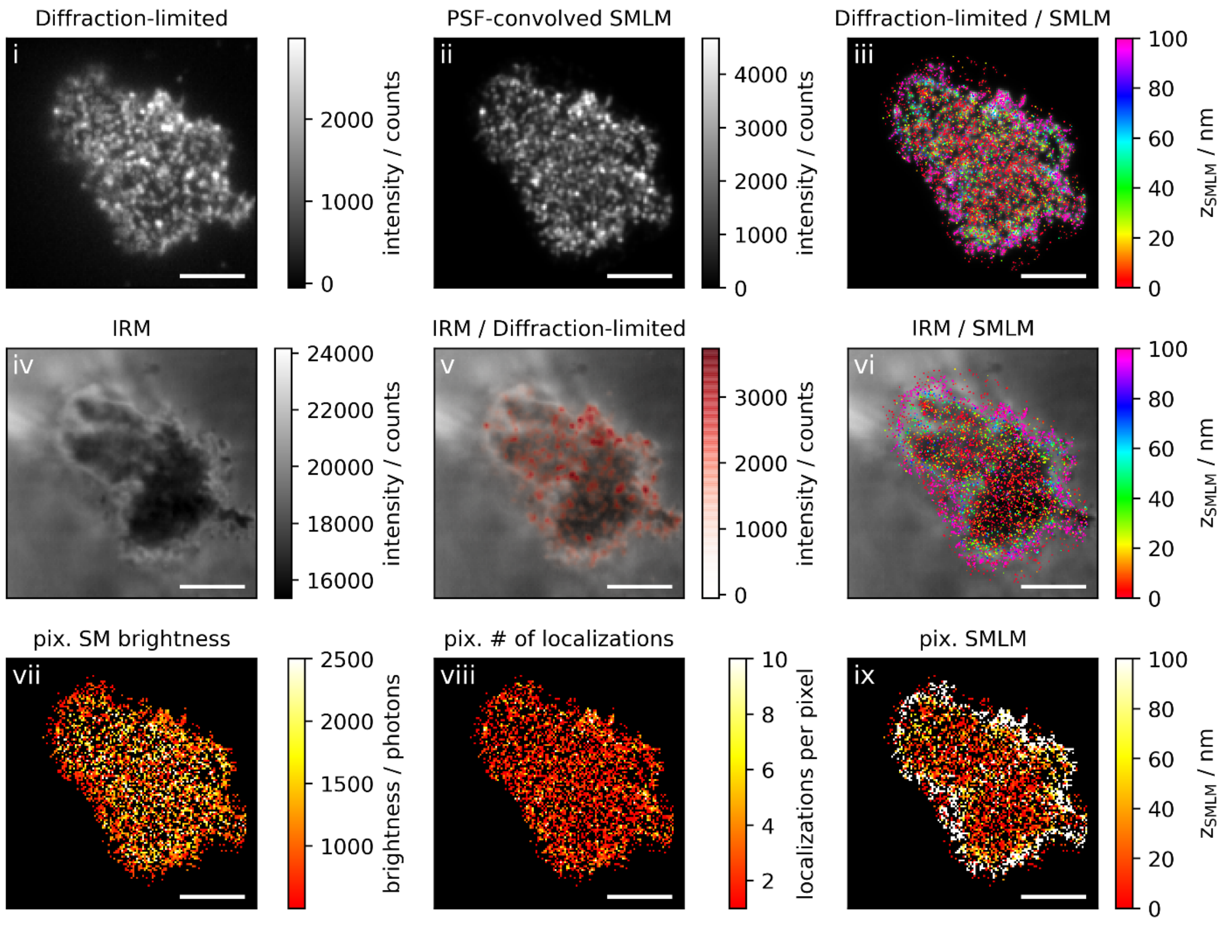

Diffraction-limited
i
intensity / counts
0
1000
2000
PSF-convolved SMLM
ii
intensity / counts
0
1000
2000
3000
4000
Diffraction-limited / SMLM
iii
z_{SMLM} / nm
0
20
40
60
80
100
IRM
iv
intensity / counts
16000
18000
20000
22000
24000
IRM / Diffraction-limited
v
intensity / counts
0
1000
2000
3000
IRM / SMLM
vi
z_{SMLM} / nm
0
20
40
60
80
100
pix. SM brightness
vii
brightness / photons
1000
1500
2000
2500
pix. # of localizations
viii
localizations per pixel
2
4
6
8
10
pix. SMLM
ix
z_{SMLM} / nm
0
20
40
60
80
100

Figure 4a: Non-activating conditions, low ICAM-1 density, fixation: 5-10 min post seeding

Correlative 3D-SMLM, IRM, and diffraction-limited TIR microscopy of the immunological synapse. T cells were seeded on an SLB functionalized with low density of ICAM-1, and fixed 5-10 minutes post seeding. The T cell was imaged with IRM and fluorescence microscopy: (i) Diffraction-limited TIR image of the T cell. (ii) Reconstruction of the diffraction-limited image by convolving the 3D-SMLM image with the corresponding psf. (iii) Overlay of the diffraction-limited TIR image with the 3D-SMLM image. Color-code indicates distance to the coverslip z_{SMLM}. (iv) IRM image. (v) Overlay of the IRM image with the diffraction-limited image. (vi) Overlay of the IRM and the 3D-SMLM image. Bottom row images were generated by calculating the pixel-wise average of the 3D-SMLM images (pixel size of 146nm is consistent with diffraction-limited image) according to pixelated mean single molecule (SM) intensity (vii), pixelated number of localizations (viii) and pixelated mean z_{SMLM} (ix). Scale bars 5 µm.

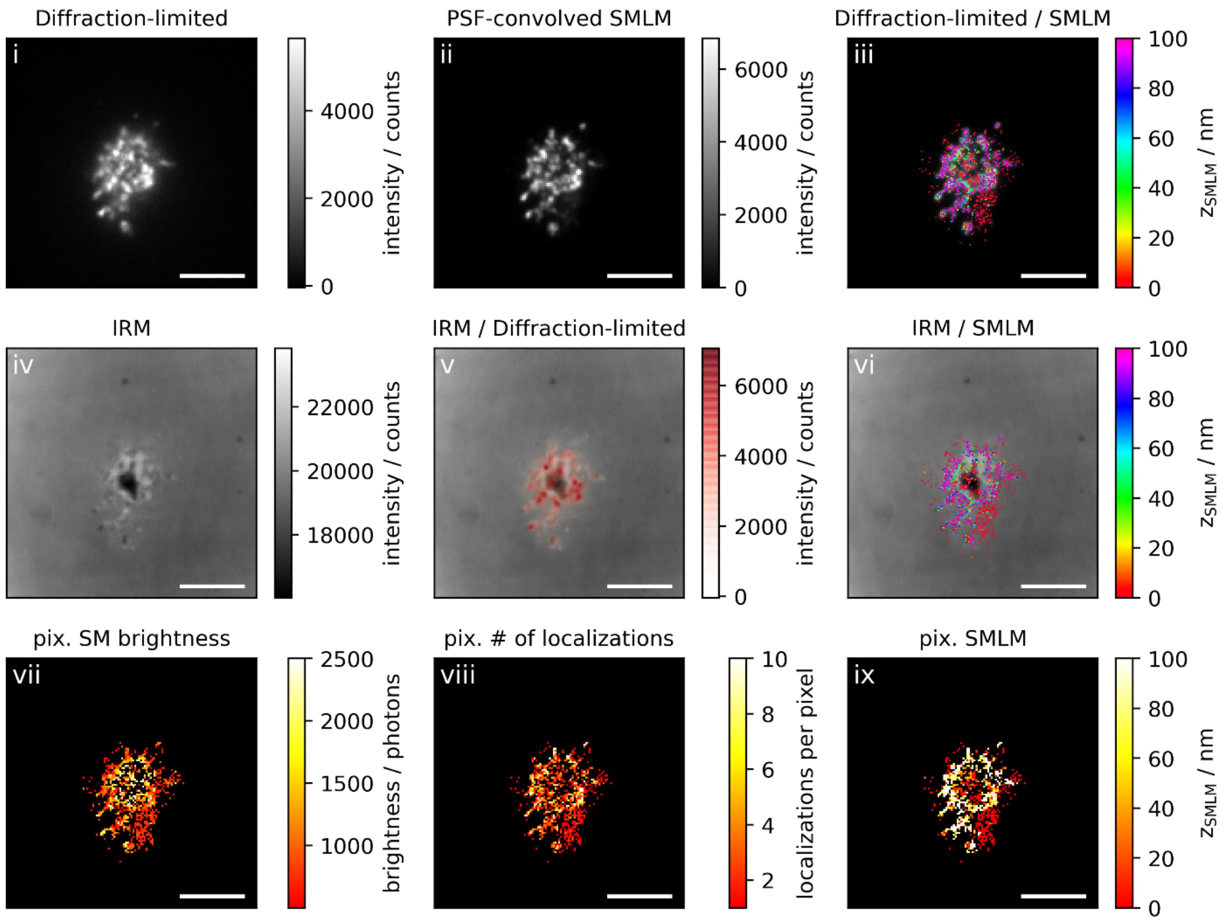
Diffraction-limited
i
intensity / counts
0
2000
4000
PSF-convolved SMLM
ii
intensity / counts
0
2000
4000
6000
Diffraction-limited / SMLM
iii
z_{SMLM} / nm
0
20
40
60
80
100
IRM
iv
intensity / counts
18000
20000
22000
IRM / Diffraction-limited
v
intensity / counts
0
2000
4000
6000
IRM / SMLM
vi
z_{SMLM} / nm
0
20
40
60
80
100
pix. SM brightness
vii
brightness / photons
1000
1500
2000
2500
pix. # of localizations
viii
localizations per pixel
2
4
6
8
10
pix. SMLM
ix
z_{SMLM} / nm
0
20
40
60
80
100

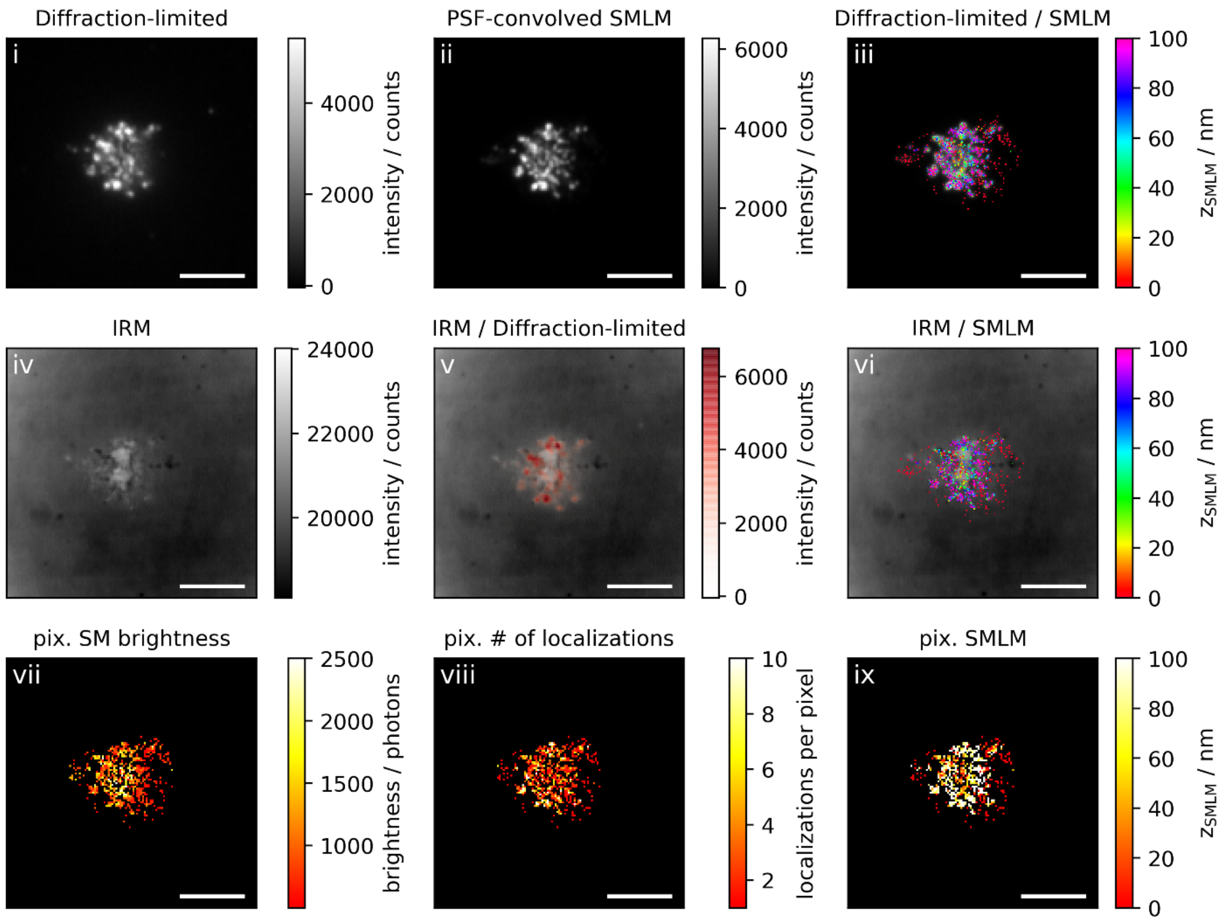
Diffraction-limited
i
0
2000
4000
intensity / counts
PSF-convolved SMLM
ii
0
2000
4000
6000
intensity / counts
Diffraction-limited / SMLM
iii
0
20
40
60
80
100
zSMLM / nm
IRM
iv
20000
22000
24000
intensity / counts
IRM / Diffraction-limited
v
0
2000
4000
6000
intensity / counts
IRM / SMLM
vi
0
20
40
60
80
100
zSMLM / nm
pix. SM brightness
vii
1000
1500
2000
2500
brightness / photons
pix. # of localizations
viii
2
4
6
8
10
localizations per pixel
pix. SMLM
ix
0
20
40
60
80
100
zSMLM / nm

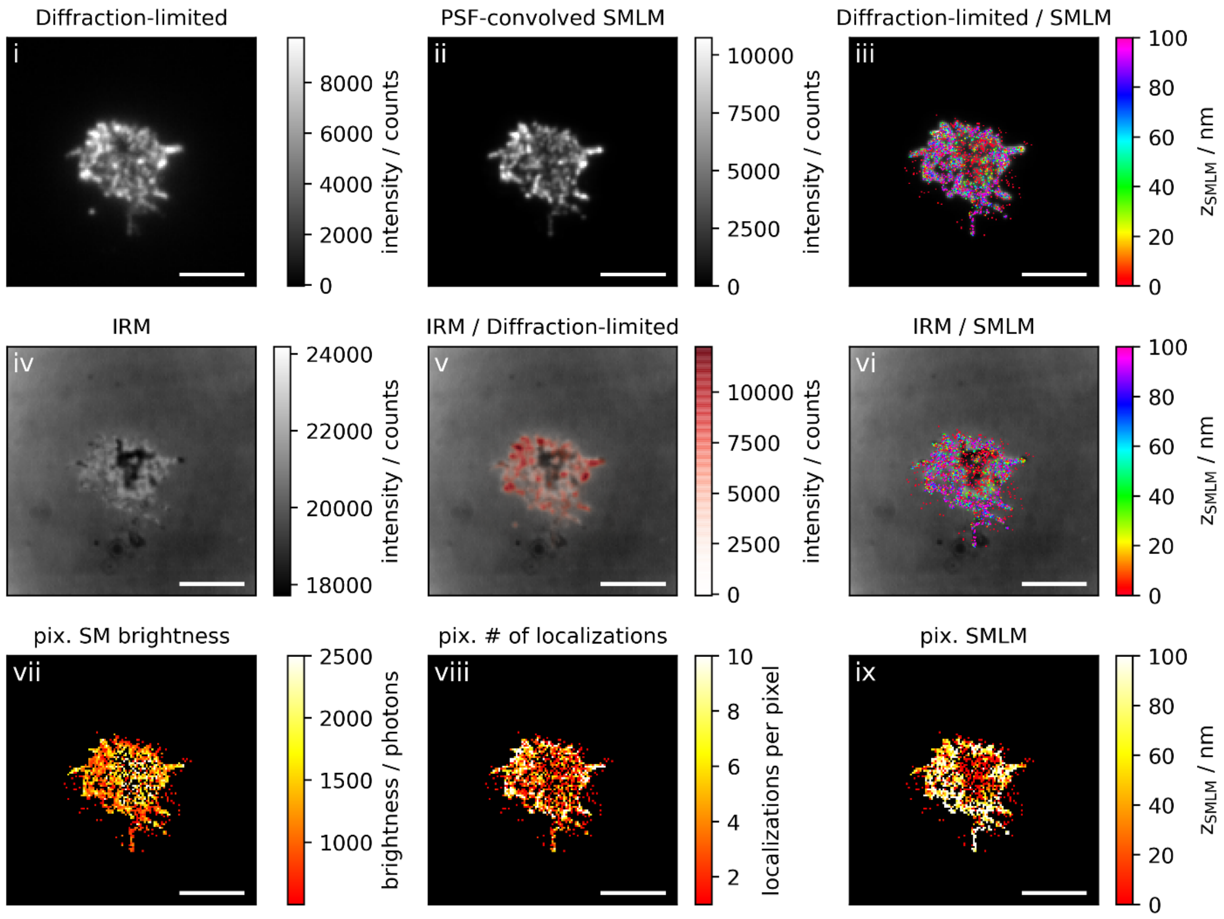
Diffraction-limited
i
intensity / counts
PSF-convolved SMLM
ii
intensity / counts
Diffraction-limited / SMLM
iii
z_{SMLM} / nm
IRM
iv
intensity / counts
IRM / Diffraction-limited
v
intensity / counts
IRM / SMLM
vi
z_{SMLM} / nm
pix. SM brightness
vii
brightness / photons
pix. # of localizations
viii
localizations per pixel
pix. SMLM
ix
z_{SMLM} / nm

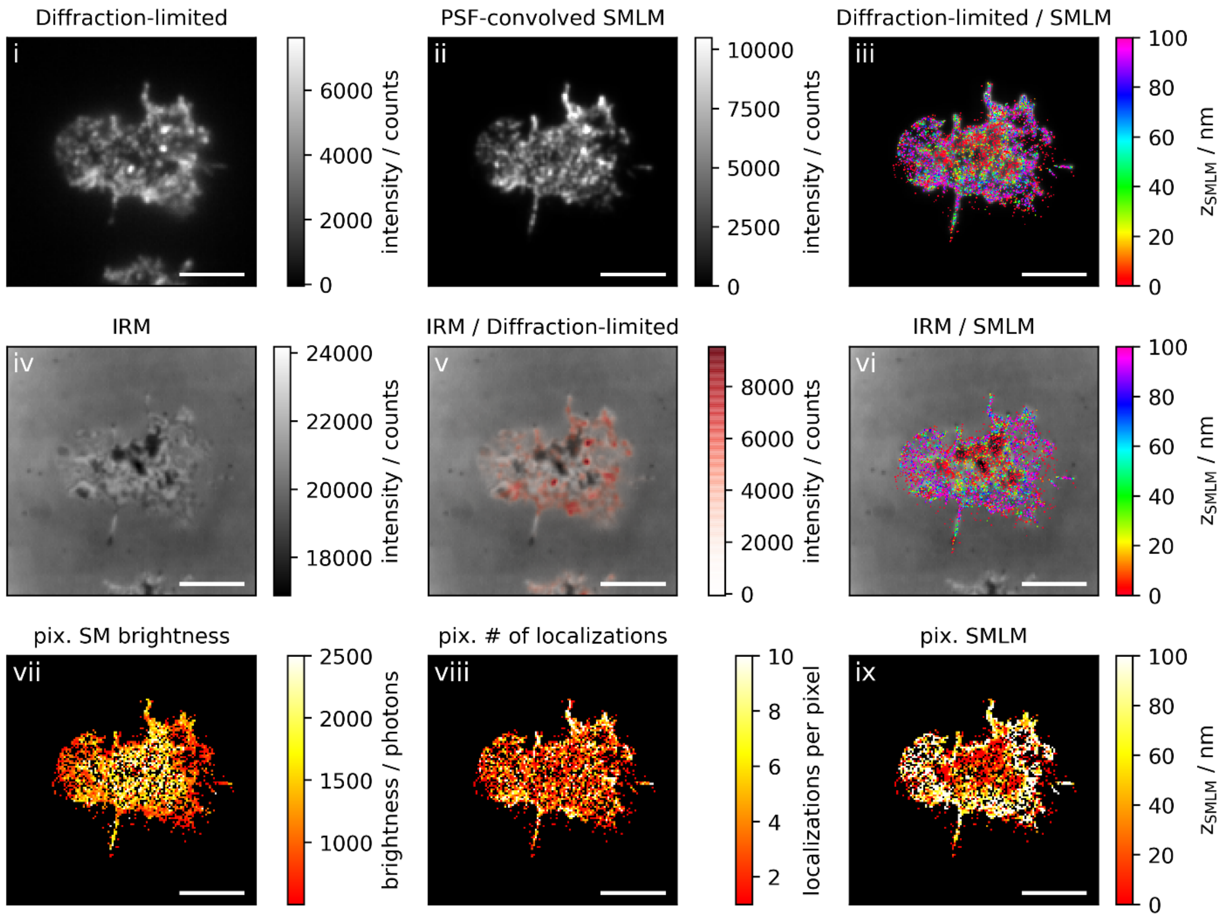
Diffraction-limited
i
6000
4000
2000
0
intensity / counts
PSF-convolved SMLM
ii
10000
7500
5000
2500
0
intensity / counts
Diffraction-limited / SMLM
iii
100
80
60
40
20
0
z_{SMLM} / nm
IRM
iv
24000
22000
20000
18000
intensity / counts
IRM / Diffraction-limited
v
8000
6000
4000
2000
0
intensity / counts
IRM / SMLM
vi
100
80
60
40
20
0
z_{SMLM} / nm
pix. SM brightness
vii
2500
2000
1500
1000
brightness / photons
pix. # of localizations
viii
10
8
6
4
2
localizations per pixel
pix. SMLM
ix
100
80
60
40
20
0
z_{SMLM} / nm

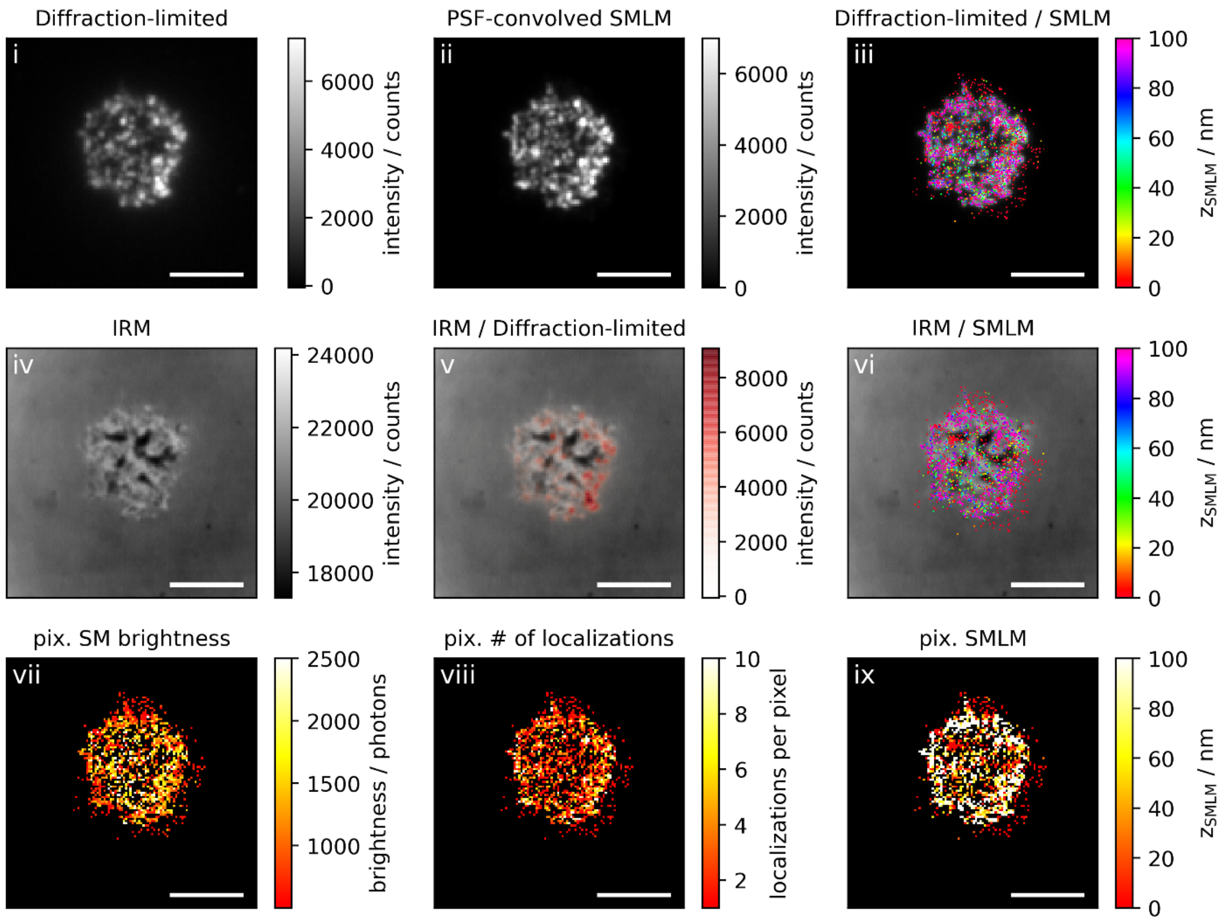
Diffraction-limited
i
intensity / counts
PSF-convolved SMLM
ii
Diffraction-limited / SMLM
iii
z_{SMLM} / nm
IRM
iv
IRM / Diffraction-limited
v
IRM / SMLM
vi
pix. SM brightness
vii
brightness / photons
pix. # of localizations
viii
localizations per pixel
pix. SMLM
ix

Figure 4b: Non-activating conditions, low ICAM-1 density, fixation: 10 min post seeding

Correlative 3D-SMLM, IRM, and diffraction-limited TIR microscopy of the immunological synapse. T cells were seeded on an SLB functionalized with low density of ICAM-1, and fixed 10 minutes post seeding. The T cell was imaged with IRM and fluorescence microscopy: (i) Diffraction-limited TIR image of the T cell. (ii) Reconstruction of the diffraction-limited image by convolving the 3D-SMLM image with the corresponding psf. (iii) Overlay of the diffraction-limited TIR image with the 3D-SMLM image. Color-code indicates distance to the coverslip z_{SMLM}. (iv) IRM image. (v) Overlay of the IRM image with the diffraction-limited image. (vi) Overlay of the IRM and the 3D-SMLM image. Bottom row images were generated by calculating the pixel-wise average of the 3D-SMLM images (pixel size of 146nm is consistent with diffraction-limited image) according to pixelated mean single molecule (SM) intensity (vii), pixelated number of localizations (viii) and pixelated mean z_{SMLM} (ix). Scale bars 5 µm.

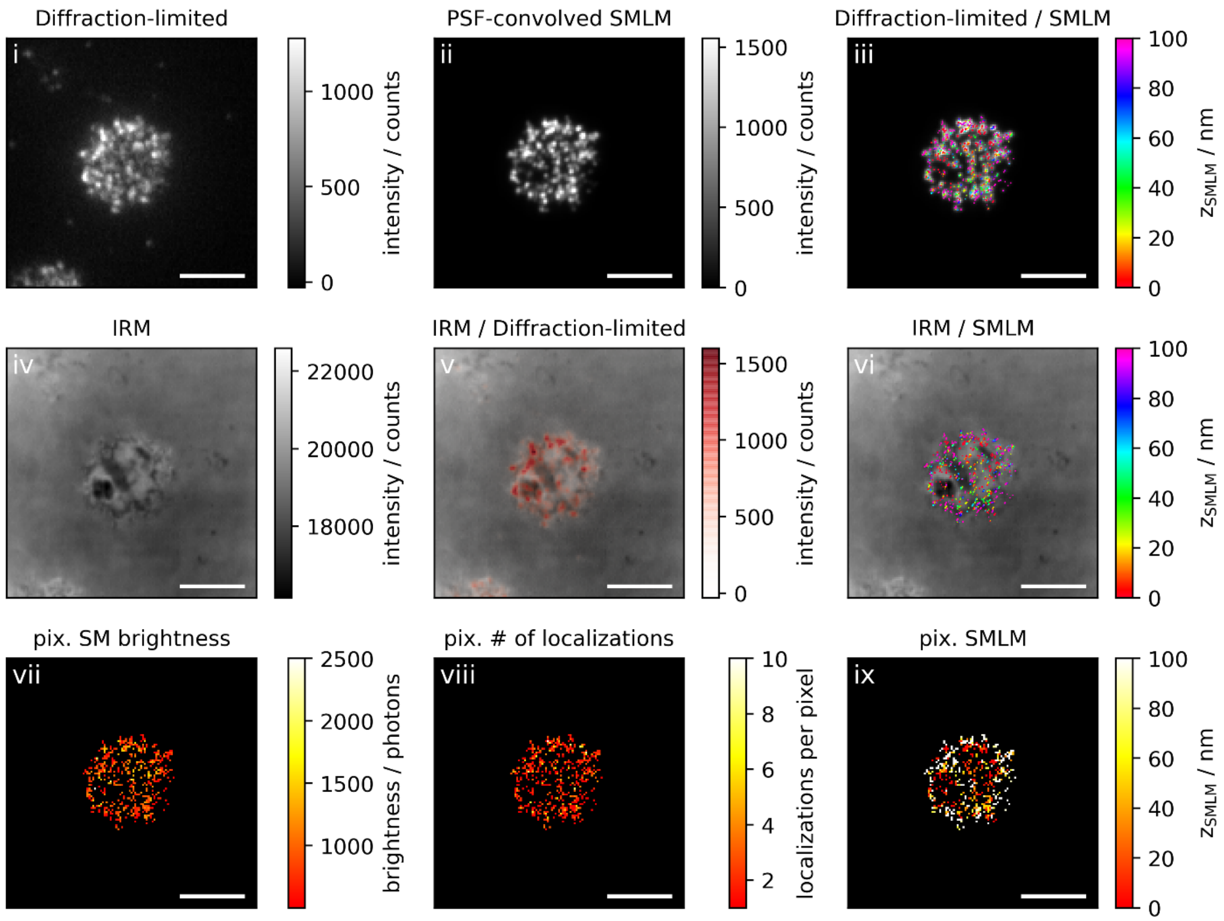

Diffraction-limited
i
1000
500
0
intensity / counts
PSF-convolved SMLM
ii
1500
1000
500
0
intensity / counts
Diffraction-limited / SMLM
iii
100
80
60
40
20
0
z_SMLM / nm
IRM
iv
22000
20000
18000
intensity / counts
IRM / Diffraction-limited
v
1500
1000
500
0
intensity / counts
IRM / SMLM
vi
100
80
60
40
20
0
z_SMLM / nm
pix. SM brightness
vii
2500
2000
1500
1000
brightness / photons
pix. # of localizations
viii
10
8
6
4
2
localizations per pixel
pix. SMLM
ix
100
80
60
40
20
0
z_SMLM / nm

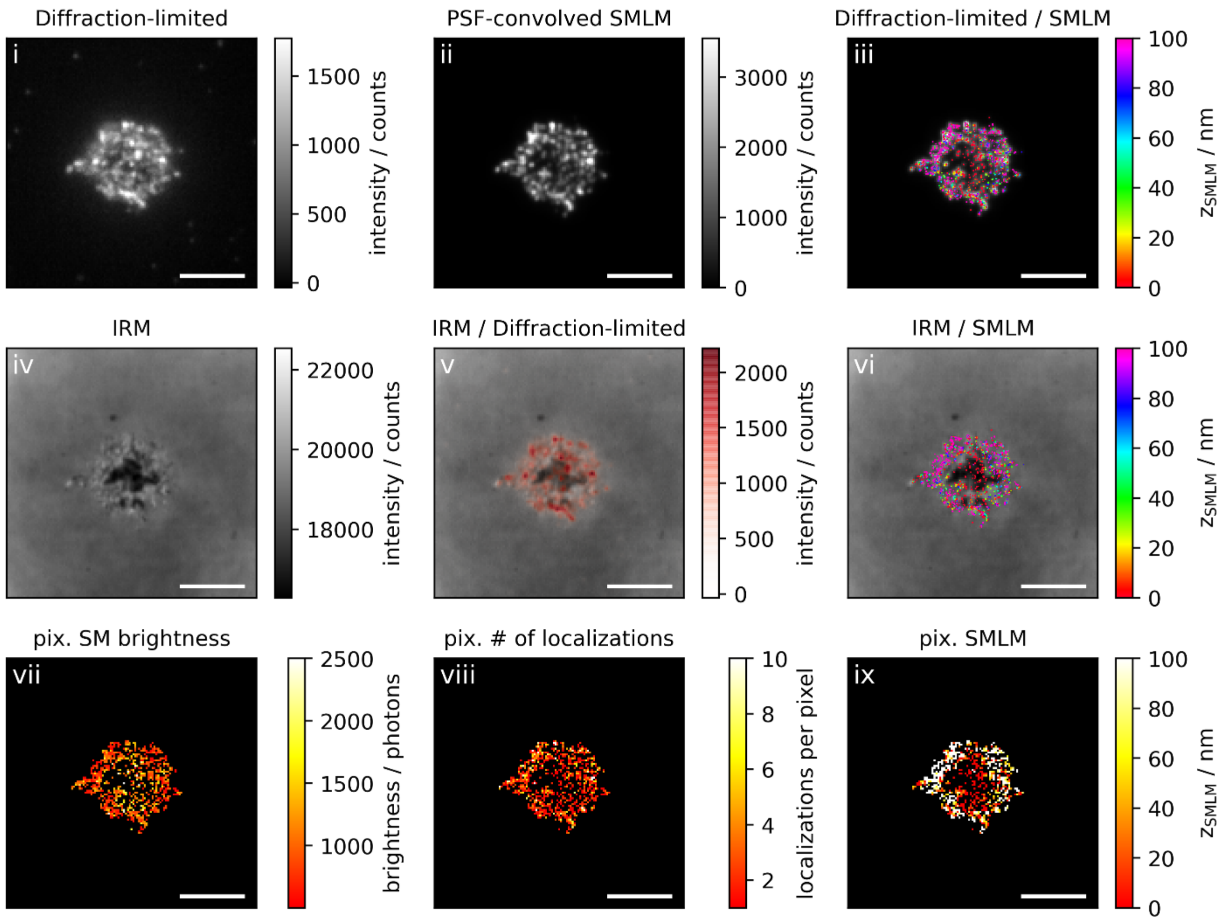
Diffraction-limited
i
intensity / counts
1500
1000
500
0
PSF-convolved SMLM
ii
3000
2000
1000
0
Diffraction-limited / SMLM
iii
100
80
60
40
20
0
zSMLM / nm
IRM
iv
22000
20000
18000
IRM / Diffraction-limited
v
2000
1500
1000
500
IRM / SMLM
vi
pix. SM brightness
vii
2500
2000
1500
1000
brightness / photons
pix. # of localizations
viii
10
8
6
4
2
localizations per pixel
pix. SMLM
ix

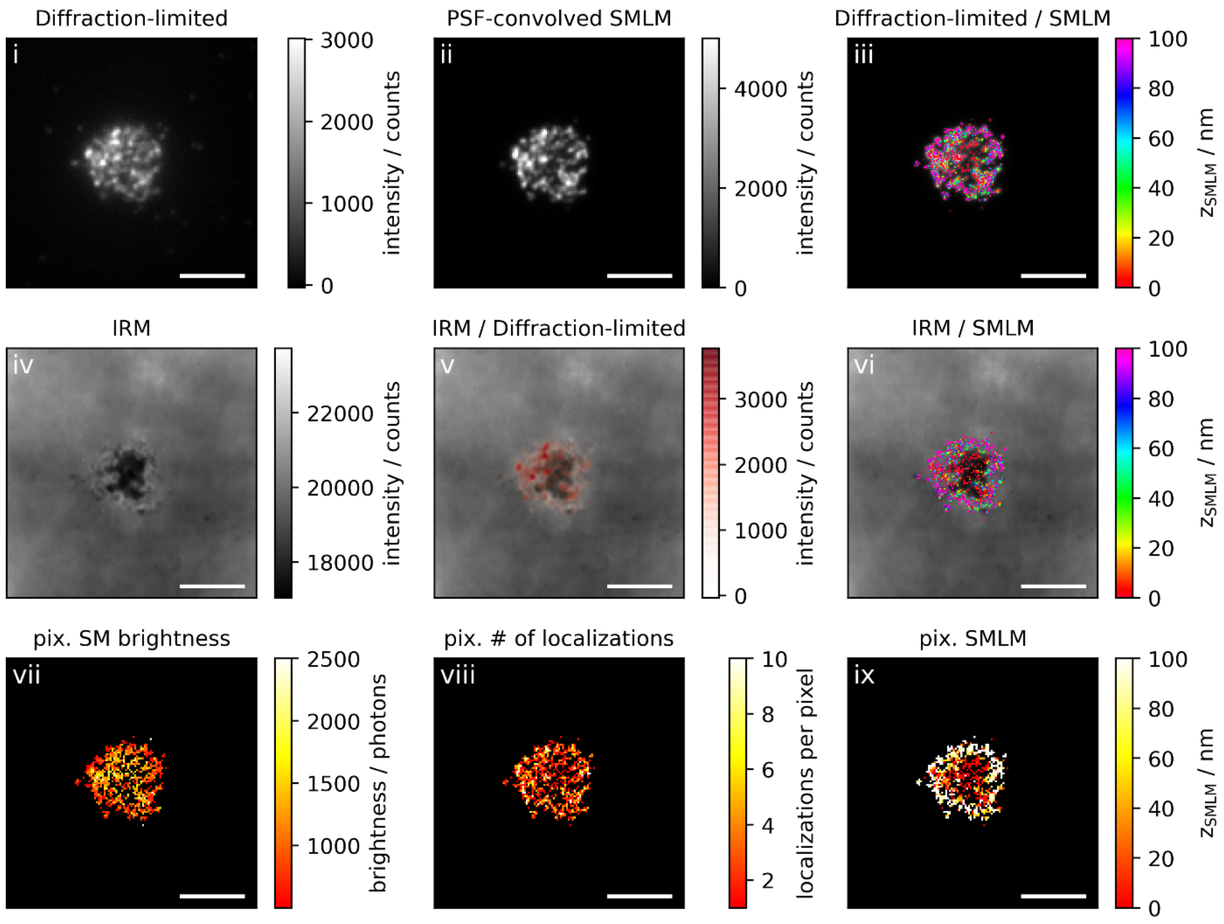

Diffraction-limited
i
3000
2000
1000
0
intensity / counts
PSF-convolved SMLM
ii
4000
2000
0
intensity / counts
Diffraction-limited / SMLM
iii
100
80
60
40
20
0
z_{SMLM} / nm
IRM
iv
22000
20000
18000
intensity / counts
IRM / Diffraction-limited
v
3000
2000
1000
0
intensity / counts
IRM / SMLM
vi
100
80
60
40
20
0
z_{SMLM} / nm
pix. SM brightness
vii
2500
2000
1500
1000
brightness / photons
pix. # of localizations
viii
10
8
6
4
2
localizations per pixel
pix. SMLM
ix
100
80
60
40
20
0
z_{SMLM} / nm

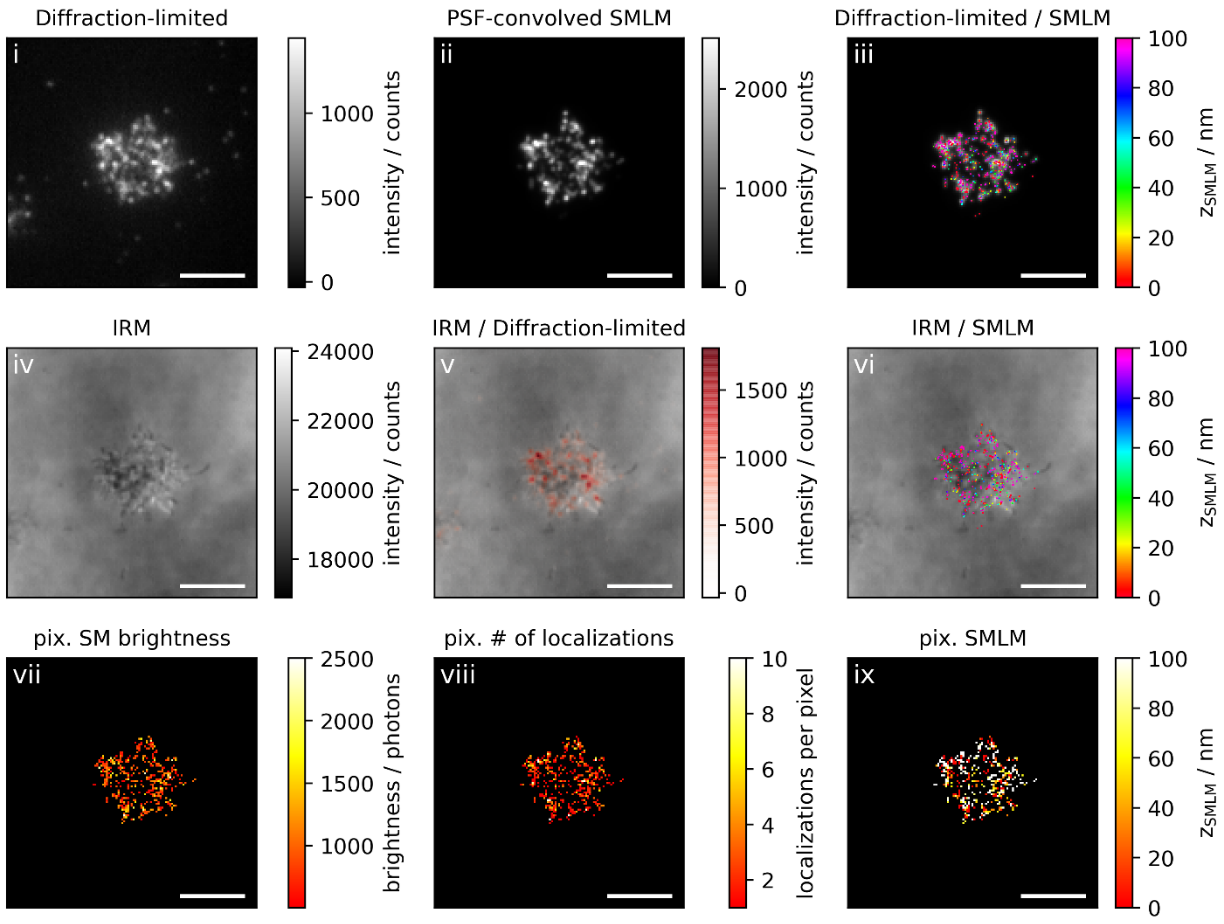
Diffraction-limited
i
1000
500
0
intensity / counts
PSF-convolved SMLM
ii
2000
1000
0
intensity / counts
Diffraction-limited / SMLM
iii
100
80
60
40
20
0
zSMLM / nm
IRM
iv
24000
22000
20000
18000
intensity / counts
IRM / Diffraction-limited
v
1500
1000
500
0
intensity / counts
IRM / SMLM
vi
100
80
60
40
20
0
zSMLM / nm
pix. SM brightness
vii
2500
2000
1500
1000
brightness / photons
pix. # of localizations
viii
10
8
6
4
2
localizations per pixel
pix. SMLM
ix
100
80
60
40
20
0
zSMLM / nm

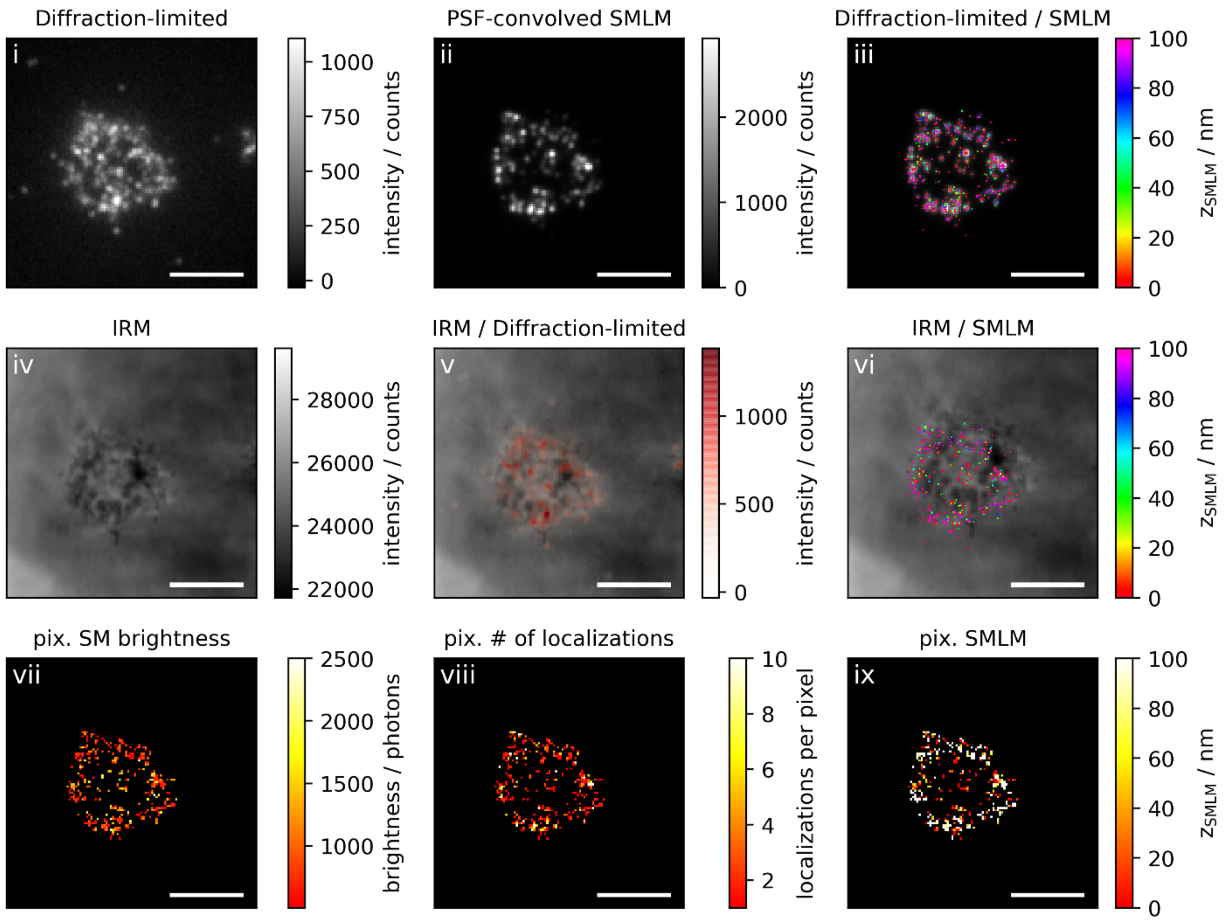

Diffraction-limited
i
intensity / counts
PSF-convolved SMLM
ii
Diffraction-limited / SMLM
iii
z_{SMLM} / nm
IRM
iv
IRM / Diffraction-limited
v
IRM / SMLM
vi
pix. SM brightness
vii
brightness / photons
pix. # of localizations
viii
localizations per pixel
pix. SMLM
ix

Figure 4c: Non-activating conditions, low ICAM-1 density, fixation: 10-15 min post seeding

Correlative 3D-SMLM, IRM, and diffraction-limited TIR microscopy of the immunological synapse. T cells were seeded on an SLB functionalized with low density of ICAM-1, and fixed 10-15 minutes post seeding. The T cell was imaged with IRM and fluorescence microscopy: (i) Diffraction-limited TIR image of the T cell. (ii) Reconstruction of the diffraction-limited image by convolving the 3D-SMLM image with the corresponding psf. (iii) Overlay of the diffraction-limited TIR image with the 3D-SMLM image. Color-code indicates distance to the coverslip z_{SMLM}. (iv) IRM image. (v) Overlay of the IRM image with the diffraction-limited image. (vi) Overlay of the IRM and the 3D-SMLM image. Bottom row images were generated by calculating the pixel-wise average of the 3D-SMLM images (pixel size of 146nm is consistent with diffraction-limited image) according to pixelated mean single molecule (SM) intensity (vii), pixelated number of localizations (viii) and pixelated mean z_{SMLM} (ix). Scale bars 5 µm.

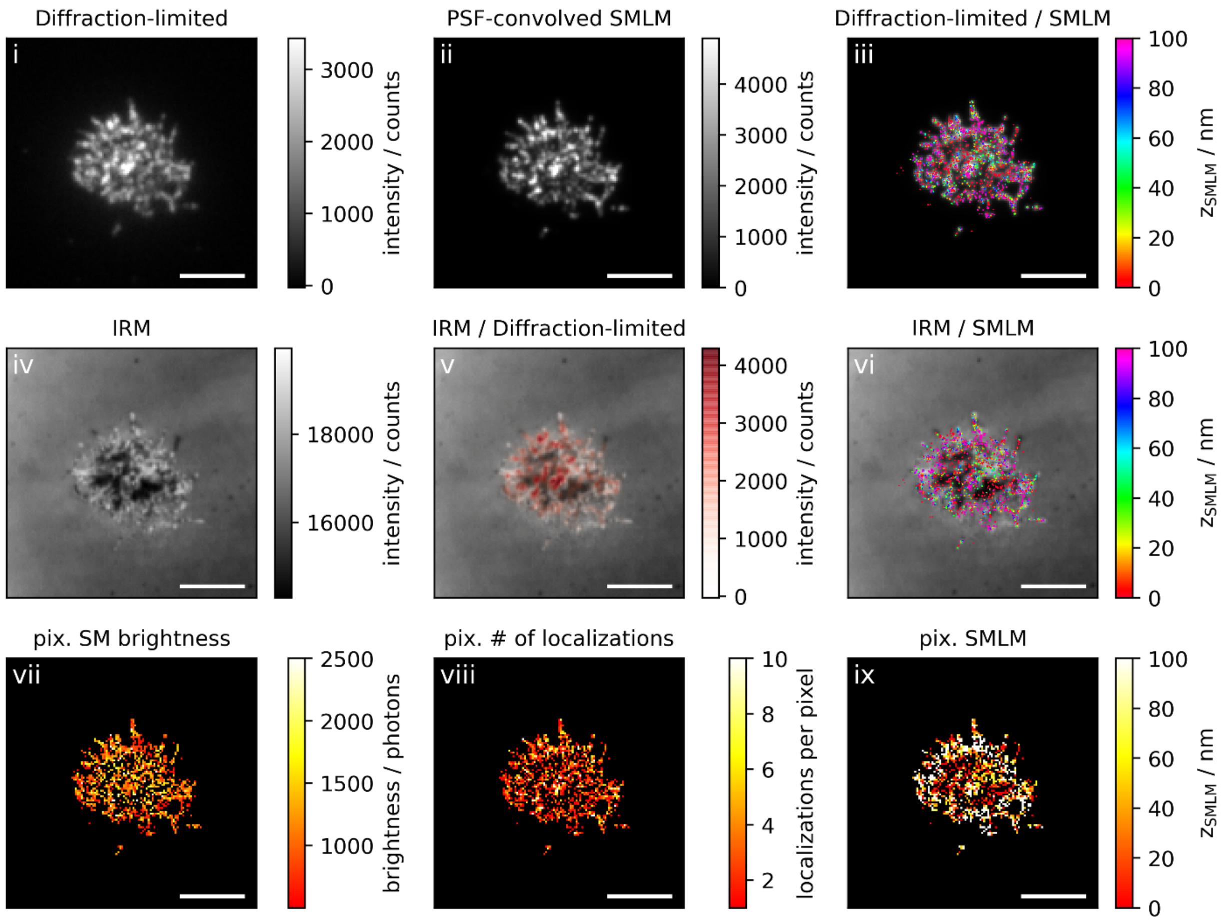
Diffraction-limited
i
intensity / counts
PSF-convolved SMLM
ii
Diffraction-limited / SMLM
iii
z_{SMLM} / nm
IRM
iv
IRM / Diffraction-limited
v
IRM / SMLM
vi
pix. SM brightness
vii
brightness / photons
pix. # of localizations
viii
localizations per pixel
pix. SMLM
ix

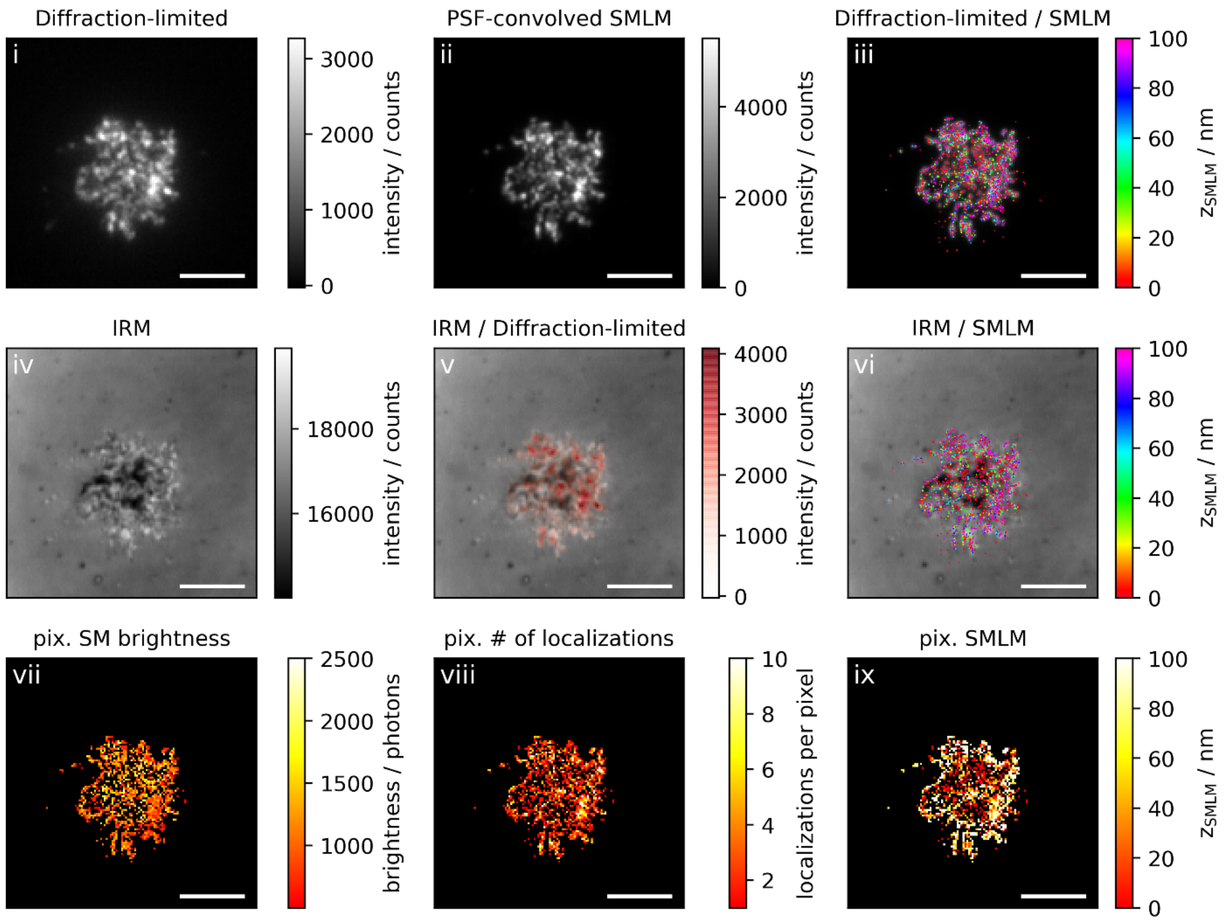
Diffraction-limited
i
3000
2000
1000
0
intensity / counts
PSF-convolved SMLM
ii
4000
2000
0
intensity / counts
Diffraction-limited / SMLM
iii
100
80
60
40
20
0
z_{SMLM} / nm
IRM
iv
18000
16000
intensity / counts
IRM / Diffraction-limited
v
4000
3000
2000
1000
0
intensity / counts
IRM / SMLM
vi
100
80
60
40
20
0
z_{SMLM} / nm
pix. SM brightness
vii
2500
2000
1500
1000
brightness / photons
pix. # of localizations
viii
10
8
6
4
2
localizations per pixel
pix. SMLM
ix
100
80
60
40
20
0
z_{SMLM} / nm

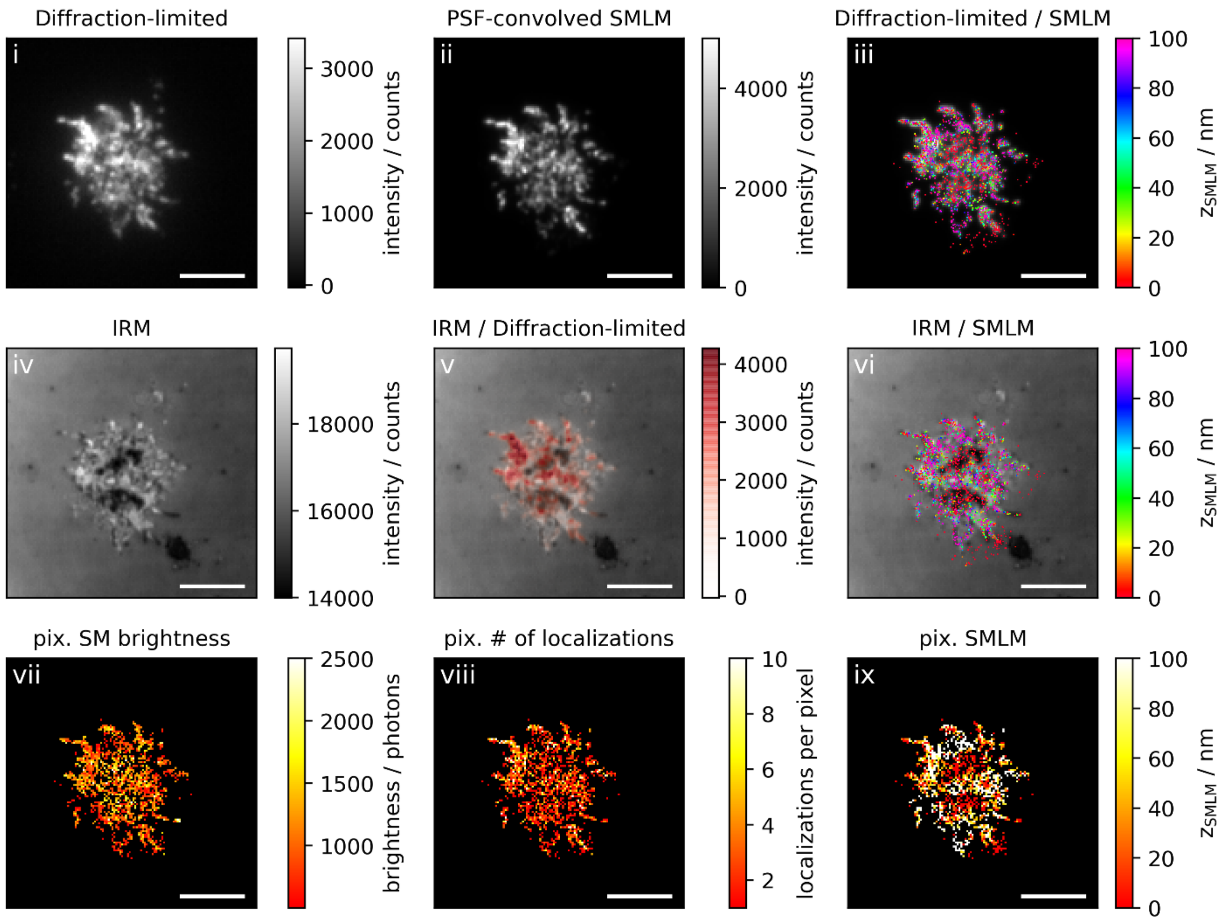

Diffraction-limited
i
intensity / counts
PSF-convolved SMLM
ii
Diffraction-limited / SMLM
iii
z_{SMLM} / nm
IRM
iv
IRM / Diffraction-limited
v
IRM / SMLM
vi
pix. SM brightness
vii
brightness / photons
pix. # of localizations
viii
localizations per pixel
pix. SMLM
ix

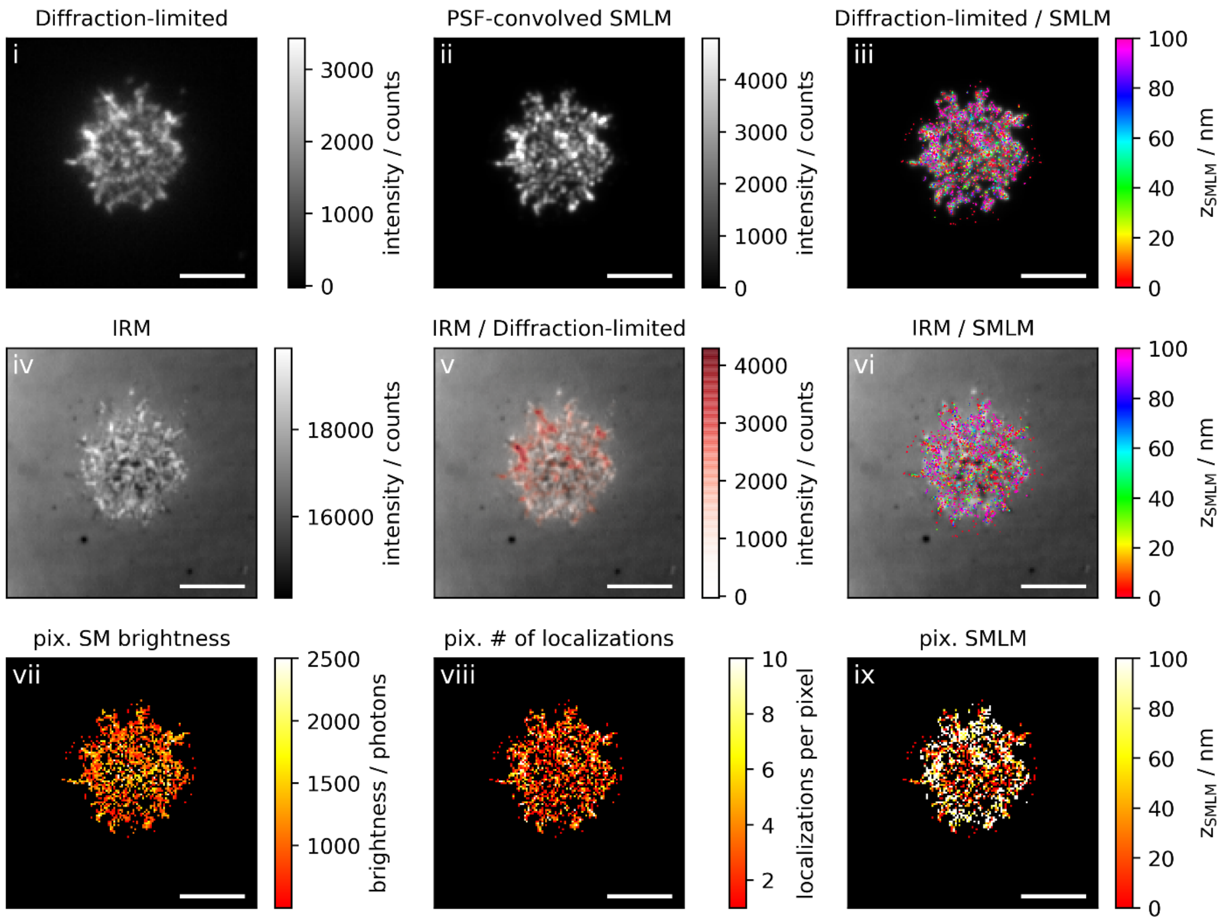
Diffraction-limited
i
3000
2000
1000
0
intensity / counts
PSF-convolved SMLM
ii
4000
3000
2000
1000
0
intensity / counts
Diffraction-limited / SMLM
iii
100
80
60
40
20
0
z_{SMLM} / nm
IRM
iv
18000
16000
intensity / counts
IRM / Diffraction-limited
v
4000
3000
2000
1000
0
intensity / counts
IRM / SMLM
vi
100
80
60
40
20
0
z_{SMLM} / nm
pix. SM brightness
vii
2500
2000
1500
1000
brightness / photons
pix. # of localizations
viii
10
8
6
4
2
localizations per pixel
pix. SMLM
ix
100
80
60
40
20
0
z_{SMLM} / nm

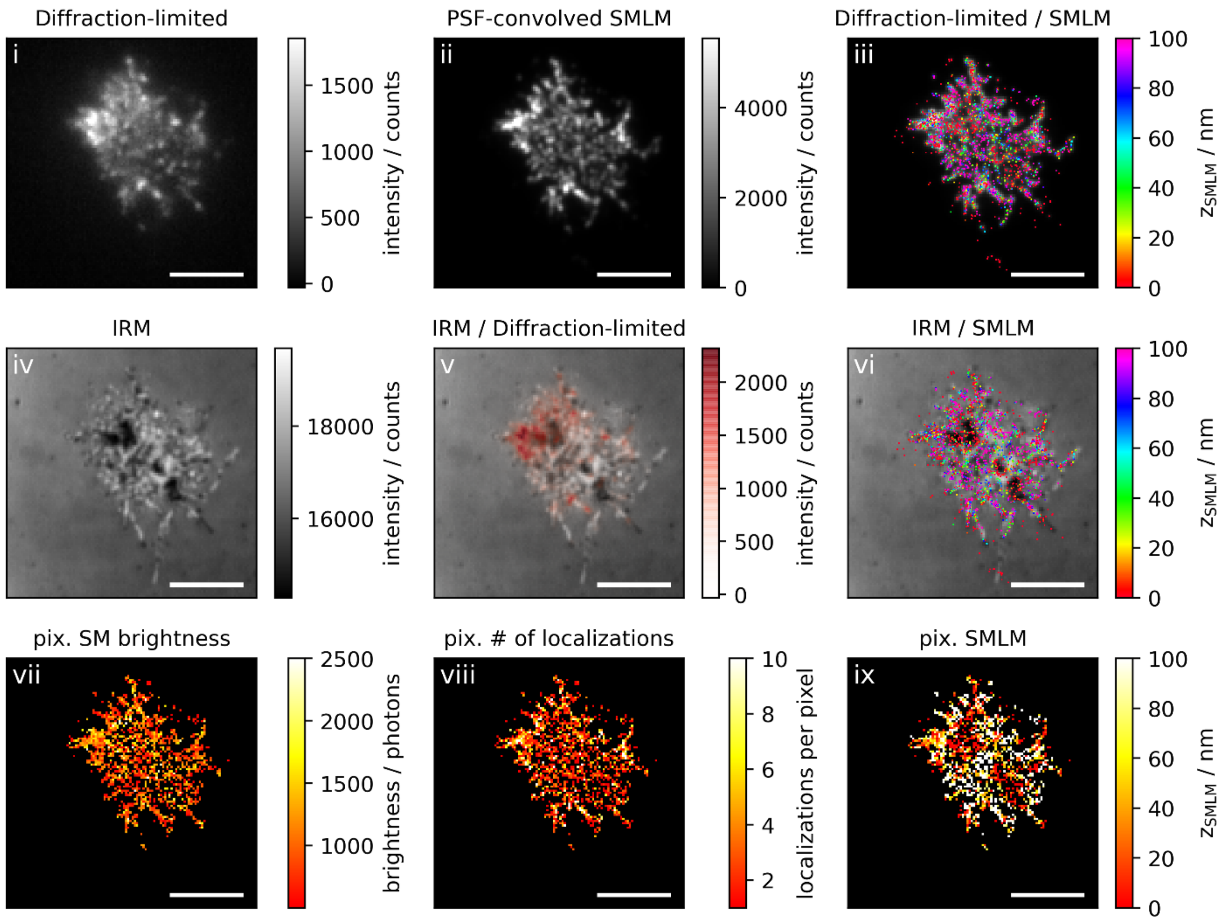

Diffraction-limited
i
intensity / counts
1500
1000
500
0
PSF-convolved SMLM
ii
4000
2000
0
intensity / counts
Diffraction-limited / SMLM
iii
100
80
60
40
20
0
z_{SMLM} / nm
IRM
iv
18000
16000
intensity / counts
IRM / Diffraction-limited
v
2000
1500
1000
500
0
intensity / counts
IRM / SMLM
vi
100
80
60
40
20
0
z_{SMLM} / nm
pix. SM brightness
vii
2500
2000
1500
1000
brightness / photons
pix. # of localizations
viii
10
8
6
4
2
localizations per pixel
pix. SMLM
ix
100
80
60
40
20
0
z_{SMLM} / nm
